# Exploring the Effects of Se Basicity on a Te···Se Interaction Supported by a Rigid Indazolium Backbone

**DOI:** 10.1021/acs.organomet.4c00094

**Published:** 2024-05-29

**Authors:** Logan T. Maltz, François P. Gabbaï

**Affiliations:** Department of Chemistry, Texas A&M University, College Station, Texas 77843, United States

## Abstract

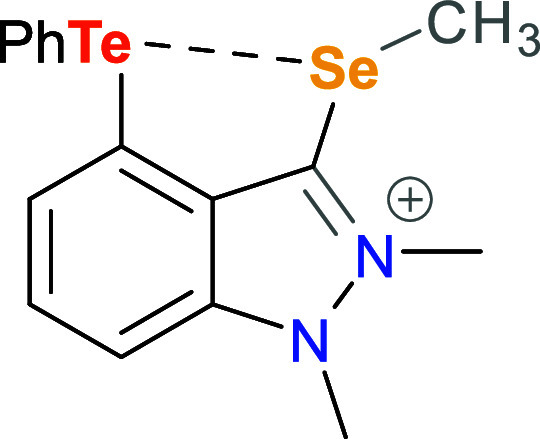

With an interest in chalcogen bonding, we use a rigid indazolium backbone to install a formally zero-valent Se center next to a divalent Te center, allowing us to investigate the effects of oxidation of the Se center on the observed Te···Se interaction. Through spectroscopic and computational comparison of the Se(0) species with its Se(II) counterpart and their monochalcogen analogues, we experimentally and computationally investigate the effect of modulating Se basicity on the resulting Te···Se interaction. Comparison with well-studied naphthalene and acenaphthene variants indicates that the increased basicity of the Se(0) center allows for a comparably strong Te···Se interaction despite longer *peri* distances and a larger splay angle. Finally, our study illuminates the potential non-innocence of cationic organic substituents in chalcogen-bonding catalysis of the transfer hydrogenation of quinolines.

## Introduction

Non-covalent interactions. While the prefix “non” makes us think about what they are not, these interactions are *not* unimportant; instead, they are foundational to every branch of chemistry ranging from supramolecular chemistry^1−3^ to catalysis^4−7^ and even to anion binding^8,9^ and transport,^10−12^ just to name a few. A recurring theme in this broad field is the interaction between an electron-rich region and an electron-poor one. This distinction immediately brings to mind Lewis acid–base interactions which can exhibit non-covalent interactions, especially when the interaction becomes elongated.

Recently, there has been a surge of interest in p-block Lewis acids because of their ability to form hypervalent Lewis acid–base adducts using σ*-orbitals and their coincident σ-holes. This focus started predominantly with halogen bonds^13−15^ and progressively worked its way to the left of the periodic table, extending to chalcogen bonds, pnictogen bonds, and even tetrel bonds.^16^ With their use of empty σ*-orbitals, these compounds have challenged the notion that the interactions they form are purely “non-covalent.”^17^ While the Gabbaï lab has a strong tradition with pnictogen-based Lewis acids,^18−20^ we—like others—have become enamored with the neighboring chalcogens.^1−12^

Chalcogen atoms can assume a divalent, tetravalent, or hexavalent state. The divalent state is especially interesting because the chalcogen center seems perfectly ambiphilic, balancing two Lewis acidic σ-holes/σ*-orbitals with two Lewis basic lone pairs. This tension between acting as a Lewis base or a Lewis acid adds intrigue to their dinuclear interactions. Many groups have studied whether the formation of a non-covalent interaction between chalcogens can be induced by forcing two chalcogen centers together.

**Figure 1:**
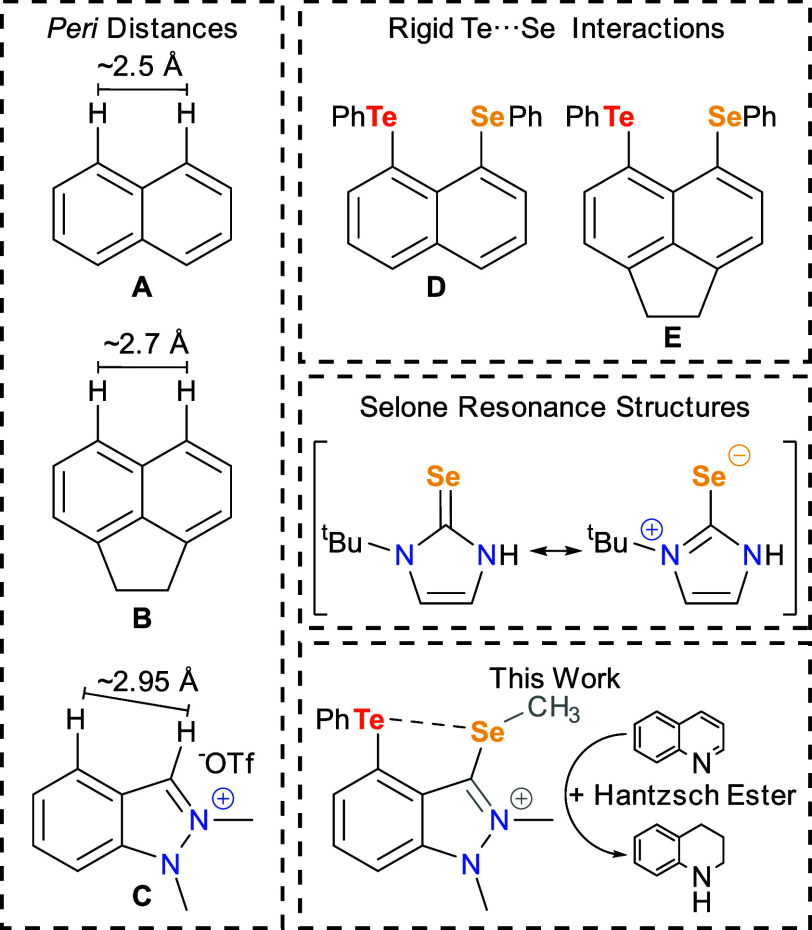
Left: *Peri* distances in rigid backbones. Right (top): Close Te···Se contacts enforced by rigid backbones. Right (middle): Selected resonance structures of selones.^26^ Right (bottom): Visual summary of work covered.

Accordingly, homonuclear and heteronuclear dichalcogen species have been investigated on rigid naphthalene and acenaphthene backbones over the course of several decades.^21−24^ Naphthalene provides a *peri* distance of ∼2.5 Å while acenaphthene allows for controlled elongation of the interatomic interactions with a slightly longer *peri* distance of ∼2.7 Å (Figure 1).^24^ These dichalcogen systems have been extensively studied on naphthalene,^21,22,25^ and it has been shown that most of the systems exhibit 3c-4e chalcogen bonding.^21,25^ In heteronuclear systems, the less electronegative element in the pair serves as the chalcogen-bond donor (i.e., the Lewis acid) and thus receives electron density from the more electronegative atom.^25^

The mixed Te/Se systems are particularly interesting because of the defined roles of Te as Lewis acid and Se as Lewis base. Oxidation of the Te center has been observed to increase the strength of its interaction with Se.^27^ While methylation is a common way to augment Lewis acid–base interactions, it typically involves oxidation from the +2 to the +4 state and focuses on increasing Lewis acidity.^28^ We often focus on the Lewis acid, but what about altering the basicity of Se in these interactions?

Herein, we modulate the basicity of Se and probe its interaction with divalent Te. This investigation is facilitated by the 1,2-dimethylindazolium backbone. Like naphthalene and acenaphthene, this backbone is rigid and planar, yet it provides a slightly longer *peri* distance of ∼2.95 Å and access to a σ-donating carbene center.^29^ Combined with Se, this carbene allows access to the zwitterionic resonance structures of selones which place a partially negative charge on the Se center (Figure 1).^26^

With this new backbone, we synthesize a series of mixed Te/Se species and their monochalcogen analogues, employing spectroscopic and computational analyses to investigate the difference in the Te···Se interaction upon oxidizing Se from the formally 0 state to the +2 state. We then turn our attention to comparing these systems with the previously published naphthyl (**D**) and acenaphthyl (**E**) derivatives bearing divalent Te and Se centers. Finally, we investigate whether the synthesized dichalcogens can serve as catalysts in the transfer hydrogenation of quinoline.

## Results and Discussion

### Synthesis and Analysis

**Figure 2:**
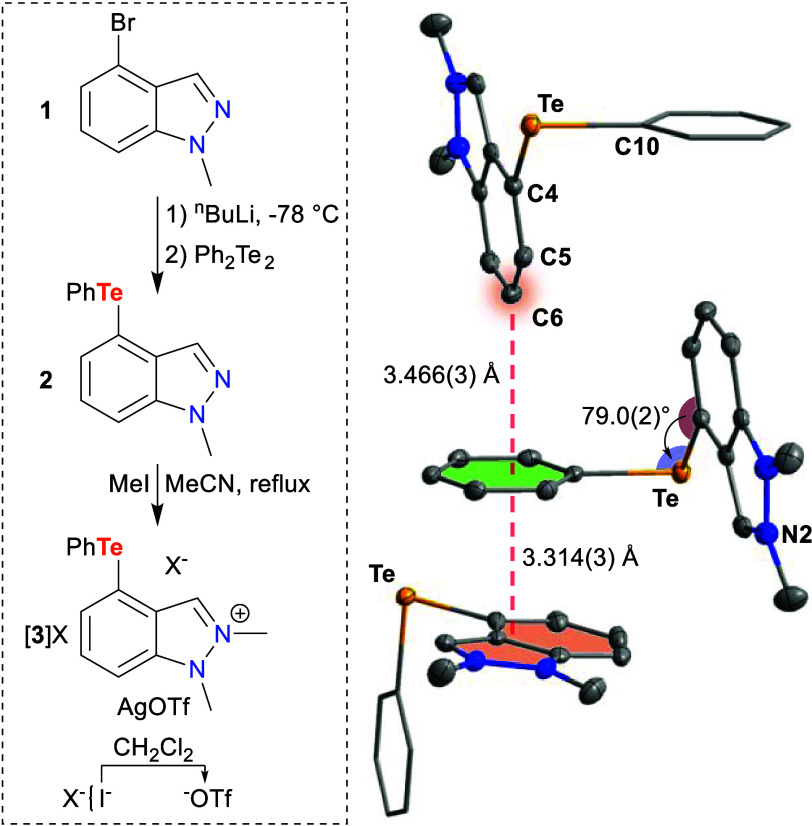
Left: Reaction scheme for synthesis of [**3**]X from **1**. Right: Crystal structure of [**3**]I highlighting intermolecular interactions with selected metrics. Hydrogen atoms and counterion I^–^ omitted for clarity. Thermal ellipsoids drawn at 50% probability; phenyl groups drawn as thin lines.

We started our synthesis by adding a divalent Te center to the neutral indazole precursor, leaving the carbene position open for further functionalization. Similar to our previous work with the indazole/indazolium backbone,^30,31^ we added ^n^BuLi to a solution of 4-bromo-1-methyl-1*H* indazole (**1**) in dry tetrahydrofuran (THF) at −78 °C. After stirring for 2 h, 1 equiv of Ph_2_Te_2_ was added to the lithium salt. The resulting solution was stirred overnight and worked up using two sequential silica chromatography columns, yielding **2** as an amber oil (Figure 2). ^125^Te NMR spectroscopy clearly indicated the installation of the Te with a peak at 600.3 ppm (Table 1). The integrations in the ^1^H NMR spectrum in addition to the singlet at 7.86 ppm—characteristic of the indazole C-*H*—further validated the successful synthesis of **2**.

**Table 1:** Experimental ^77^Se and ^125^Te Chemical Shifts in CDCl_3_

Compound	δ_Se_ (ppm)	δ_Te_ (ppm)
**2**		600.3
[**3**]OTf		652.8
**4**	204.2	656.7
**5**	90.8	679.8
**7**	131.0	
**8**	103.3	

To acidify the protocarbene, we added a cationic charge to the system by methylating **2**. This methylation was easily accomplished by refluxing **2** with an excess of MeI in acetonitrile (MeCN) for 3 days to produce indazolium [**3**]I as a pale-yellow powder (Figure 2). Despite the harsh conditions, the Te center remained untouched as indicated by the lack of Te satellites in the ^1^H NMR spectrum. Due to its poor solubility in CDCl_3_, [**3**]I was characterized in CD_3_CN (Supporting Information); however, the salt was sufficiently soluble in CDCl_3_ to obtain a satisfactory ^1^H NMR spectrum of [**3**]I for a more straightforward comparison with **2**. The introduction of a cationic charge shifted the indazolium C-*H* singlet significantly downfield to 8.92 ppm in the ^1^H NMR spectrum. The ^1^H NMR spectrum further depicted the monomethylation of **2** with two distinct methyl resonances at 4.77 and 4.51 ppm.

Single-crystal X-ray diffraction (SCXRD) quality crystals were obtained by layering a solution of [**3**]I in MeCN with Et_2_O (Figure 2). With a distance of 3.7470(12) Å between the I^–^ counterion and the plane of the π^+^-surface, there is potential for an anion–π interaction to occur.^32^ Instead of positioning itself over the center of the π^+^-surface, though, the I^–^ lies directly over the two nitrogens where the maximum electrostatic potential in the molecule is localized (*vide infra*). The phenyl group of the Te, on the other hand, seems to interact more directly with the π^+^-surface.

By orienting the Te–Ph bond orthogonally to the indazolium backbone (∠C10–Te1–C4-C5 = 79.0(2)°), two molecules can interact with each other in a face-to-edge manner between the π-surface of the phenyl and the positive C6 region of the indazolium backbone (Ph···C6 = 3.466(3) Å).^33,34^ There are also π–π interactions occurring between the phenyl of one molecule and the indazolium surface of a third molecule (Ph···C9 = 3.314(3) Å). In this way, three molecules in the crystal structure interact through the phenyl of one molecule, stabilizing the orthogonal orientation of the Te–Ph bond. This orientation is not seen in the gas-phase optimized structure (*vide infra*). Instead, the Te–Ph bond aligns with the indazolium backbone likely due to beneficial π-interactions between a Te lone pair and the backbone,^25^ the loss of which is compensated for in the solid state by these other intermolecular interactions.

To increase the solubility of **3**^**+**^ in CDCl_3_—allowing for better comparison with the other molecules—we used AgOTf to exchange the I^–^ counterion for a triflate (^−^OTf) (Figure 2). Compared with the I^–^ salt, there is a slight upfield shift in the indazolium C-*H* singlet to 8.60 ppm and in the methyl resonances to 4.47 and 4.29 ppm, but the aromatic protons remain in similar positions, potentially indicating some association of the triflate with the protocarbene C-*H*. Indicative of the conversion to a cationic indazolium, the ^125^Te signal appears at 652.8 ppm, more than 50 ppm downfield of the same resonance in indazole **2**.

**Figure 3:**
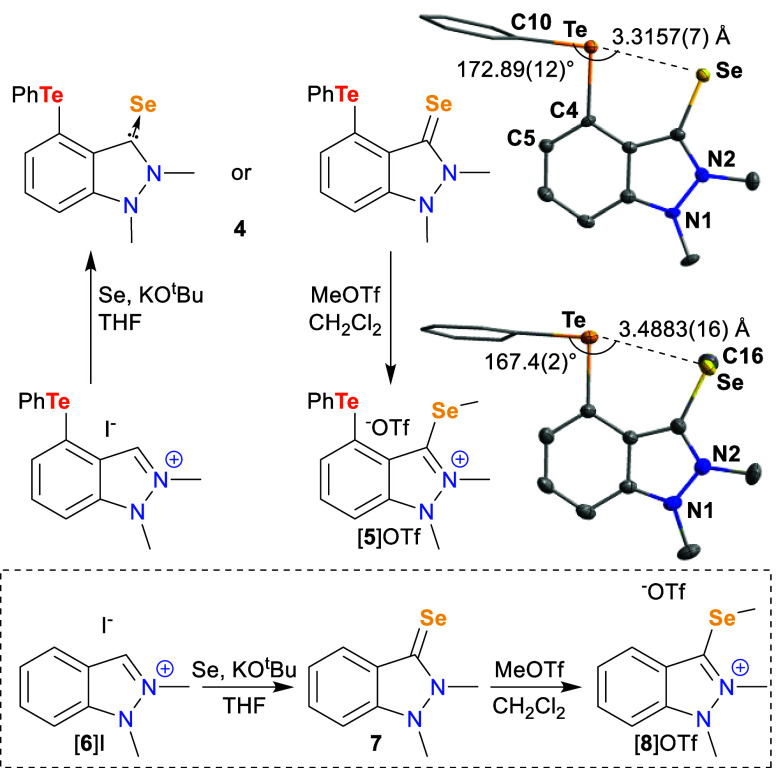
Top: Reaction scheme for synthesis of [**5**]OTf from [**3**]I and associated crystal structures of **4** and [**5**]OTf with selected metrics. Hydrogen atoms, solvent, and ^–^OTf counterion omitted for clarity. Thermal ellipsoids drawn at 50% probability; phenyl groups drawn as thin lines. Bottom: Synthesis of Te-free analogues **7** and [**8**]OTf.

Having acidified the indazolium C-*H*, we sought to deprotonate the protocarbene to install Se within the van der Waals radius of the Te center. By refluxing [**3**]I with Se powder and KO^t^Bu in THF, **4** was produced as a yellow solid with a formally Se(0) center supported by a C → Se dative interaction (Figure 3). The disappearance of the protocarbene C-*H* resonance from the ^1^H NMR spectrum indicated the successful coordination of the carbene to Se. With backdonation from this Se into the carbene, the methyl peaks were shielded, shifting upfield relative to the salts of **3**^**+**^.

Being NMR-active itself, Se provides another handle through which to probe the molecule. Compared to the ^77^Se chemical shift of 131.0 ppm for Te-free analogue **7**—synthesized similarly to **4** (Figure 3)—the Se resonance in **4** shifts significantly downfield to 204.2 ppm, potentially indicating an interaction with the Te center.^36^ The ^125^Te NMR spectrum does not see a significant change, with only a slight downfield shift of about 4 ppm from [**3**]OTf to 656.7 ppm accompanying the new compound’s formation. The difference in the relative shifts of the ^77^Se and ^125^Te chemical shifts may indicate that alteration of the backbone’s electronic structure plays a more significant role in the observed shifts with the Se chemical shift being more affected due to increased conjugation with the backbone.

X-ray diffraction analysis of single crystals obtained by layering hexanes over a CDCl_3_ solution of **4** provided further insight into the Te···Se interaction. The most apparent difference between the structures of **4** and [**3**]I is the Te–Ph bond aligning with the indazolium backbone to engage the Se with the Te–Ph σ*-orbital and the coincident σ-hole on Te (∠C10–Te1–C4-C5 = 2.5(4)°; ∠C10–Te1–Se1 = 172.89(12)°). While intermolecular π–π interactions do occur between the indazolium surfaces in **4**, the favorable Te···Se interaction compensates for the loss of the weak intermolecular interactions with the phenyl that were seen in [**3**]I. With the rigidity of the indazolium backbone forcing them into contact with each other, Te and Se exhibit a short interatomic distance of 3.3157(7) Å that is within the sum of the van der Waals radii for the two atoms (3.96 Å);^37^ even so, this distance seems comfortable for the molecule considering that the splay angle—the deviation in the sum of the three angles involving the *peri*-substituted atoms from those in the substituted structure^22^—is the same as that seen for unsubstituted 1,2-dimethylindazolium triflate.^29^

As previously mentioned, the Se can backdonate to the carbene through the Se=C double bond. Yet, there is a favorable resonance form in which the Se accepts electrons from this double bond, forming a single bond with the carbene carbon and assuming a negative charge. Exploiting the enhanced nucleophilicity offered by this resonance form, we methylated the formally Se(0) center to probe the effects of oxidation on the Te···Se interaction.

By adding 1 equiv of MeOTf to **4** in dichloromethane (DCM) at room temperature, we selectively methylated the Se center over the Te center. The success of the methylation was evident by the appearance of a third methyl resonance upfield at 2.64 ppm in the ^1^H NMR spectrum. This new methyl peak is a triplet with ^77^Se satellites distinguished from ^125^Te satellites by the smaller coupling constant (^2^*J*_Se–H_ = 6.2 Hz). The cationic charge induced a further downfield shift of the ^125^Te resonance (679.8 ppm) while additional electron density from the methyl on the Se caused an upfield shift in the ^77^Se NMR spectrum (90.8 ppm).^38^ Compared with the shift from **7** to [**8**]OTf (Δδ_Se_ = 27.7 ppm), the significantly larger 113.4 ppm shift from **4** to [**5**]OTf may indicate a decrease in the Te···Se interaction though the impact on the backbone’s electronic structure could also play a role as previously mentioned.

Layering hexanes over a solution of [**5**]OTf in DCM yielded SCXRD quality crystals. Their analysis indicated some level of positional disorder. Appropriate modeling led to a major component that accounted for 75% of the occupancy, and we focus our analysis on this component. The methyl adds orthogonally to the C–Te···Se angle, allowing Se to still donate electron density to the Te center; however, this interaction is now visibly weaker upon oxidation of the Se: the Te···Se distance increases by 0.17 Å to 3.4883(16) Å and the splay angle increases by ∼4°.

### Computational Investigation

While the crystal structures and NMR spectroscopy are informative, we turned to computational tools to gain a deeper insight into the Te···Se interaction in **4** and **5**^+^. Starting from the crystal geometries, the structures were optimized using the B3LYP functional with Grimme’s D3 dispersion correction with Becke–Johnson damping and a mixed basis set (Te: aug-cc-pVTZ-PP; Se: aug-cc-pVTZ; H/C/N: def2-TZVP). This level of theory correlated well with the Te···Se distances in the molecules investigated (Graph S1).

The most obvious difference between **4** and **5**^**+**^ is the presence of a positive charge, so it seems pertinent to start our analysis by looking at natural population analysis (NPA) charges (Table S1). Upon methylation of indazole **2** to indazolium **3**^**+**^, the charge on Te increases slightly from +0.51 to +0.58. The charge on the Te center does not differ much in either **4** or **5**^**+**^ compared with **3**^**+**^ (**4**: +0.59; **5**^**+**^: +0.59), though the slight increase in charge suggests some sharing of electron density with the adjacent Se center.

The Se center itself is better analyzed without Te complicating the charge, so we look to the Te-free analogues of **4** and **5**^**+**^: **7** and **8**^**+**^, respectively. Despite being formally neutral, the Se in **7** has a partial negative charge of −0.21 due to the large contribution from the zwitterionic resonance structure. Methylation leads to a dramatic change in the charge on Se to +0.38 in **8**^**+**^. With the addition of a Lewis acidic PhTe moiety next to these Se centers, we see a reduction in the magnitude of the charges as the electron density distributes itself between the two centers: for **4**, the Se charge decreases in magnitude by 0.04 to −0.17, while a smaller decrease in the Se charge is seen for **5**^**+**^ (Δ*q* = −0.01). This smaller decrease in **5**^**+**^ suggests less electron sharing between Te and Se and thus a weakening of the Te···Se interaction moving from **4** to **5**^**+**^. As previously stated, this weakening is evident in the longer Te···Se distance and larger splay angle in the crystal structure geometries; however, it is also seen in the topology of the electron density, as highlighted by an atoms-in-molecules (AIM) analysis.

**Figure 4:**
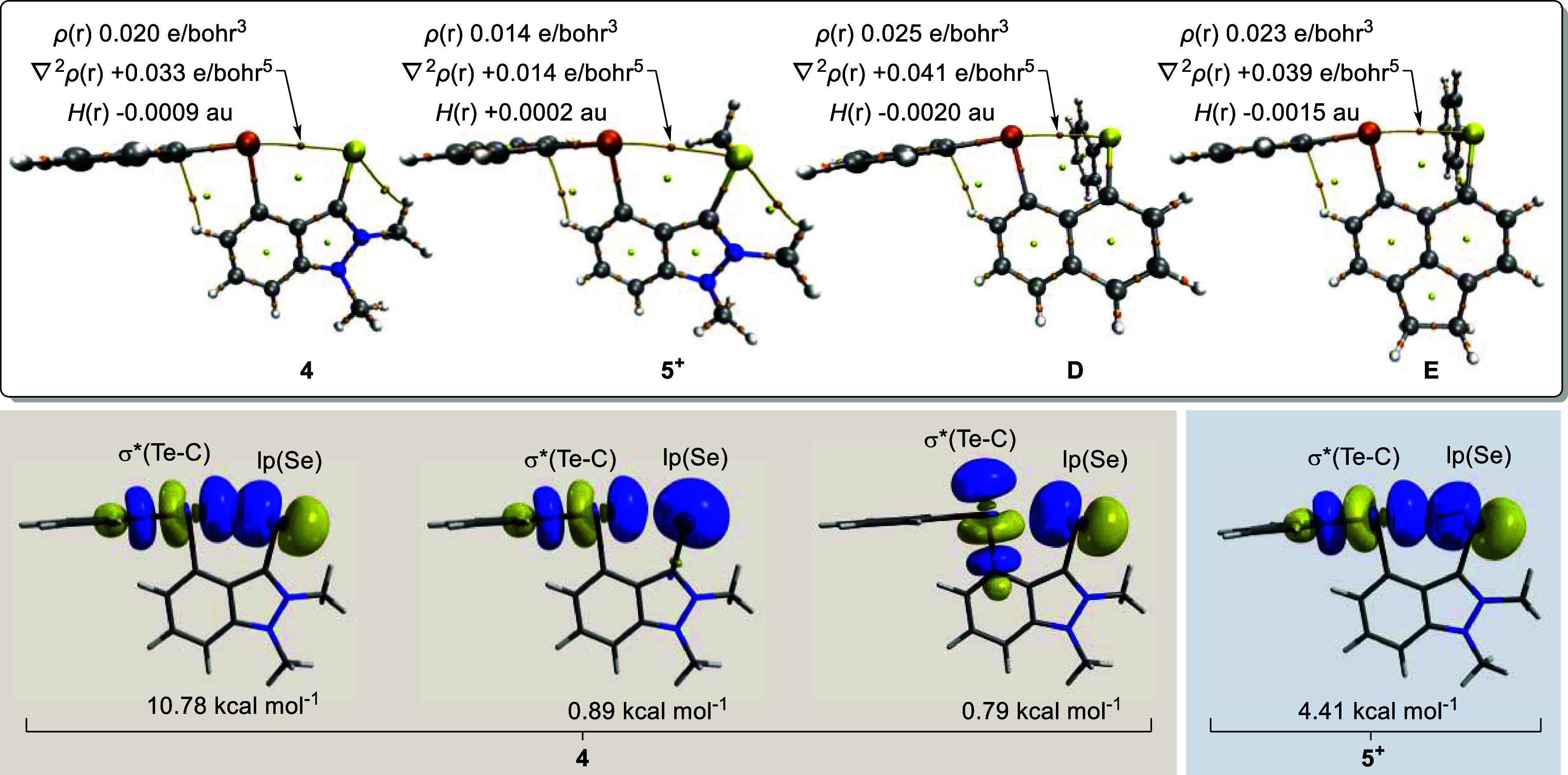
Top: AIM analysis with Te···Se bond critical point metrics indicated. Bottom: lp(Se) → σ*(Te–C) interactions identified by NBO analysis for **4** and **5**^**+**^ (isovalue = 0.05).

AIM identifies a bond path between the Te and Se centers in both **4** and **5**^**+**^ (Figure 4). The decrease in the electron density ρ(*r*) at the bond critical point from 0.020 e bohr^–3^ in **4** to 0.014 e bohr^–3^ in **5**^**+**^ is expected for a decrease in the strength of the interaction. Furthermore, while the Laplacian ∇^2^ρ(*r*) remains positive in both species—indicating a closed-shell interaction—the total energy density *H*(*r*) shifts from negative in **4** to positive in **5**^**+**^, suggesting that the interaction shifts from a partially covalent interaction to a predominantly electrostatic one.^39^

While the interaction in **5**^**+**^ might be predominantly electrostatic, natural bond orbital (NBO) second-order perturbation theory analysis does identify a lp(Se) → σ*(Te–C) interaction with a magnitude of 4.41 kcal mol^–1^ (Figure 4). This interaction is not insignificant, yet the same interaction in **4** has a magnitude of 10.78 kcal mol^–1^. Furthermore, **4** has two more Se → Te interactions: one between the p-based lone pair of Se and the σ*-orbital of Te *trans* to the indazolium backbone and the other between the s-based lone pair of Se and the σ*-orbital of Te *trans* to the phenyl (Figure 4). **5**^**+**^ does not show these other two interactions as the cationic charge stabilizes the s-based lone pair on Se and contracts the Se center’s more energetically accessible p-based orbital. These three interactions in **4** provide a total deletion energy (Δ*E*_del_)^40^ of 13.07 kcal mol^–1^, more than three times larger in magnitude than the single interaction seen in **5**^**+**^. Ultimately, we see that addition of a cationic charge decreases the strength of the Te···Se interaction by weakening the orbital contribution to the interaction. The observable effect of this weakening of the covalent contribution on the Te···Se distance and the splay angle counters the notion that non-covalent interactions are covalent-*free* interactions.

As can be seen, the cationic charge on **5**^**+**^ has other implications outside of simply converting Se(0) to Se(II). As such, it would be insightful to compare **4** with neutral Se(II) analogues, particularly naphthalene-based **D** and acenaphthene-based **E**. These molecules were optimized from their reported crystal structures^22,24^ according to the same level of theory as **4** and **5**^**+**^.

NBO analysis reveals that the same three lp(Se) → σ*(Te–C) donations seen in **4** are also seen in **E** and **D** (Figures S47 and S48). Looking at the NBO analysis, we see that despite the longer *peri* distance, **4** competes with **E** and **D**. The more basic Se in **4** leads to a slightly stronger orbital interaction than **E** (Δ*E*_del_ = 13.07 kcal mol^–1^ vs 12.88 kcal mol^–1^). Ultimately, though, the shorter distance in **D** provides the highest orbital interaction with Δ*E*_del_ = 14.90 kcal mol^–1^.

While NBO analysis speaks to selected orbital components of the interaction, we again turn to AIM analysis to get a better idea of the electron density as a whole. As can be seen in Figure 4, **D** shows the largest electron density ρ(*r*) along the Te···Se bond path with a value of 0.025 e bohr^–3^ with slightly lower values of 0.023 e bohr^–3^ for **E** and 0.020 e bohr^–3^ for **4**. This result seemingly indicates that the enforcement of shorter distances does indeed promote stronger interactions. Even so, the fact that the Te···Se interaction in **4** still competes with those in **D** and **E** despite the substantial increase in the *peri* distance and the increased splay angle demonstrates the enhanced basicity of the Se(0) center compared with the Se(II) centers.

### Catalysis

Having investigated the Te···Se interaction, we wanted to test the catalytic potential of our newly synthesized molecules. Intermolecular chalcogen-bond catalysis is relatively young with the first example from the Matile group only appearing within the past decade.^5,41^ In 2017, the Huber group introduced a series of catalysts containing alkylated selenoureas for the activation of C–X bonds.^6,42^ The Gabbaï group has repeatedly demonstrated the catalytic benefits of adding a cationic charge to originally neutral systems with a 2021 publication by Zhou and Gabbaï providing an example of methylated telluroniums showing a marked increase in catalytic activity compared to their neutral tellurium congeners.^28^ These results were further supported by a simultaneous publication from the Pale group.^43^ Against this backdrop, we saw [**5**]OTf with its cationic charge and methylated selone and were eager to observe its catalytic activity.

Compared to **4**, we envisioned [**5**]OTf being more active as a result of the cationic charge increasing the Lewis acidity both by increasing the charge of the molecule and by freeing the Te and Se centers from interacting with each other. To test this hypothesis, we employed our compounds as catalysts in the commonly used benchmark reaction of the transfer hydrogenation of quinolines using Hantzsch ester (**HEH**) as a proton/hydride source (Table 2).^4,5,28^ To avoid any potential interference from water, we loaded the catalytic reaction mixtures in a nitrogen-filled glovebox using dry CDCl_3_. Due to the limited solubility of **HEH** in CDCl_3_, the reaction mixtures were constantly shaken to ensure as much homogeneity as possible.

**Table 2:**
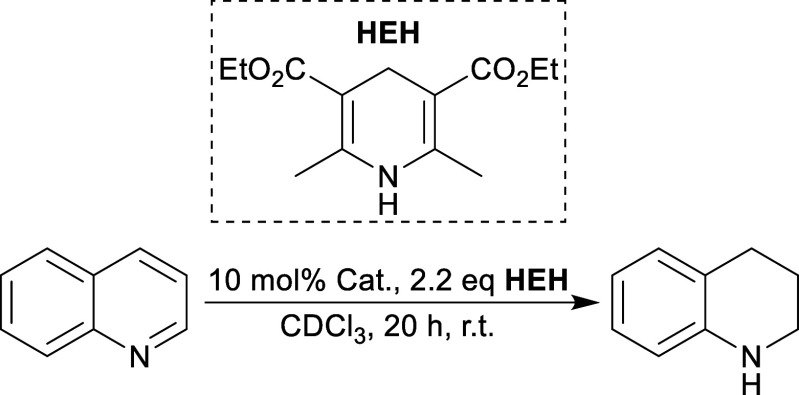
Transfer Hydrogenation of Quinoline

Entry	Cat.	Conversion (%)^a^
1	—	9
2	[**3**]OTf	95
3	**4**	3
4	[**5**]OTf	71
5	[**6**]OTf (**C**)	98
6	**7**	8
7	[**8**]OTf	83

a Conversion determined by ^1^H NMR

Gratifyingly, methylation of **4** to [**5**]OTf led to a dramatic increase in reactivity (Table 2, Entries 3 and 4). Wanting to determine whether this increase was due to the combined activity of two chalcogen bonds, we compared [**5**]OTf against Se-free [**3**]OTf and Te-free [**8**]OTf (Entries 2 and 7). Somewhat less gratifyingly, the catalytic activity increased by more than 10% compared with [**5**]OTf, reaching 95% for [**3**]OTf. This result seemingly indicated that one chalcogen center was better than two as it avoided increased steric hindrance. As would be expected, we see greater catalytic activity for Te than Se with [**3**]OTf being more active than [**8**]OTf. At the end of these reactions, however, we noticed that the Te-containing species left a small amount of dark powdery precipitate in the bottom of the reaction mixture which indicated that there might be some decomposition of compounds [**3**]OTf, **4**, and [**5**]OTf due to cleavage of the weaker Te–C bond. This result prompted us to question whether the molecules obtained upon shedding the Te atom were the active species.

**Figure 5:**
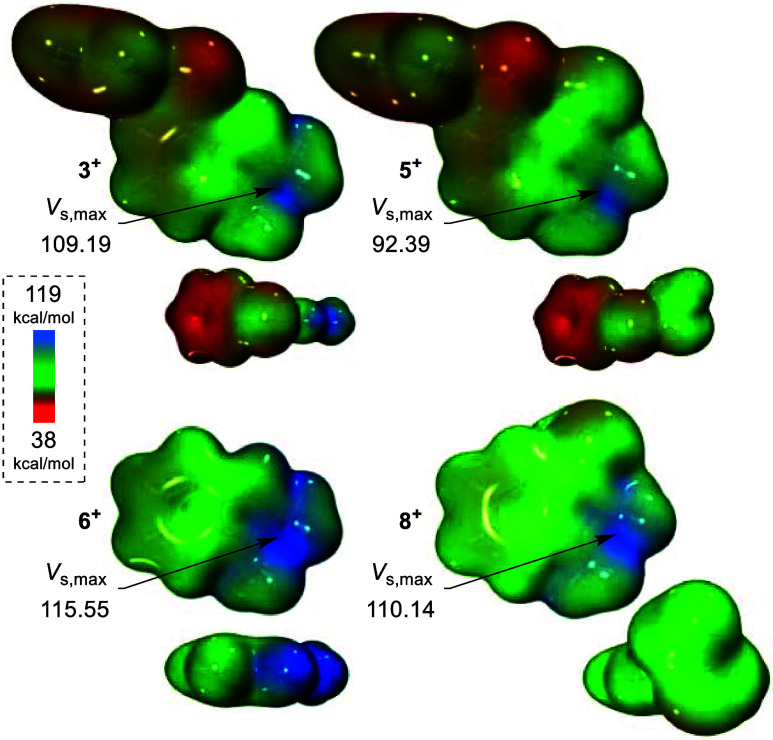
ESP maps of cationic molecules investigated with an isosurface value of 0.001 au. *V*_s,max_ values given in kcal mol^–1^. The side profile and overhead view are shown together to visualize the σ-holes on the chalcogen centers: *V*_s,max_(**3**^**+**^, Te) = 73.46 kcal mol^–1^; *V*_s,max_(**5**^**+**^, Te) = 70.24 kcal mol^–1^; *V*_s,max_(**5**^**+**^, Se) = 87.81 kcal mol^–1^; and *V*_s,max_(**8**^**+**^, Se) = 90.01 kcal mol^–1^.

We have already discussed the increased catalytic activity of Te-free [**8**]OTf compared to [**5**]OTf, seemingly supporting this hypothesis. We then compared [**3**]OTf with the completely chalcogen-free analogue [**6**]OTf. Sadly for our story of chalcogen-bond catalysis, [**6**]OTf promoted nearly complete conversion of quinoline within the observed time period (Entry 5). This data seemingly indicates that the Te-based compounds might become catalytically active upon loss of the Te center with their lower catalytic activity in comparison to the Te-free systems being explained by the time necessary to shed Te. Even so, the 83% conversion seen by Te-free [**8**]OTf indicates other factors at play. Ultimately, we turn to electrostatic potential (ESP) maps and the maximum electrostatic potential (*V*_s,max_) for a possible answer (Figure 5).

A 2019 computational investigation by Ser et al. suggested that halogen-bond catalysts promote this transfer hydrogenation by stabilizing the conjugate base of **HEH** obtained after the initial proton transfer from **HEH** to quinoline.^44^ In a similar way, we can envision cationic [**6**]OTf facilitating this protonation by stabilizing the **HEH** conjugate base through strengthened ion pairing. Accordingly, we ascribe the observed catalytic activity of [**6**]OTf to its comparatively high *V*_s,max_ of 115.55 kcal mol^–1^ (Figure 5). This value is 5–6 kcal mol^–1^ greater than the *V*_s,max_ in the less active species [**3**]OTf and [**5**]OTf. This increased charge is likely due to a combination of more focused charge on a smaller molecule and lack of electron donation from other substituents. With the lowest *V*_s,max_ in the cationic systems, [**5**]OTf also has the lowest conversion. Furthermore, the neutral species having *V*_s,max_ values less than 35 kcal mol^–1^ would explain their apparent inactivity in catalysis. Interestingly, in all of the systems, the *V*_s,max_ at the chalcogen centers is significantly less than that between the indazolium nitrogens.

Ultimately, this result reminds us to consider the potential reactivity of the organic substituents in chalcogen-bond catalysts to ensure that any observed catalytic activity is actually due to the chalcogen center. It also suggests that this transfer hydrogenation may simply be supported by common cations, the only barrier being solubility. This possibility would make for an informative future study.

## Conclusions

In this investigation, we installed divalent Te and zero-valent Se in close proximity on an indazolium backbone to promote a Te···Se chalcogen-bonding interaction. To understand the effect of Se basicity on the interaction, we methylated the Se(0) center to afford a divalent Se and physically and computationally observed the resulting weakening of the Te···Se interaction. Computational methods allowed us to compare our neutral, zero-valent Se compound with the well-studied neutral naphthyl and acenaphthyl mixed divalent Te/Se variants. Ultimately, it seemed that the increased basicity of Se(0) overcame the longer distance on the rigid backbone, rivaling the interactions seen in the naphthyl and acenaphthyl variants with shorter internuclear distances. Finally, we tested our cationic Te/Se species in the catalytic transfer hydrogenation of quinoline using a Hantzsch ester, surprisingly discovering the catalytic activity of chalcogen-free 1,2-dimethylindazolium triflate.

## Experimental Section

### General Considerations

4-bromo-1-methyl-1*H*-indazole **1**,^45^ 1,2-dimethylindazolium iodide [**6**]I,^46^ 1,2-dimethylindazolium triflate [**6**]OTf,^29^ and diphenyl ditelluride^47^ were prepared according to literature procedures. All other compounds were sourced commercially and used as received. Solvents were dried by refluxing over Na/K (Et_2_O, hexanes, THF) or CaH_2_ (CH_2_Cl_2_, CH_3_CN). All other solvents were ACS reagent grade and used as received. The synthesis of all compounds was carried out under a dry N_2_ atmosphere using standard Schlenk techniques or a glovebox unless otherwise stated. Flash chromatography was performed using a Teledyne ISCO CombiFlash RF Flash Chromatography system. NMR spectra were recorded at 298.0 K on a Bruker Avance Neo 400 spectrometer (400.09 MHz for ^1^H; 376.42 MHz for ^19^F; 76.33 MHz for ^77^Se; 126.23 MHz for ^125^Te) or at 305.0 K on a Bruker Avance 500 NMR spectrometer (500.13 MHz for ^1^H; 125.77 MHz for ^13^C) equipped with an automated tuning 5 mm ^1^H/^13^C/^15^N cold probe. Chemical shifts are given in ppm. ^1^H and ^13^C signals were referenced to residual solvent signals ^1^H (CHCl_3_: 7.26 ppm; CHD_2_CN: 1.94 ppm) and ^13^C (CDCl_3_: 77.16 ppm; CD_3_CN: 1.32 ppm). The ^19^F signals were referenced using C_6_F_6_ as a secondary external standard set at −161.64 ppm vs CFCl_3_.^48^ The ^77^Se signals were referenced using Ph_2_Se_2_ as a secondary standard set at 463.0 ppm vs Me_2_Se.^49^^77^Se NMR spectra were recorded in both the presence and absence of a sealed capillary containing a CDCl_3_ solution of Ph_2_Se_2_. The ^125^Te signals were referenced using Ph_2_Te_2_ as a secondary standard set at 422.0 ppm vs Me_2_Te.^50^^125^Te NMR spectra were recorded in both the presence and absence of a sealed capillary containing a CDCl_3_ solution of Ph_2_Te_2_. Elemental analyses were performed at Atlantic Microlab (Norcross, GA).

### Crystallographic Measurements

The crystallographic measurements were performed at 110 K using a Bruker D8 QUEST diffractometer (Mo–Kα radiation, λ = 0.71073 Å) equipped with a Photon III detector. In each case, a specimen of suitable size and quality was selected and mounted onto a nylon loop. Integrated intensity information for each reflection was obtained by reduction of the data frames with either APEX3^51^ or APEX4.^52^ The semiempirical method SADABS was applied for the absorption correction.^53^ The structures were solved by intrinsic phasing (ShelXT)^54^ and refined by the full-matrix least-squares technique against F^2^ with anisotropic temperature parameters for all non-hydrogen atoms (ShelXL)^55^ using the Olex2–1.5 interface.^56^ The hydrogen atoms were placed in calculated positions and refined using a riding model approximation. Diamond4 was employed for the final data presentation and structure plots. The data has been deposited with the Cambridge Structural Database. CCDC 2338340–2338342 contain the supplementary crystallographic data for this paper.

### Computational Methods

All calculations were carried out using density functional theory as implemented in the Gaussian 16 program.^57^ All calculations were conducted with the B3LYP functional^58−61^ with Grimme’s D3 dispersion correction with Becke–Johnson damping^62^ and a mixed basis set (aug-cc-pVTZ-PP^63^ and ECP28MDF^63^ for Te; aug-cc-pVTZ^64^ for Se; def2-TZVP^65,66^ for all other atoms) starting from the crystal structure geometries where possible.^22,24,29^ All other structures were modified in GaussView 6.1.1^67^ from their parent optimized compounds before being optimized themselves. No imaginary frequencies were found for the optimized structures, confirming that a local minimum on the potential energy hypersurface had been reached in all cases. Natural bond orbital (NBO) calculations were performed using NBO 7.0 at the same level of theory.^68^ NBOs were visualized using Avogadro.^69^ Electrostatic potential (ESP) maps were plotted using the Multiwfn software package^70,71^ in conjunction with VMD software.^72,73^ Atoms-in-molecules (AIM) analyses were performed with Multiwfn using the wave functions derived from the optimized structures. These results were visualized using VMD.

#### Synthesis of **2**

**1** (1.0400 g, 4.9275 mmol) was added to a Schlenk flask and cycled onto a nitrogen Schlenk line. 30 mL dry THF was added to the reaction flask before cooling the solution to −78 °C. 2.5 M ^n^BuLi (2.4 mL, 6.0 mmol) was added to the solution dropwise using a syringe. The reaction mixture was allowed to stir at −78 °C for 2 h. In the meantime, Ph_2_Te_2_ (2.3296 g, 5.6900 mmol) was added to a separate Schlenk flask and cycled onto the line, and the Ph_2_Te_2_ was dissolved in 20 mL dry THF. The Ph_2_Te_2_ solution was transferred via a cannula to the flask containing **1** and ^n^BuLi still at −78 °C. The Ph_2_Te_2_ flask was rinsed and transferred with two aliquots of dry THF (15 mL followed by 10 mL). After 1 h, the reaction mixture was allowed to warm to room temperature and was left to stir overnight. The solvent was removed *in vacuo*. Then, the residue was redissolved in 200 mL benchtop DCM and filtered through a pad of Celite. 10 g silica were added to the solution before removing the solvent *in vacuo*. The resulting powder was added to a CombiFlash cartridge to perform flash chromatography through a column with 25 g silica. The eluent was monitored via the incorporated UV–vis spectrometer as it left the column to determine when the compound was eluting. The reaction mixture was eluted with 100% hexanes until the eluent showed no sign of the compound. Then, the eluent was changed to 20% EtOAc in hexanes, eluting **2** together with an impurity as one fraction. The solvent was removed from this fraction *in vacuo*. The resulting oil was redissolved in 100 mL DCM to which 5 g silica were added before removing the solvent *in vacuo*. This powder was added to a CombiFlash cartridge to perform another round of flash chromatography through a column with 65 g silica using an eluent of 5% EtOAc in hexanes, effectively isolating **2**. The solvent from the corresponding fraction was removed *in vacuo*, and the resulting oil was triturated with 3 mL pentane to promote the removal of trapped solvent. **2** was obtained as an amber oil (0.5290 g, 1.575 mmol, 32% yield). ^**1**^**H NMR** (500.13 MHz, CDCl_3_, 305.0 K) δ (ppm) 7.86 (s, 1H, indazole-N=C*H*), 7.67 (dd, *J* = 6.8, 1.0 Hz, 2H, *o*-Ph–C*H*), 7.53 (dd, *J* = 7.0, 0.4 Hz, 1H, indazole–C*H*), 7.37 (d, *J* = 8.4 Hz, 1H, indazole–C*H*), 7.26–7.21 (m, 2H, *p*-Ph–C*H* and indazole–C*H*), 7.17 (t, *J* = 14.9 Hz, 2H, *m*-Ph–C*H*), 4.06 (s, 3H, N–C*H*_3_). ^**13**^**C{**^**1**^**H} NMR** (125.77 MHz, CDCl_3_, 305.0 K) δ (ppm) 139.35 (s, N-*ipso*-indazole-*C*), 137.87 (s, *o*-Ph-*C*H), 135.85 (s, indazole-N=*C*H), 131.78 (s, indazole-*C*H), 129.66 (s, *m*-Ph-*C*H), 129.62 (s, *ipso*-Ph-*C*H), 127.98 (s, *p*-Ph-*C*H), 126.92 (s, indazole-*C*H), 114.20 (s, Te-*ipso*-indazole-*C*), 109.49 (s, indazole-*C*H), 107.19 (s, indazole-*C*), 35.95 (s, N-*C*H_3_). ^**125**^**Te{**^**1**^**H} NMR** (126.23 MHz, CDCl_3_, 298.0 K) δ (ppm) 600.3 (s). **Elemental Analysis:** Calculated for C_14_H_12_N_2_Te: C 50.07, H 3.60, N 8.34. Found: C 49.78, H 3.48, N 8.34.

#### Synthesis of [**3**]I

**2** (0.5234 g, 1.558 mmol) was transferred to a Schlenk tube with a Teflon tap using 1 mL benchtop DCM. **2** was dried *in vacuo* for 2 h before finishing the cycling of the Schlenk tube onto the line. 10 mL dry MeCN were added to the Schlenk tube followed by MeI (2.5 mL, 40. mmol). The Schlenk tube was sealed by closing the Teflon tap. The reaction solution was then refluxed for 3 days. While still at reflux, the Teflon tap was opened to vacuum, allowing for the removal of MeCN and MeI. The remaining solid was transferred to an Erlenmeyer flask using 30 mL benchtop MeCN. While stirring, solid [**3**]I and some oil **2** were precipitated from the solution using 60 mL Et_2_O. The supernatant was decanted. The mixture was triturated with 2 × 10 mL MeCN:Et_2_O (1:1 v/v), and the supernatant was decanted. Using 2 × 10 mL MeCN:Et_2_O (1:1 v/v), the resulting powder was transferred to a 20 mL scintillation vial, decanting the supernatant between aliquots. The powder was then triturated with 3 × 2 mL MeCN:Et_2_O (2:1 v/v) followed by 2 × 6 mL Et_2_O, decanting the supernatant between aliquots. The solvent was removed under a flow of compressed air before further drying the powder *in vacuo*. [**3**]I was obtained as a pale-yellow powder (0.3396 g, 0.7108 mmol, 46% yield). ^**1**^**H NMR** (500.13 MHz, CD_3_CN, 305.0 K) δ (ppm) 8.71 (s, 1H, indazole-N=C*H*), 7.84 (dd, *J* = 6.8, 0.7 Hz, 1H, indazole–C*H*), 7.78 (dd, *J* = 7.1, 1.0 Hz, 2H, *o*-Ph–C*H*), 7.72 (d, *J* = 8.9 Hz, 1H, indazole–C*H*), 7.66 (t, *J* = 15.6 Hz, 1H, indazole–C*H*), 7.36 (tt, *J* = 14.9, 2.4 Hz, 1H, *p*-Ph–C*H*), 7.27 (t, *J* = 15.0 Hz, 2H, *m*-Ph–C*H*), 4.27 (s, 3H, N–C*H*_3_), 4.15 (s, 3H, N–C*H*_3_). ^**13**^**C{**^**1**^**H} NMR** (125.77 MHz, CD_3_CN, 305.0 K) δ (ppm) 141.10 (s, N-*ipso*-indazole-*C*), 139.54 (s, *o*-Ph-*C*H), 137.28 (s, indazole-N=*C*H), 136.28 (s, indazole-*C*H), 134.49 (s, indazole-*C*H), 130.91 (s, *m*-Ph-*C*H), 129.69 (s, *p*-Ph-*C*H), 125.89 (s, indazole-*C*), 114.40 (s, *ipso*-Ph-*C*), 112.08 (s, indazole-*C*H), 110.26 (s, Te-*ipso*-indazole-*C*), 39.18 (s, N-*C*H_3_), 34.72, (s, N-*C*H_3_). ^**125**^**Te{**^**1**^**H} NMR** (126.23 MHz, CD_3_CN, 298.0 K) δ (ppm) 646.5 (s). ^**1**^**H NMR** (500.13 MHz, CDCl_3_, 305.0 K) δ (ppm) 8.92 (s, 1H, indazole-N=C*H*), 7.79 (d, *J* = 8.0 Hz, 2H, *o*-Ph–C*H*), 7.72 (dd, *J* = 5.9, 1.9 Hz, 1H, indazole–C*H*), 7.59–7.55 (m, 2H, indazole–C*H*), 7.36 (t, *J* = 14.8, 1H, *p*-Ph–C*H*), 7.28 (t, 2H, *m*-Ph–C*H*), 4.77 (s, 3H, N–C*H*_3_), 4.51 (s, 3H, N–C*H*_3_). **Elemental Analysis:** Calculated for C_15_H_15_IN_2_Te: C 37.71, H 3.16, N 5.86. Found: C 37.60, H 3.11, N 5.81.

#### Synthesis of [**3**]OTf

In a nitrogen-filled glovebox, [**3**]I (0.1610 g, 0.3370 mmol) and AgOTf (0.0962 g, 0.374 mmol) were added to a vial. 10 mL dry DCM were added, and the reaction mixture was stirred for 24 h in darkness. Afterward, the reaction mixture was filtered through a plug of Celite, using 2 × 3 mL dry DCM to rinse the reaction vial. The resulting solution was concentrated to 3 mL. While stirring vigorously, dry hexanes was added, precipitating an oil first and then a solid. A pipet was used to transfer the supernatant and the powdered solid to a new vial. The supernatant was decanted as much as possible, and the powder was rinsed with 3 × 1 mL dry hexanes before being dried *in vacuo*. The product was obtained as a pale-yellow-verging-on-white powder (0.1236 g, 0.2472 mmol, 73% yield). ^**1**^**H NMR** (500.13 MHz, CDCl_3_, 305.0 K) δ (ppm) 8.60 (s, 1H, indazole-N=C*H*), 7.75 (dd, *J* = 7.9, 1.0 Hz, 2H, *o*-Ph–C*H*), 7.70 (dd, *J* = 5.1, 2.6 Hz, 1H, indazole–C*H*), 7.56–7.52 (m, 2H, indazole–C*H*), 7.33 (tt, *J* = 14.9, 2.4 Hz, 1H, *p*-Ph–C*H*), 7.25 (t, *J* = 14.5 Hz, 2H, *m*-Ph–C*H*), 4.47 (s, 3H, N–C*H*_3_), 4.29 (s, 3H, N–C*H*_3_). ^**13**^**C{**^**1**^**H} NMR** (125.77 MHz, CDCl_3_, 305.0 K) δ (ppm) 140.62 (s, N-*ipso*-indazole-*C*), 139.09 (s, *o*-Ph-*C*H), 135.76 (s, indazole-*C*H), 135.34 (s, indazole-N=*C*H), 133.91 (s, indazole-*C*H), 130.31 (s, *m*-Ph-*C*H), 129.21 (s, *p*-Ph-*C*H), 124.97 (s, indazole-*C*), 120.85 (q, *J*_C–F_ = 321.3 Hz, ^–^O_3_S-*C*F_3_), 112.88 (s, *ipso*-Ph-*C*), 110.74 (s, Te-*ipso*-indazole-*C*), 110.41 (s, indazole-*C*H), 38.80 (s, N-*C*H_3_), 34.07 (s, N-*C*H_3_). ^**19**^**F NMR** (376.42 MHz, CDCl_3_, 298.0 K) δ (ppm) −78.4 (s). ^**125**^**Te{**^**1**^**H} NMR** (126.23 MHz, CDCl_3_, 298.0 K) δ (ppm) 652.8 (s). **Elemental Analysis:** Calculated for C_16_H_15_F_3_N_2_O_3_STe: C 38.44, H 3.02, N 5.60. Found: C 37.77, H 2.90, N 5.62.

#### Synthesis of **4**

KO^t^Bu (0.1220 g, 1.087 mmol) was added to a Schlenk flask inside a nitrogen-filled glovebox. This Schlenk flask was removed from the glovebox and cycled onto a nitrogen Schlenk line before quickly adding solid [**3**]I (0.4142 g, 0.8669 mmol) and solid Se (0.2188 g, 2.771 mmol) under a flow of nitrogen. To this solid mixture was added 15 mL dry THF. The reaction mixture was heated to reflux and allowed to stir overnight, after which the stopcock was opened to vacuum to remove the solvent. The resulting solid was dissolved in 125 mL benchtop DCM and filtered through Celite. 5 g silica were added to the solution before removing the solvent *in vacuo*. The resulting powder was added to a CombiFlash cartridge to perform flash chromatography through a column with 65 g silica. The eluent was monitored via the incorporated UV–vis spectrometer as it left the column to determine when the compound was eluting. The reaction mixture was eluted with 100% hexanes to saturate the column. Then, the eluent was changed to 1:1 DCM:hexanes and flowed through the column until two orange bands were sufficiently separated. The polarity of the eluent was increased to 9:1 DCM:hexanes, eluting **4** together with minor impurities as one fraction. The solvent was removed from this fraction *in vacuo*. The resulting solid was dissolved in 12 mL DCM in a 20 mL scintillation vial which was then placed in a freezer, precipitating a solid. The supernatant was decanted into a separate 20 mL scintillation vial, and the remaining solid was triturated with 1 × 10 mL hexanes followed by 2 × 3 mL hexanes. Residual solvent was removed *in vacuo*, yielding **4** as a dandelion-yellow solid (0.1000 g, 0.2332 mmol, 27% yield). ^**1**^**H NMR** (500.13 MHz, CDCl_3_, 305.0 K) δ (ppm) 7.97 (d, *J* = 6.8 Hz, 2H, *o*-Ph–C*H*), 7.44 (t, *J* = 14.9 Hz, 1H, *p*-Ph–C*H*), 7.35 (t, *J* = 14.9 Hz, 2H, *m*-Ph–C*H*), 7.11 (t, *J* = 15.8 Hz, 1H, indazole-*H*), 6.92 (d, *J* = 8.2 Hz, 1H, indazole-*H*), 6.75 (d, *J* = 7.6 Hz, 1H, indazole-*H*), 4.00 (s, 3H, N–C*H*_3_), 3.66 (s, 3H, N–C*H*_3_). ^**13**^**C{**^**1**^**H} NMR** (125.77 MHz, CDCl_3_, 305.0 K) δ (ppm) 166.05 (d, *J*_*C–Se*_ = 11.6 Hz, *C*=Se), 144.62 (s, N-*ipso*-indazole-*C*), 141.57 (s, *m*-Ph-*C*H), 132.12 (s, indazole-*C*H), 130.54 (s, Te-*ipso*-indazole-*C*), 129.89 (s, *o*-Ph-*C*H), 128.80 (s, *p*-Ph-*C*H), 127.10 (s, indazole-*C*H), 124.99 (s, indazole-*C*), 119.76 (s, *ipso*-Ph-*C*), 106.10 (s, indazole-*C*H), 35.03 (s, N-*C*H_3_), 34.41 (N-*C*H_3_). ^**77**^**Se{**^**1**^**H} NMR** (76.33 MHz, CDCl_3_, 298.0 K) δ (ppm) 204.2 (s). ^**125**^**Te{**^**1**^**H} NMR** (126.23 MHz, CDCl_3_, 298.0 K) δ (ppm) 656.7 (s). **Elemental Analysis:** Calculated for C_15_H_14_N_2_SeTe: C 42.01, H 3.29, N 6.53. Found: C 42.16, H 3.28, N 6.49.

#### Synthesis of [**5**]OTf

In a nitrogen-filled glovebox, **4** (0.0506 g, 0.118 mmol) was weighed into a vial and dissolved in 2 mL dry DCM. MeOTf (0.0203 g, 0.124 mmol) was weighed into a small test tube and transferred to the vial containing **4** using 2 × 1 mL dry DCM. The reaction mixture was allowed to sit for 1 h before diluting the solution up to 10 mL with dry DCM and layering with 10 mL dry hexanes. The solution was allowed to sit overnight. The solvent was removed *in vacuo* before redissolving the residue in 3 mL dry DCM and layering with 17 mL dry hexanes. This layered solution was placed in the freezer, producing a precipitate. The solvent was decanted. The residue was dissolved in minimal DCM and again placed in the freezer, precipitating the product. The solvent was decanted, and the product was rinsed with 3 × 2 mL dry hexanes before being dried *in vacuo*. The product was obtained as a pale-yellow powder (0.0370 g, 0.0624 mmol, 53% yield). ^**1**^**H NMR** (500.13 MHz, CDCl_3_, 305.0 K) δ (ppm) 7.96 (dd, *J* = 7.7, 1.0, 2H, *o*-Ph–C*H*), 7.53 (t, 15.0 Hz, 1H, *p*-Ph–C*H*), 7.42 (t, *J* = 15.2 Hz, 2H, *m*-Ph–C*H*), 7.38–7.33 (m, 2H, indazole-*H*), 6.98 (dd, *J* = 6.4, 1.4 Hz, 1H, indazole-*H*), 4.65 (s, 3H, N–C*H*_3_), 4.42 (s, 3H, N–C*H*_3_), 2.64 (t, *J*_Se–H_ = 6.2 Hz, 3H, Se–C*H*_3_). ^**13**^**C{**^**1**^**H} NMR** (125.77 MHz, CDCl_3_, 305.0 K) δ (ppm) 141.79 (s, *o*-Ph-*C*H), 140.95 (s, N-*ipso*-indazole-*C*), 134.10 (s, *C*=Se), 130.63 (s, indazole-*C*H), 130.56 (s, *m*-Ph-*C*H), 130.05 (s, *p*-Ph-*C*H), 126.43 (s, Te-*ipso*-indazole-*C*), 120.71 (q, *J*_C–F_ = 319.6 Hz, ^–^O_3_S-*C*F_3_), 117.47 (indazole-*C*), 113.68 (s, *ipso*-Ph-*C*), 108.25 (s, indazole-*C*H), 37.33 (s, N-*C*H_3_), 35.43 (s, N-*C*H_3_), 14.06 (s, Se-*C*H_3_). ^**19**^**F NMR** (376.42 MHz, CDCl_3_, 298.0 K) δ (ppm) −78.5 (s). ^**77**^**Se{**^**1**^**H} NMR** (76.33 MHz, CDCl_3_, 298.0 K) δ (ppm) 90.8 (s). ^**125**^**Te{**^**1**^**H} NMR** (126.23 MHz, CDCl_3_, 298.0 K) δ (ppm) 679.8 (s). **Elemental Analysis:** Calculated for C_17_H_17_F_3_N_2_O_3_SSeTe: C 34.44, H 2.89, N 4.72. Found: C 34.31, H 2.76, N 4.56.

#### Synthesis of **7**

KO^t^Bu (0.4020 g, 3.583 mmol) was added to a Schlenk flask inside a nitrogen-filled glovebox. This Schlenk flask was removed from the glovebox and cycled onto a nitrogen Schlenk line before quickly adding solid [**6**]I (0.7995 g, 2.917 mmol) and solid Se (0.6981 g, 8.840 mmol) under a flow of nitrogen. To this solid mixture was added 30 mL dry THF. The reaction mixture was heated to reflux and allowed to stir overnight. The solvent was removed *in vacuo*. The resulting solid was dissolved in 125 mL benchtop DCM and filtered through Celite, after which the DCM was removed *in vacuo*. The solid was triturated with 3 × 3 mL Et_2_O, and the supernatant was decanted. The residue was again taken up in DCM and filtered through a silica pipet column. The column was eluted with DCM until the eluent flowed colorless. The solvent was removed under vacuum, yielding **7** as a light-orange solid (0.2208 g, 0.9806 mmol, 34% yield). ^**1**^**H NMR** (500.13 MHz, CDCl_3_, 305.0 K) δ (ppm) 8.10 (d, *J* = 7.7 Hz, 1H, indazole-*H*), 7.61 (t, *J* = 15.47 Hz, 1H, indazole-*H*), 7.27–7.23 (m, 2H, indazole-*H*), 4.13 (s, 3H, N–C*H*_3_), 3.73 (s, 3H, N–C*H*_3_). ^**13**^**C{**^**1**^**H} NMR** (125.77 MHz, CDCl_3_, 305.0 K) δ (ppm) 165.46 (s, *C*=Se), 143.36 (s, N-*ipso*-indazole-*C*), 132.44 (s, indazole-*C*H), 129.63 (s, indazole-*C*), 126.99 (s, indazole-*C*H), 123.09 (s, indazole-*C*H), 109.43 (s, indazole-*C*H), 34.98 (s, N-*C*H_3_), 34.57 (s, N-*C*H_3_). ^**77**^**Se{**^**1**^**H} NMR** (76.33 MHz, CDCl_3_, 298.0 K) δ (ppm) 131.0 (s). **Elemental Analysis:** Calculated for C_9_H_10_N_2_Se: C 48.01, H 4.48, N 12.44. Found: C 48.26, H 4.51, N 12.50.

#### Synthesis of [**8**]OTf

In a glovebox, **7** (0.0390 g, 0.173 mmol) was weighed into a vial and dissolved in 2 mL dry DCM. MeOTf (0.0602 g, 0.367 mmol) was weighed into a small test tube and transferred to the vial containing **7** using 3 × 0.5 mL dry DCM. The solution was swirled and allowed to sit overnight. The product was precipitated using dry hexanes, and the solvent was decanted. The precipitate was triturated with 2 × 1 mL dry hexanes before being dried *in vacuo*. The product was obtained as a tan powder (0.0557 g, 0.143 mmol, 83% yield). ^**1**^**H NMR** (500.13 MHz, CDCl_3_, 305.0 K) δ (ppm) 7.93 (d, *J* = 8.6 Hz, 1H, indazole-*H*), 7.83 (t, *J* = 15.7 Hz, 1H, indazole-*H*), 7.67 (d, *J* = 8.9 Hz, 1H, indazole-*H*), 7.51 (t, *J* = 15.4 Hz, 1H, indazole-*H*), 4.60 (s, 3H, N–C*H*_3_), 4.43 (s, 3H, N–C*H*_3_), 2.57 (t, *J*_Se–H_ = 6.2 Hz, 3H, Se–C*H*_3_). ^**13**^**C{**^**1**^**H} NMR** (125.77 MHz, CDCl_3_, 305.0 K) δ (ppm) 140.19 (s, indazole-*C*), 134.73 (s, *C*=Se), 134.21 (s, indazole-*C*H), 126.02 (s, indazole-*C*H), 124.54 (s, N-*ipso*-indazole-*C*), 123.09 (s, indazole-*C*H), 120.69 (q, *J*_C–F_ = 320.5 Hz, ^–^O_3_S-*C*F_3_), 111.14 (s, indazole-*C*H), 37.41 (s, N-*C*H_3_), 35.19 (s, N-*C*H_3_), 11.00 (s, Se-*C*H_3_). ^**19**^**F NMR** (376.42 MHz, CDCl_3_, 298.0 K) δ (ppm) −78.6 (s). ^**77**^**Se{**^**1**^**H} NMR** (76.33 MHz, CDCl_3_, 298.0 K) δ (ppm) 103.3 (s). **Elemental Analysis:** Calculated for C_11_H_13_F_3_N_2_O_3_SSe: C 33.94, H 3.37, N 7.20. Found: C 33.68, H 3.21, N 7.13.

### Catalysis

All manipulations were conducted in a nitrogen-filled glovebox. Commercial CDCl_3_ was dried over 4 Å molecular sieves to remove water. Quinoline and mesitylene were freeze–pump–thawed before being brought into the glovebox and dried over 4 Å molecular sieves. A stock solution was prepared by adding 123 μL of quinoline (1.04 mmol) and 50.0 μL of mesitylene (0.359 mmol) together and diluting up to 10.00 mL with CDCl_3_ in a volumetric flask. In a typical experiment, the catalyst (10 mol%) was weighed into a 20 mL scintillation vial before adding 1.00 mL of the stock solution. The vial was vigorously shaken until the solution was transparent. Due to the poor solubility of [**6**]OTf, the vial was shaken until as much of the weighed-out 10 mol% dissolved as possible. Hantzsch ester (40.0 mg, 0.158 mmol) was weighed directly into a J. Young tube. 0.70 mL of the solution containing the catalyst, quinoline, and mesitylene were added to the J. Young tube containing Hantzsch ester. The J. Young tube was sealed, transferred outside the glovebox, and laid horizontally on an Onilab SK-O180-Pro shaker, which shook the tube at 670 rpm for 20 h. After 20 h, the progress of the reaction was determined using ^1^H NMR spectroscopy. The conversions were calculated by comparing the integral of a pair of doublets for the aromatic protons of the starting material (8.08 and 8.03 ppm, 2H) to the quintet for the product (1.85 ppm, 2H). The spectra for each reaction after 20 h are given in Figures S37–S43.

## Supporting Information Available

NMR spectra for the synthesized compound, NMR spectra for catalytic studies, and computational details (PDF); The Supporting Information is available free of charge at https://pubs.acs.org/doi/10.1021/acs.organomet.4c00094.

NMR spectra for the synthesized compound, NMR spectra for catalytic studies, and computational details (PDF)
Optimized geometries **2** (XYZ)
**3^+^** (XYZ)
**4** (XYZ)
**5^+^** (XYZ)
**6^+^**(XYZ)
**7** (XYZ)
**8^+^** (XYZ)
**D** (XYZ)
**E** (XYZ)

## Supplementary Material

om4c00094_si_001.pdf

om4c00094_si_003.xyz

om4c00094_si_004.xyz

om4c00094_si_005.xyz

om4c00094_si_006.xyz

om4c00094_si_007.xyz

om4c00094_si_008.xyz

om4c00094_si_009.xyz

om4c00094_si_010.xyz

om4c00094_si_011.xyz
