# Supplementary material for: Exploring the Effects of Se Basicity on a Te···Se Interaction Supported by a Rigid Indazolium Backbone
Source: Organometallics. 2024 May 29;43(11):1246–55. doi: 10.1021/acs.organomet.4c00094 (PMC11167644; doi:10.1021/acs.organomet.4c00094)
Supplement: Supplementary file 1 — om4c00094_si_001.pdf [file om4c00094_si_001.pdf]

Exploring the Effects of Se Basicity on a Te···Se Interaction Supported by a Rigid Indazolium Backbone

Logan T. Maltz and François P. Gabbaï*

Department of Chemistry, Texas A&M University, College Station, Texas 77843-3255, United States

*Email: francois@tamu.edu

SUPPORTING INFORMATION

This PDF file includes

Contents

NMR Spectra for Synthesized Compounds 2

NMR spectra for compound **2** 2

NMR spectra for compound [**3**]I 6

NMR spectra for compound [**3**]OTf 11

NMR spectra for compound **4** 16

NMR spectra for compound [**5**]OTf 22

NMR spectra for compound **7** 29

NMR spectra for compound [**8**]OTf 33

NMR Spectra for Catalytic Studies 38

Computational Details 45

Optimized geometries 47

NBO interactions in **D** and **E** 48

NMR Spectra for Synthesized Compounds

NMR spectra for compound 2

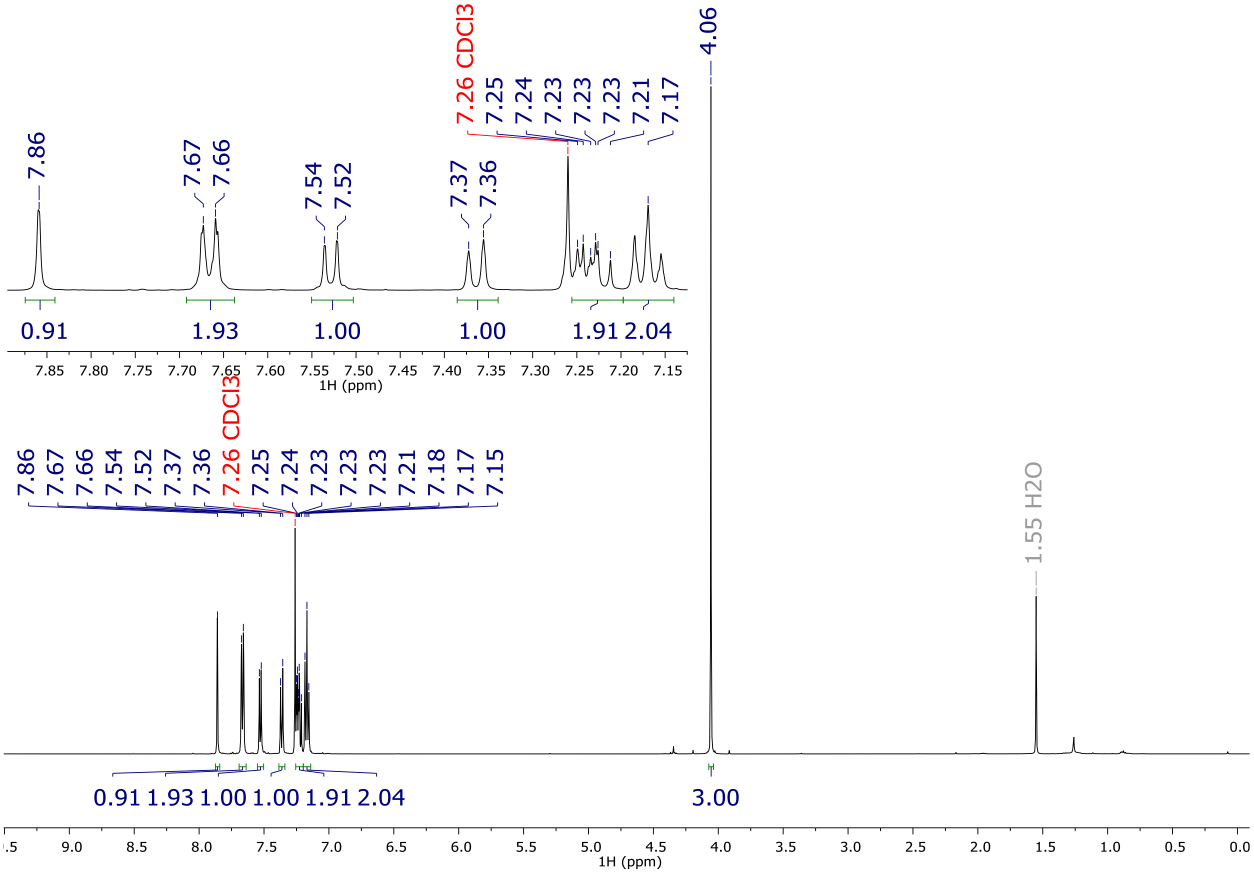

Figure S1. ^{1}H NMR spectrum of **2** in $CDCl_3$.

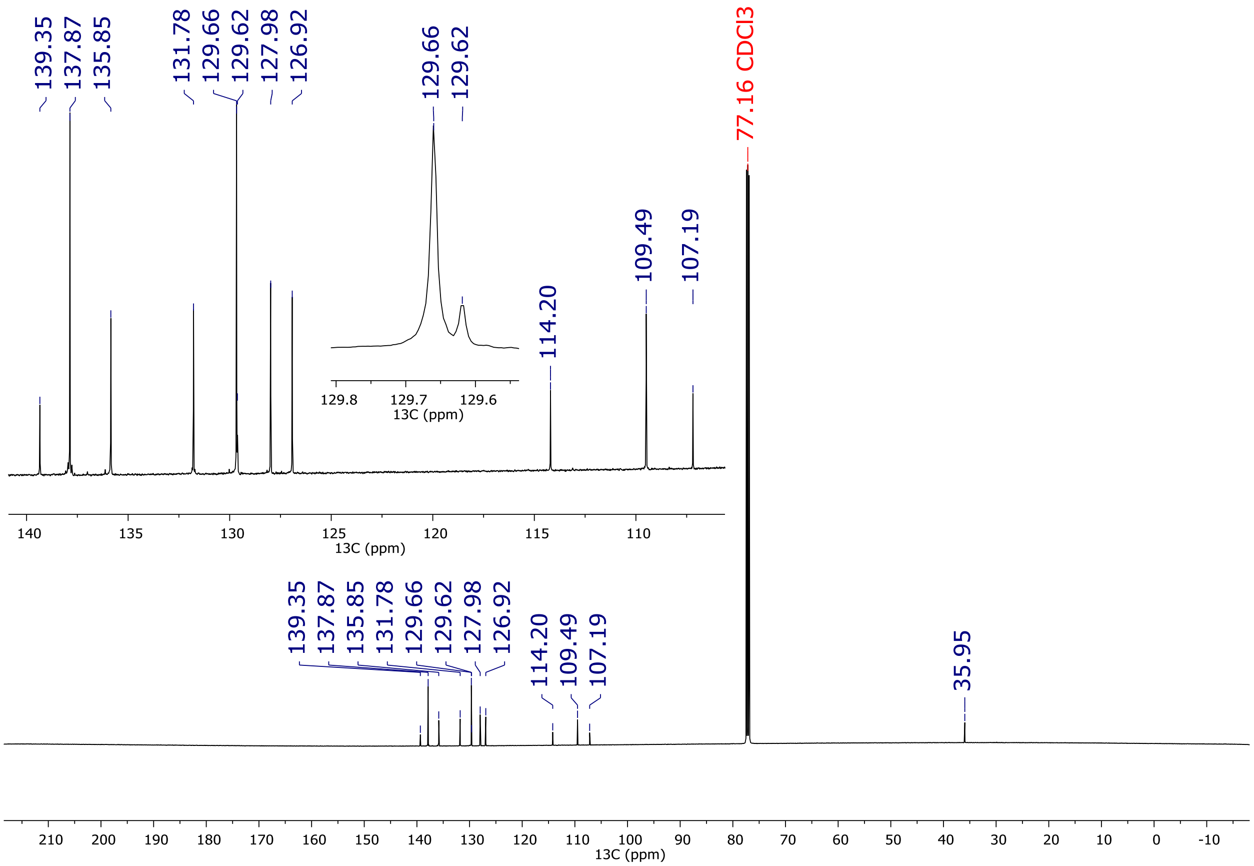

Figure S2. $^{13}C\{^{1}H\}$ NMR spectrum of **2** in $CDCl_3$.

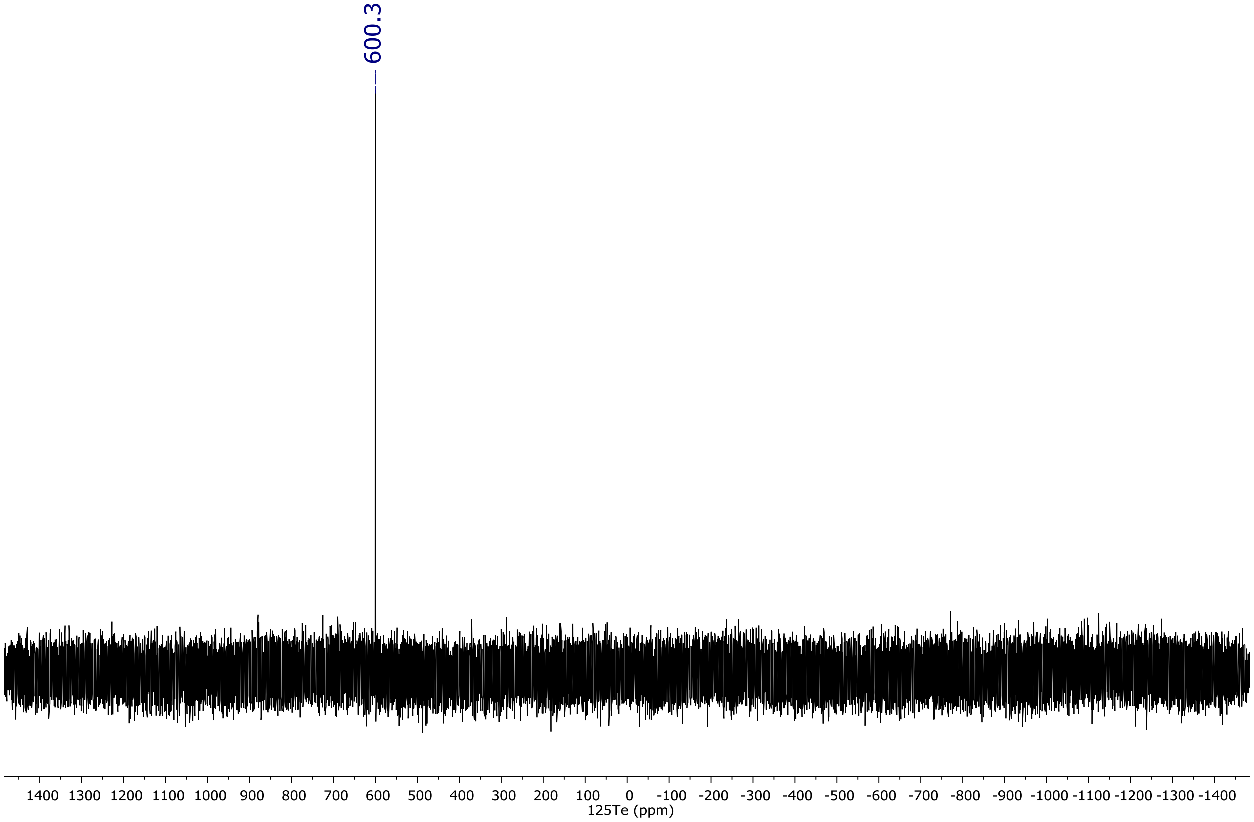

Figure S3. ^{125}Te{^{1}H} NMR spectrum of **2** in $CDCl_3$.

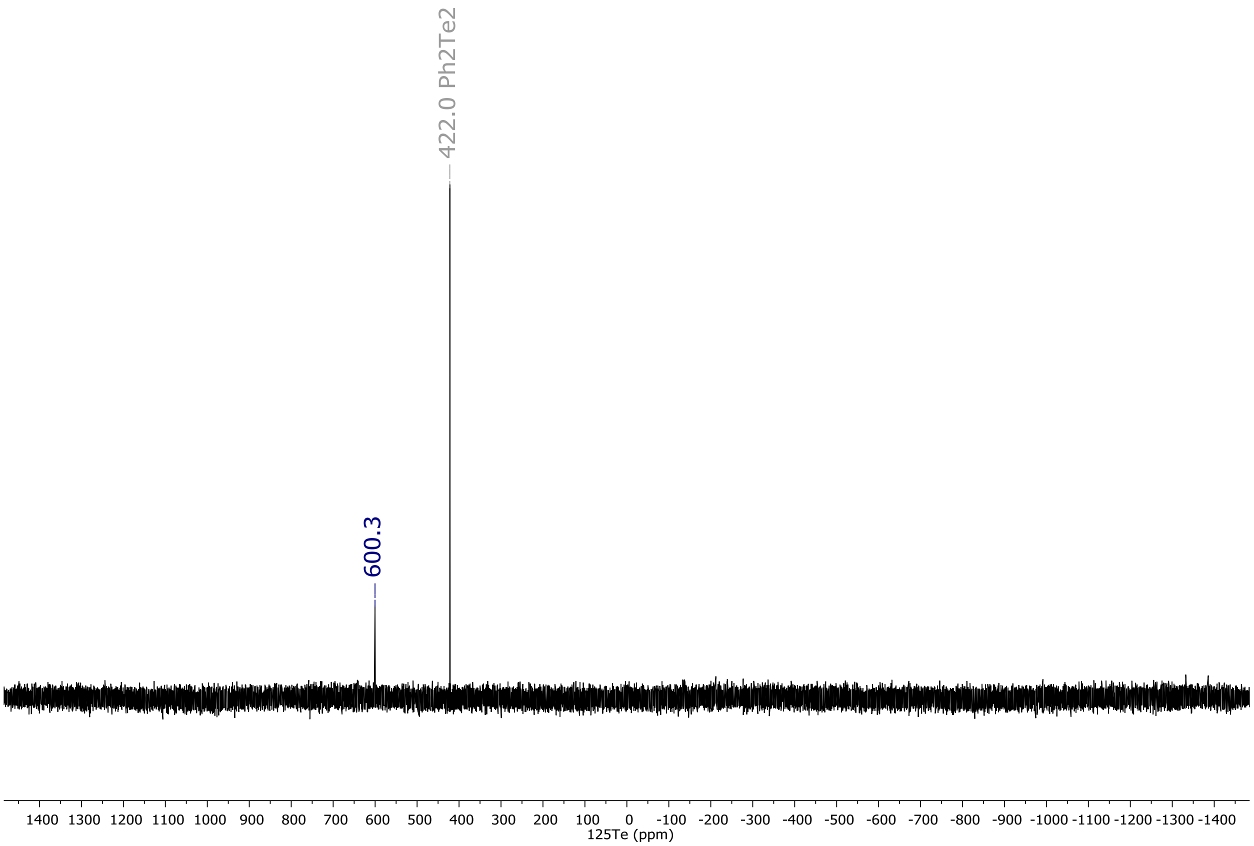

Figure S4. $^{125}Te\{^1H\}$ NMR spectrum of **2** in $CDCl_3$ with Ph_2Te_2 capillary.

NMR spectra for compound [3]I

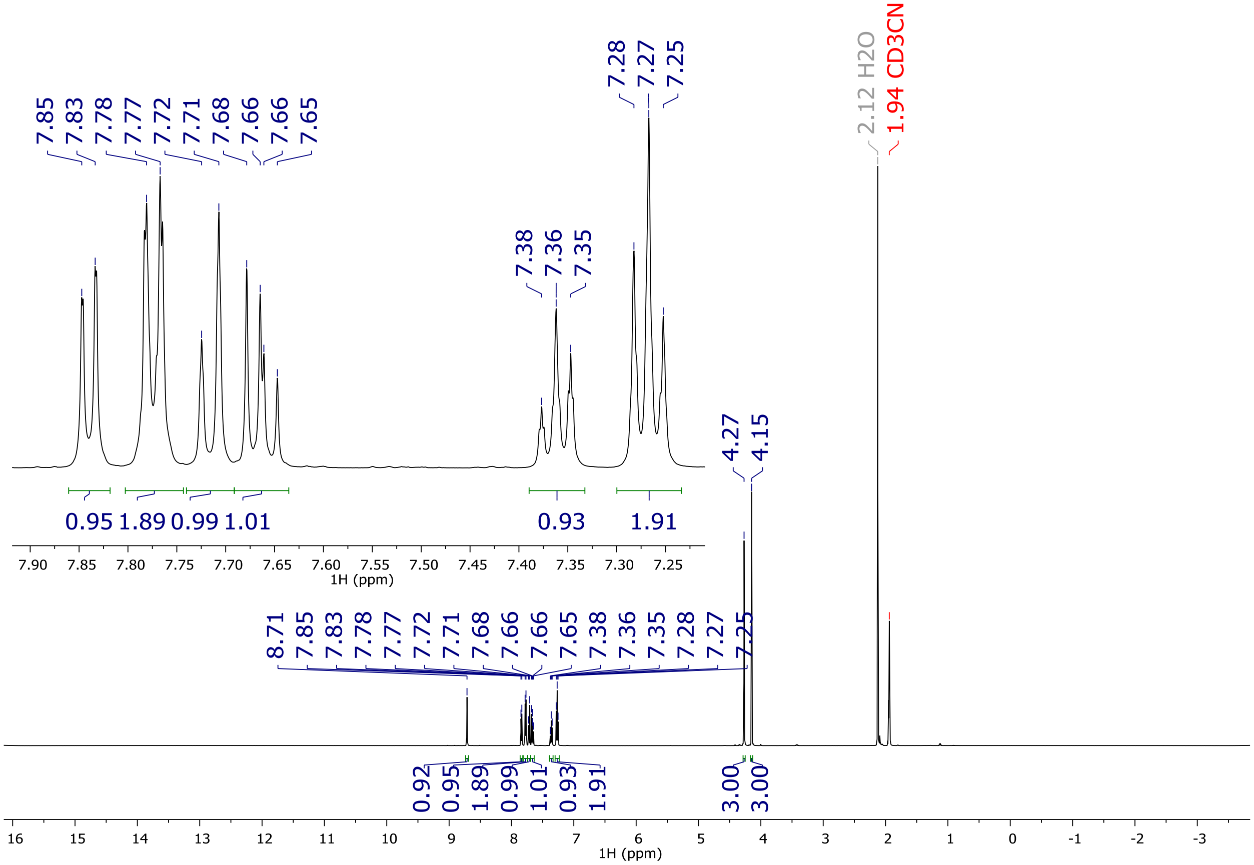

Figure S5. ${}^{1}H$ NMR spectrum of [**3**]I in CD_3CN.

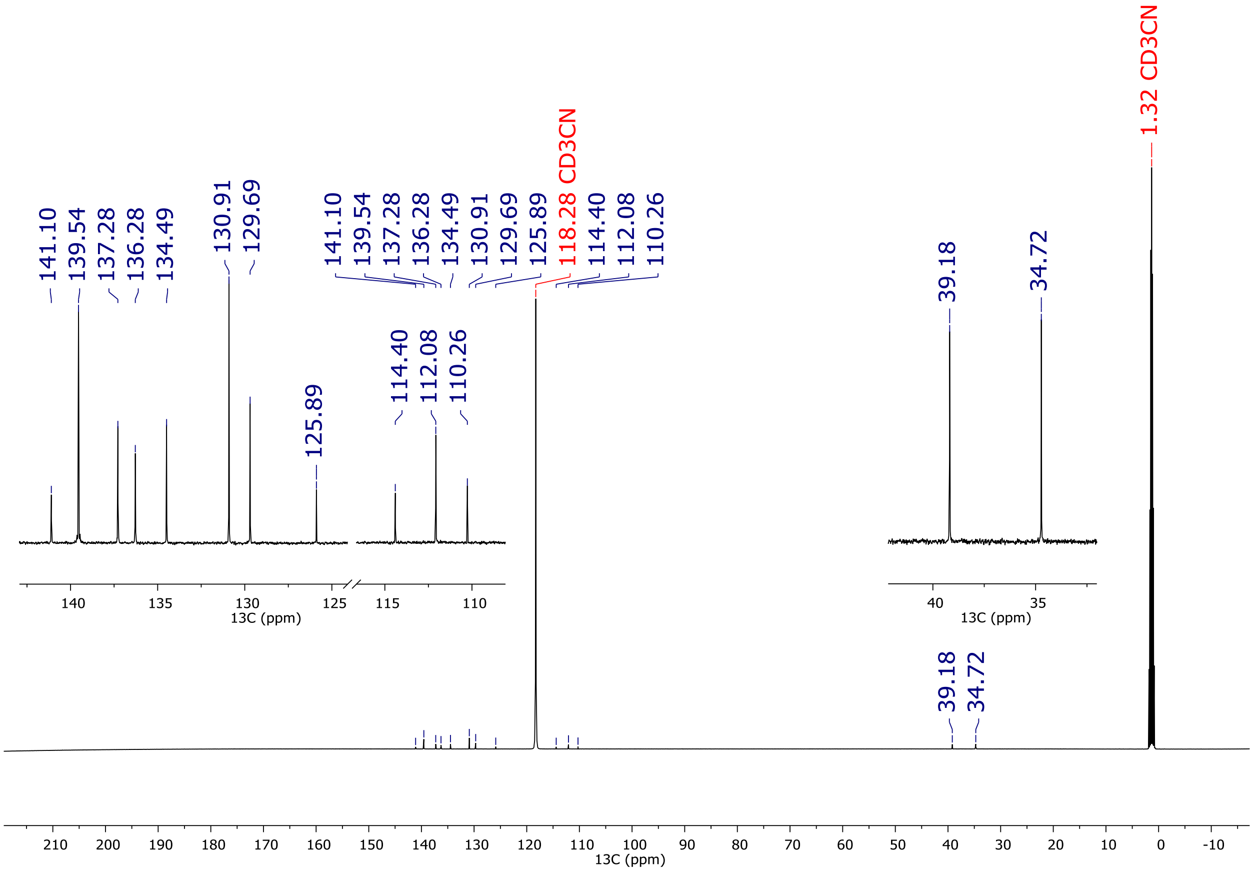

Figure S6. $^{13}C\{^1H\}$ NMR spectrum of [**3**]I in CD_3CN.

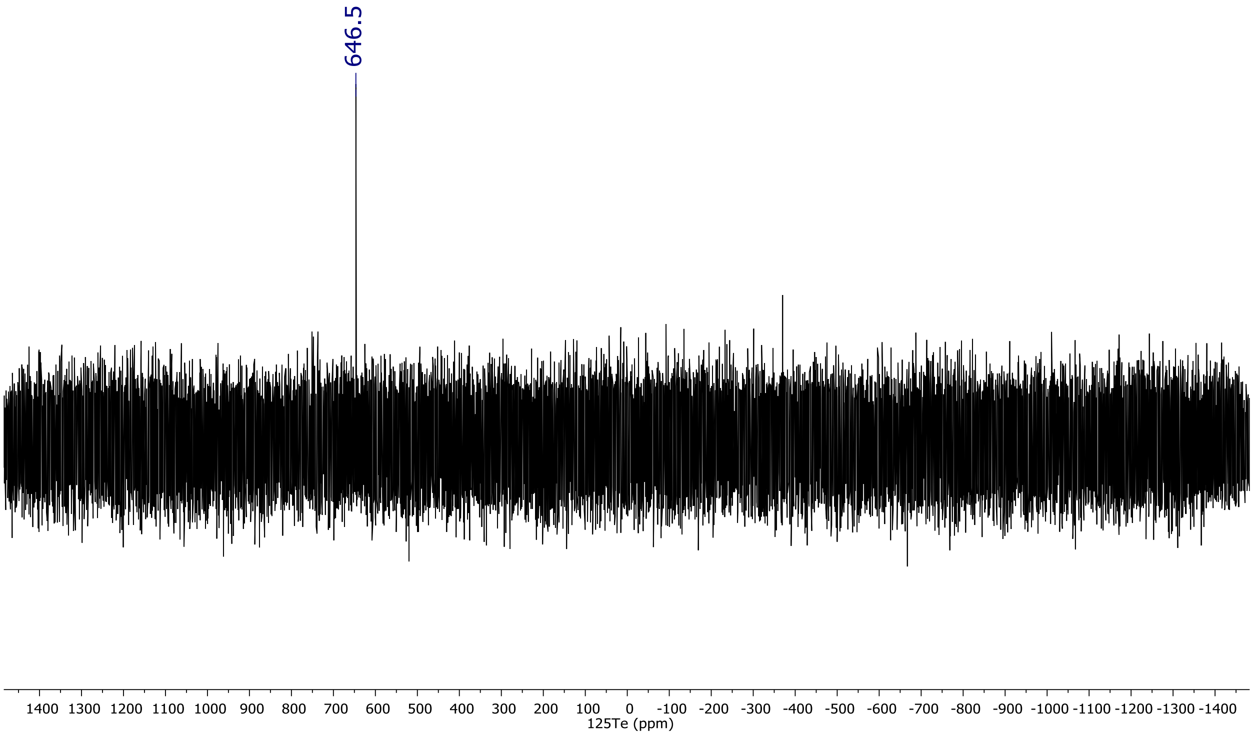

Figure S7. $^{125}Te\{^1H\}$ NMR spectrum of [**3**]I in CD_3CN.

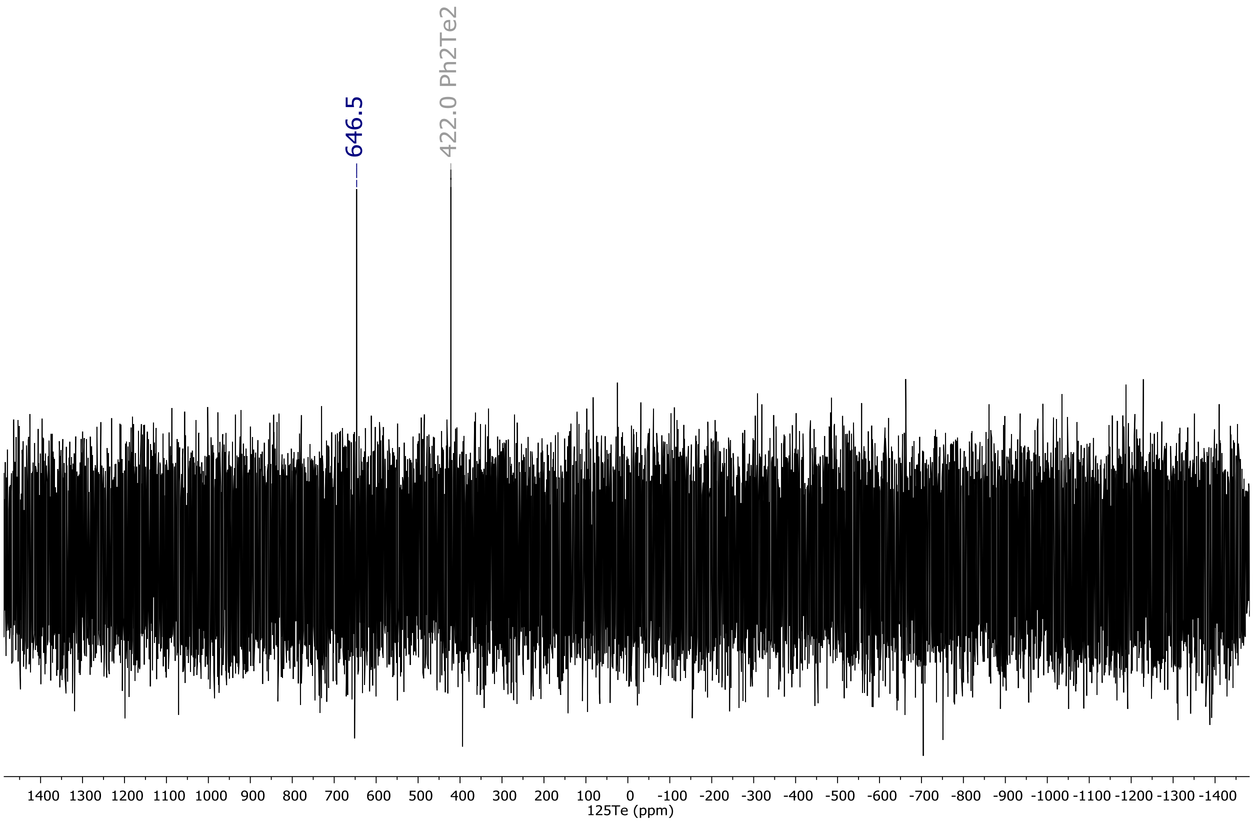

Figure S8. $^{125}Te\{^1H\}$ NMR spectrum of [**3**]I in CD_3CN with Ph_2Te_2 capillary.

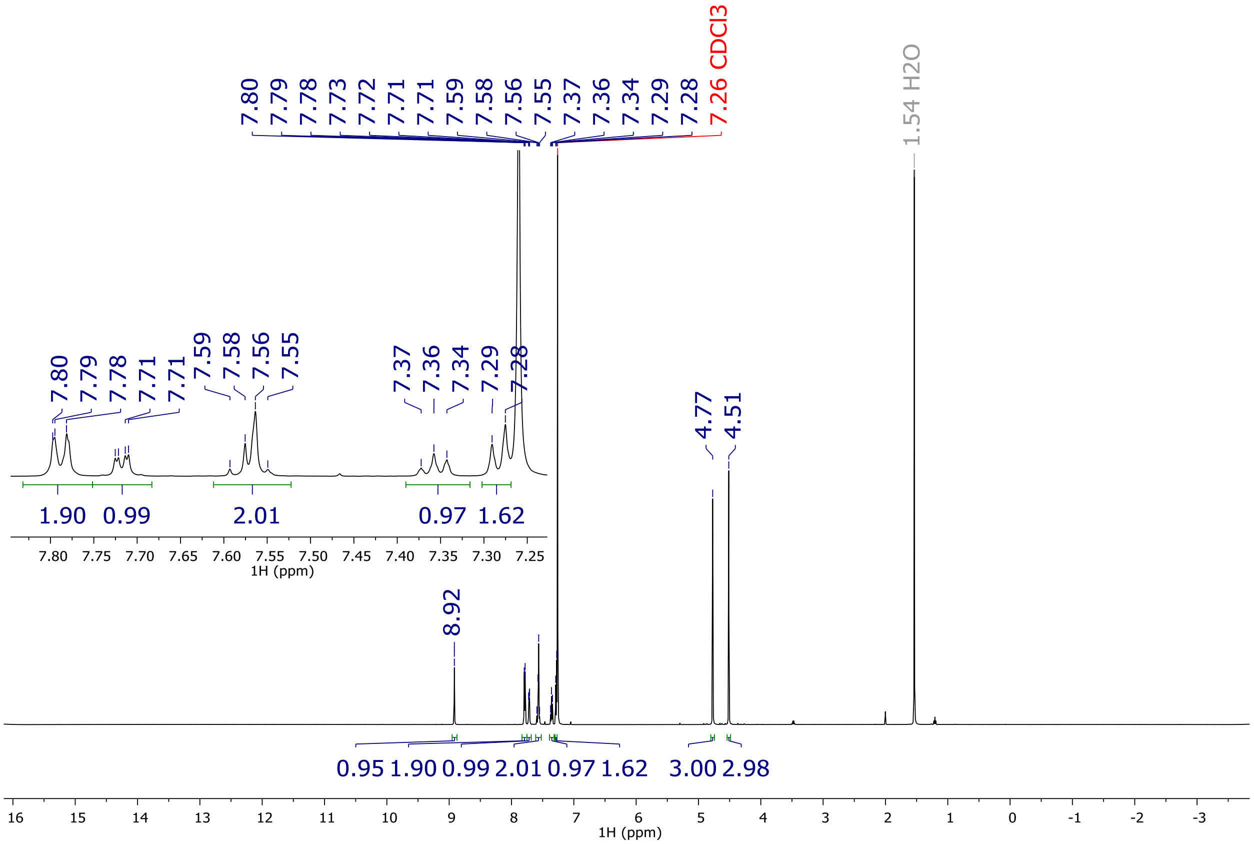

Figure S9. ^{1}H NMR spectrum of [**3**]I in $CDCl_3$.

NMR spectra for compound [3]OTf

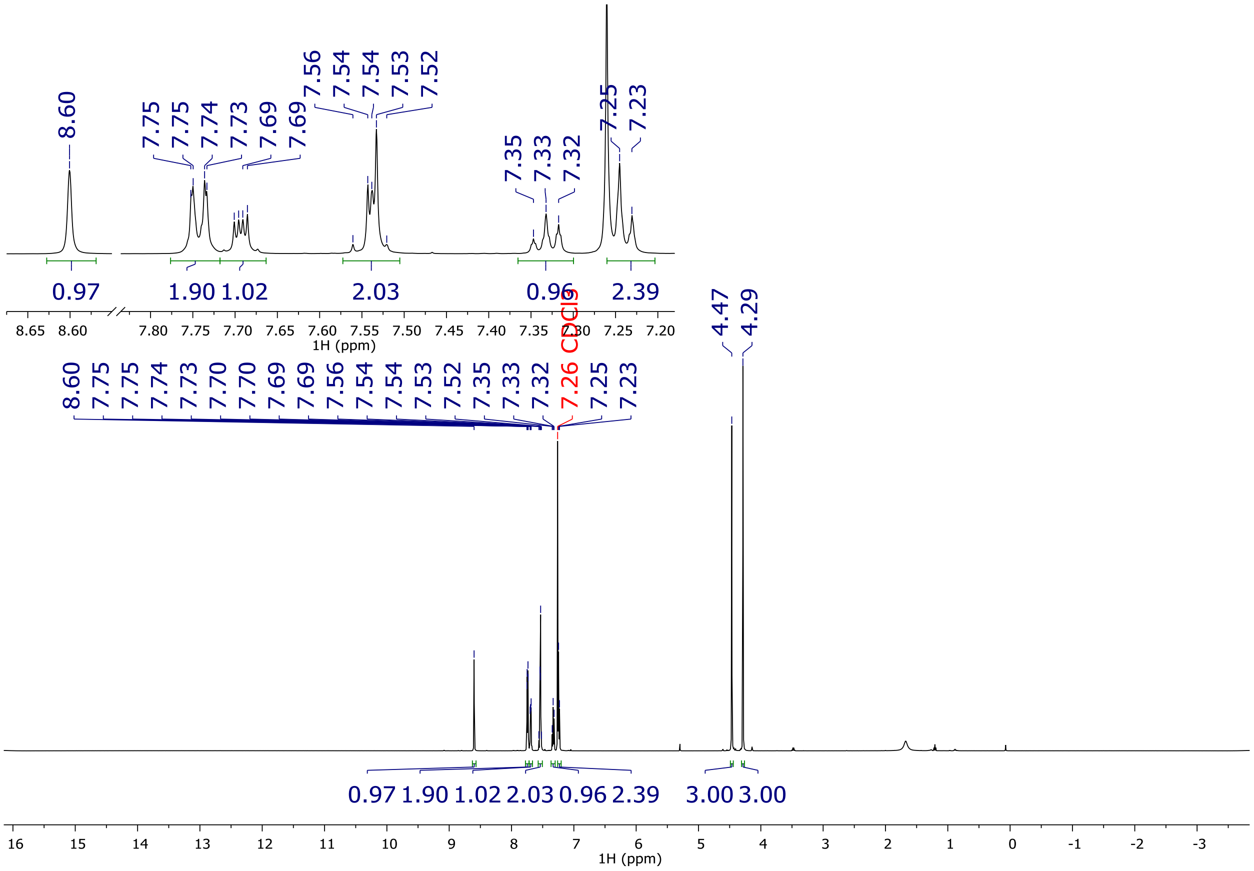

Figure S10. ^{1}H NMR spectrum of [**3**]OTf in $CDCl_3$.

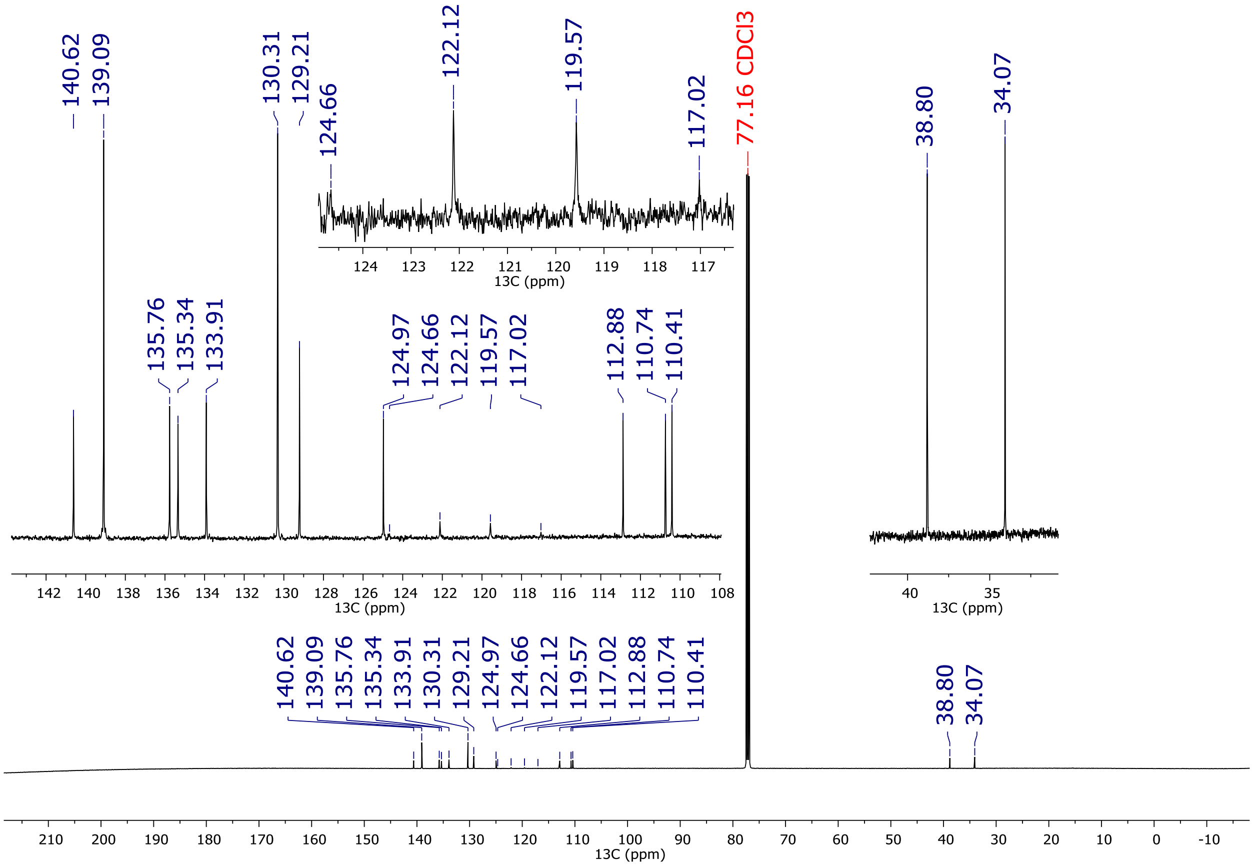

Figure S11. $^{13}C\{^{1}H\}$ NMR spectrum of [**3**]OTf in $CDCl_3$.

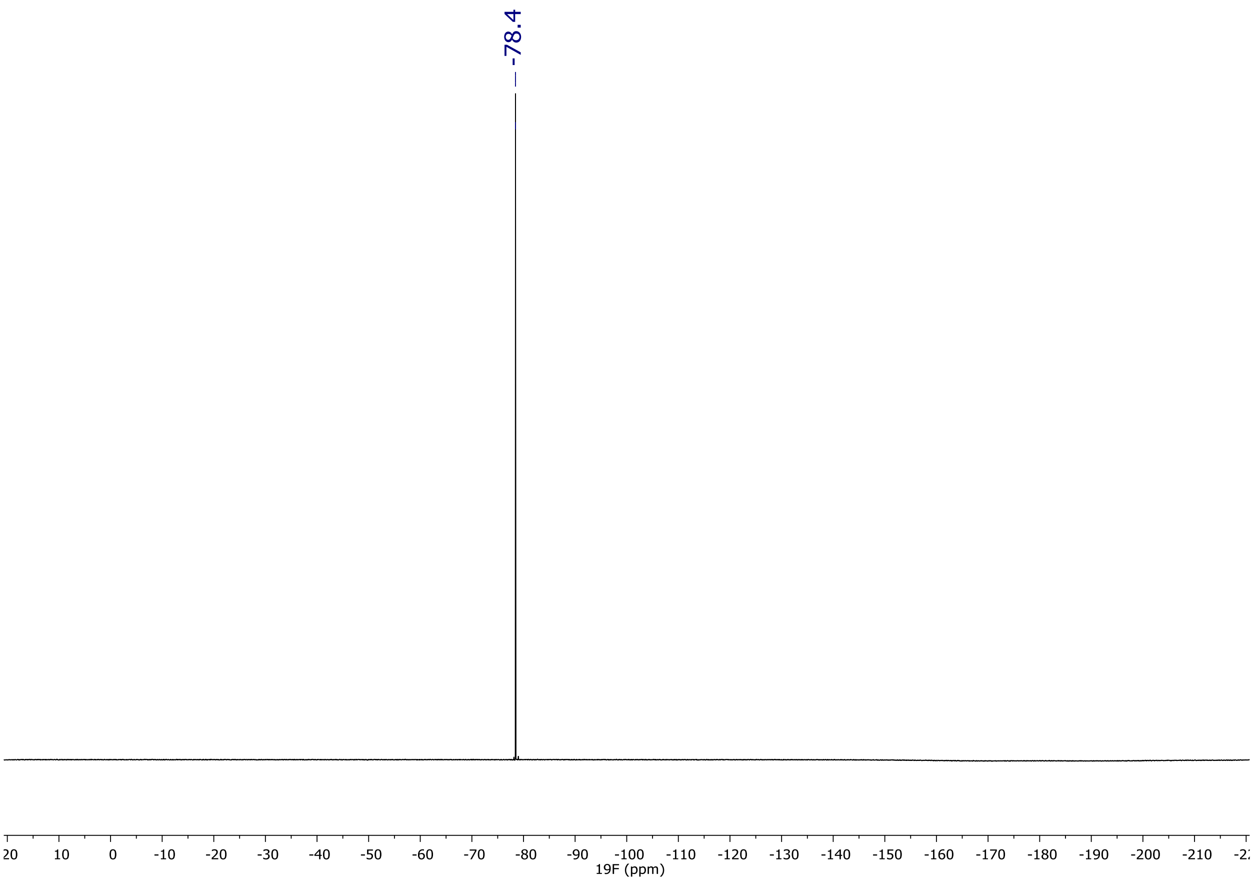

Figure S12. ^{19}F NMR spectrum of [**3**]OTf in $CDCl_3$.

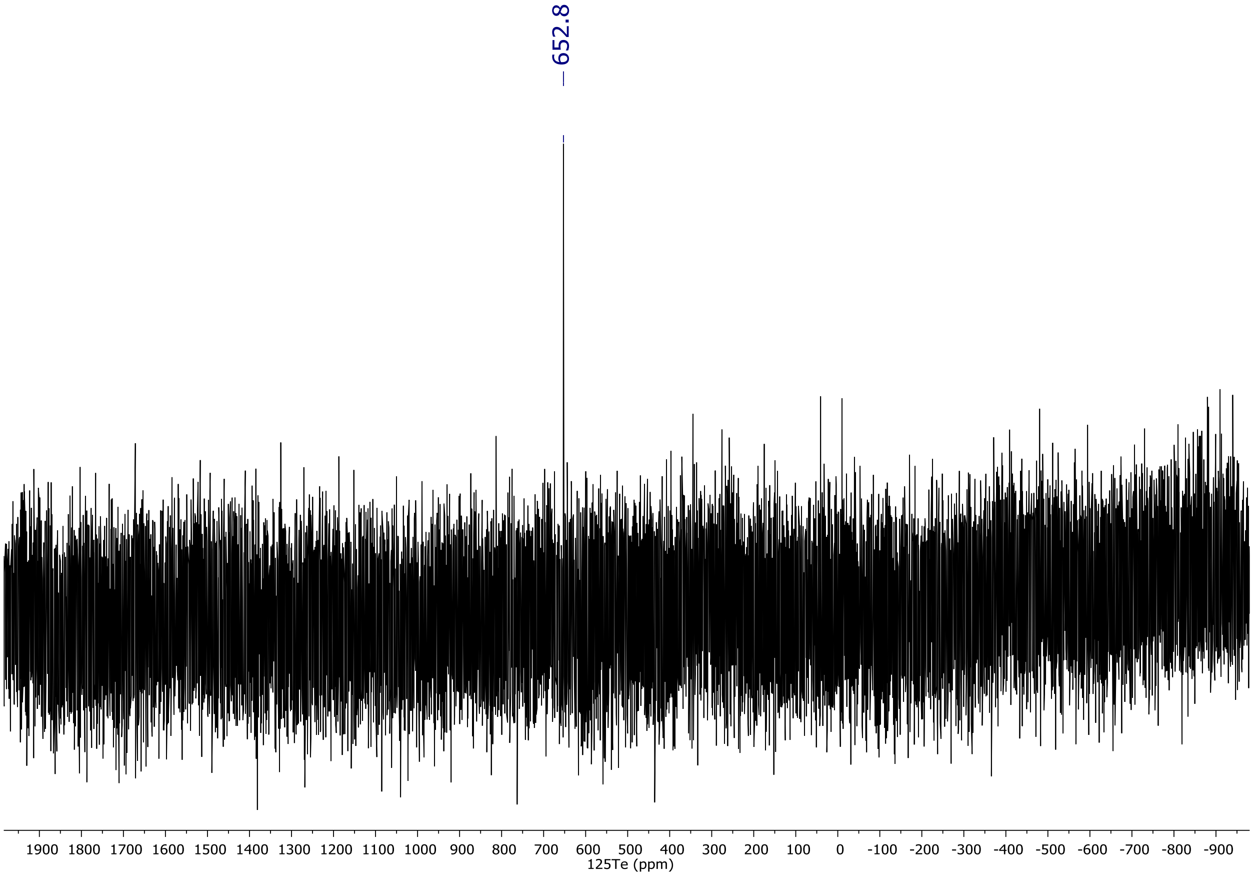

Figure S13. $^{125}Te\{^{1}H\}$ NMR spectrum of [**3**]OTf in $CDCl_3$.

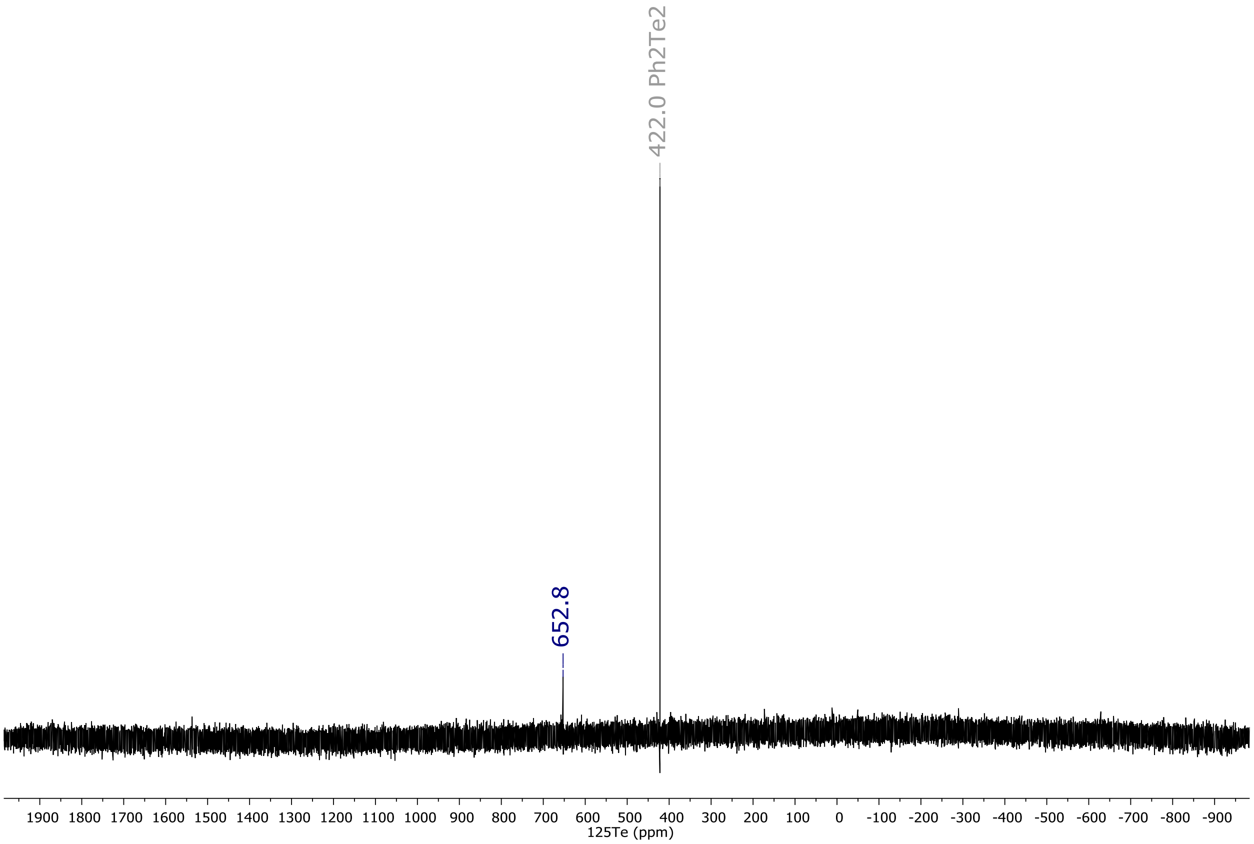

Figure S14. $^{125}Te\{^{1}H\}$ NMR spectrum of [**3**]OTf in $CDCl_3$ with Ph_2Te_2 capillary.

NMR spectra for compound 4

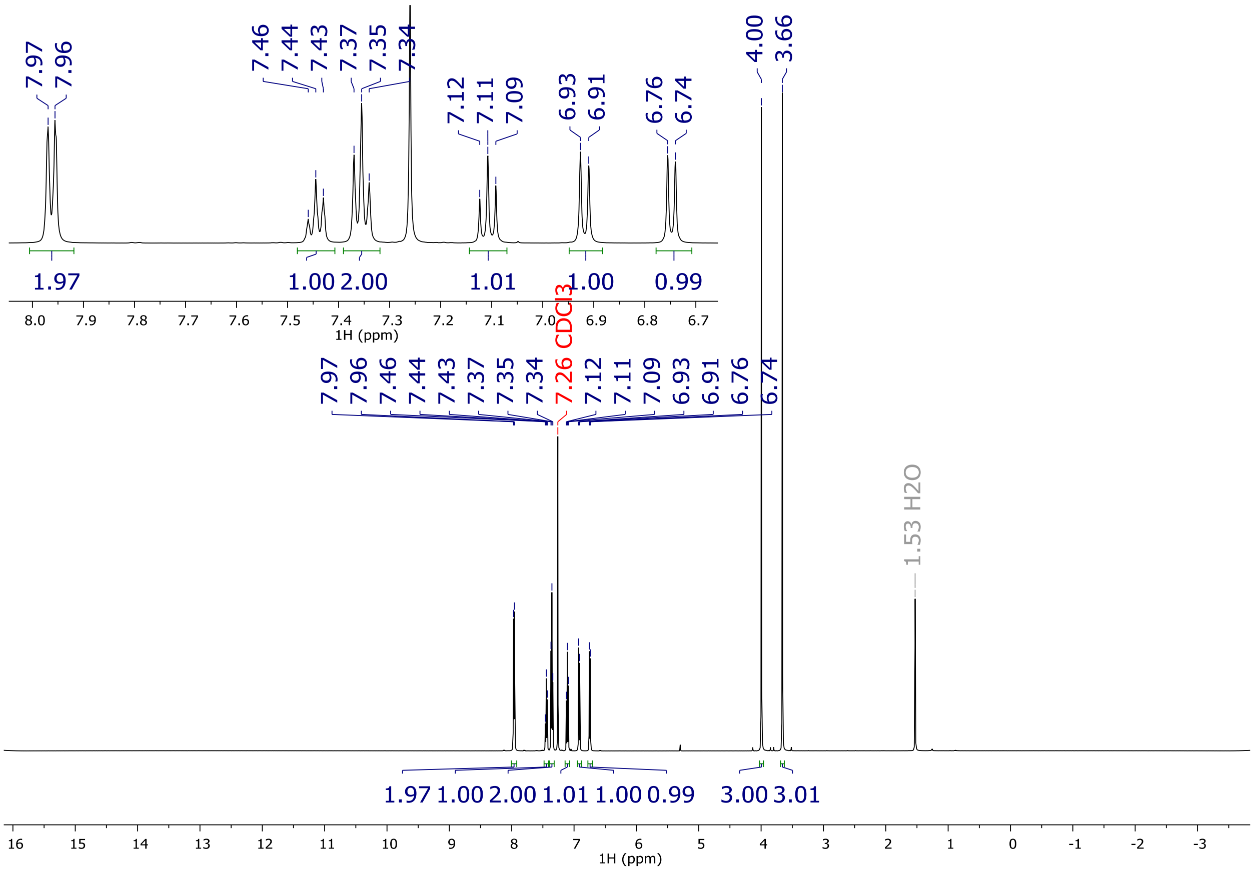

Figure S15. ^{1}H NMR spectrum of **4** in $CDCl_3$.

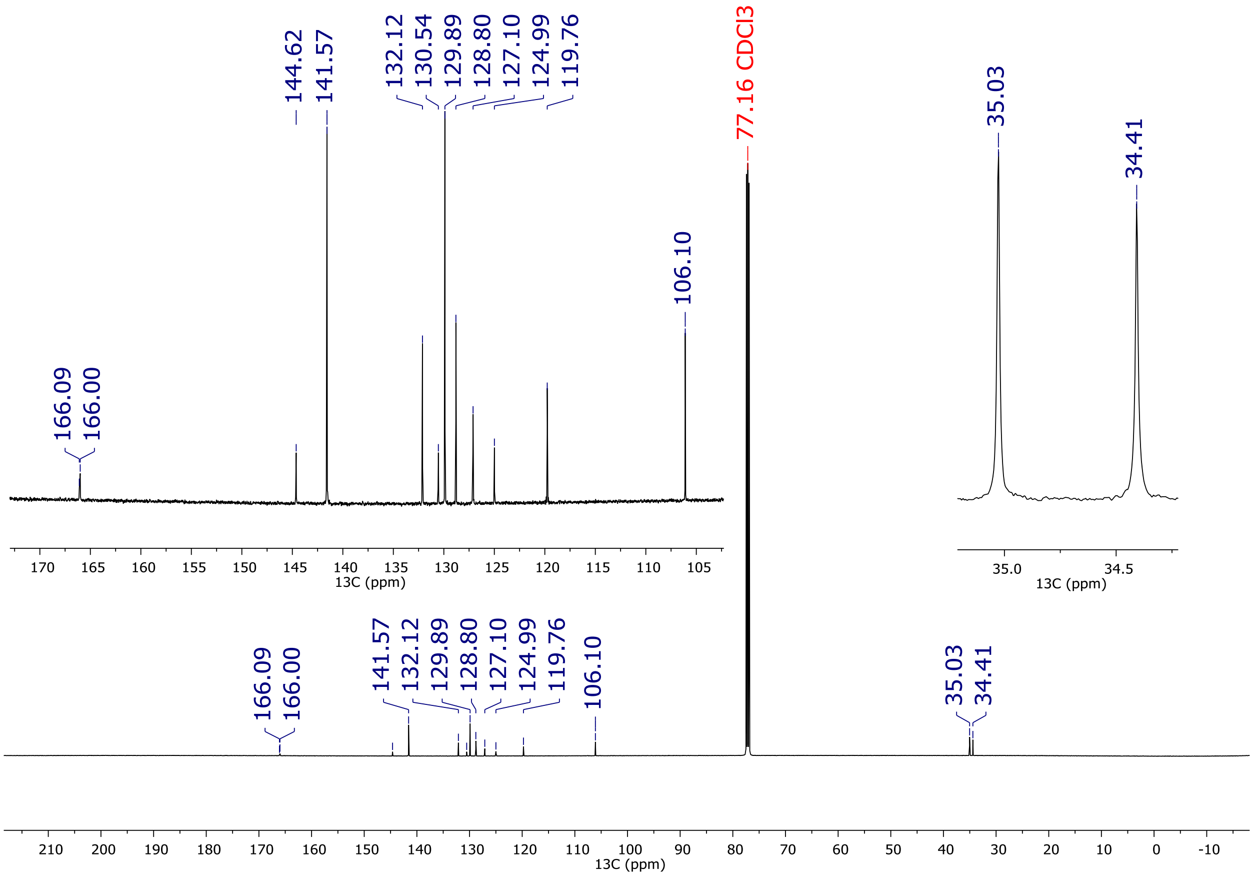

Figure S16. $^{13}C\{^{1}H\}$ NMR spectrum of **4** in $CDCl_3$.

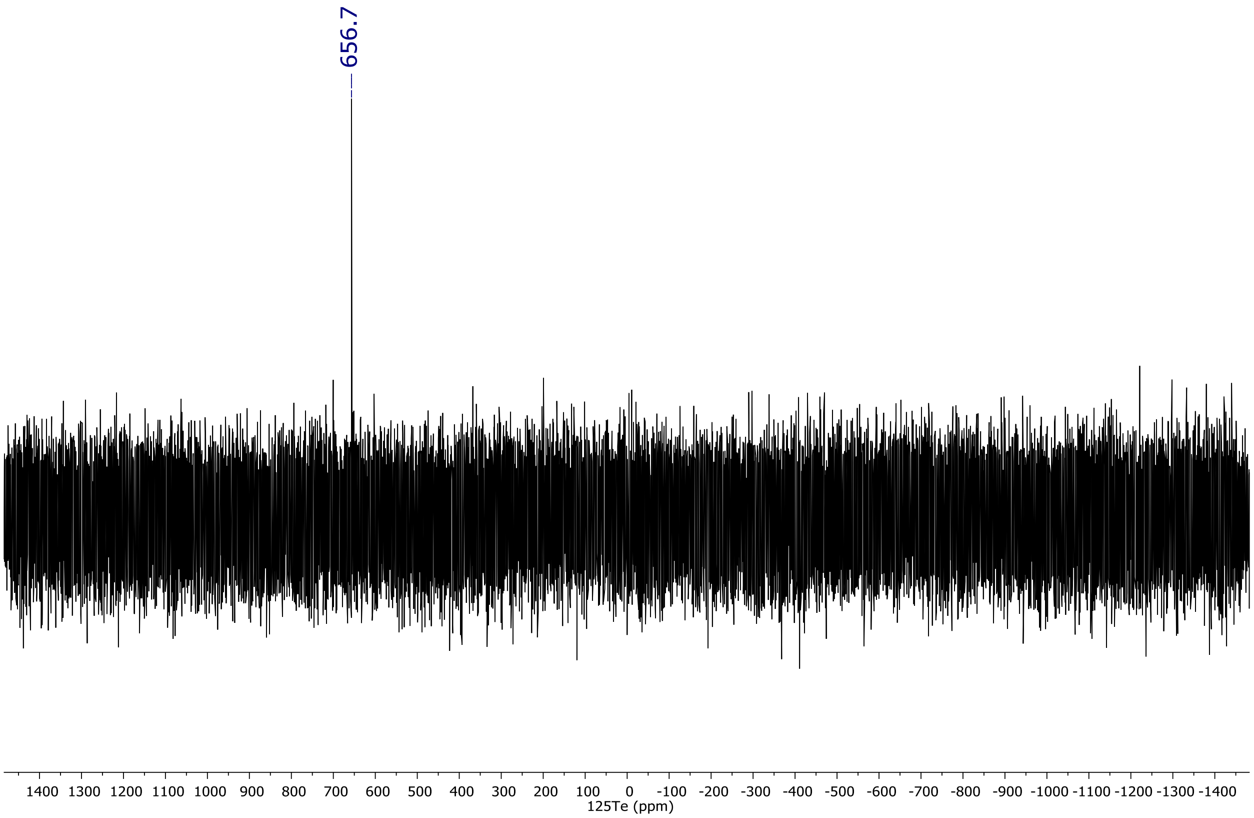

Figure S17. $^{125}Te\{^{1}H\}$ NMR spectrum of **4** in $CDCl_3$.

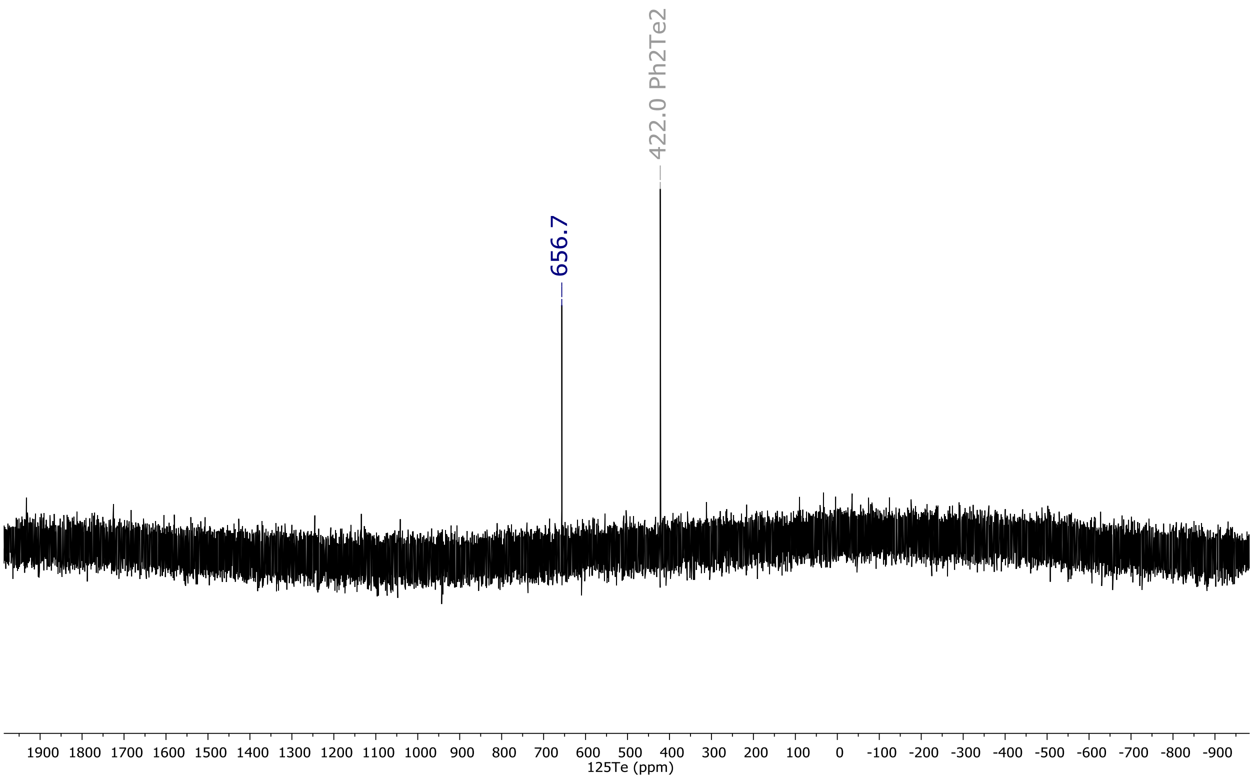

Figure S18. $^{125}Te\{^{1}H\}$ NMR spectrum of **4** in $CDCl_3$ with Ph_2Te_2 capillary.

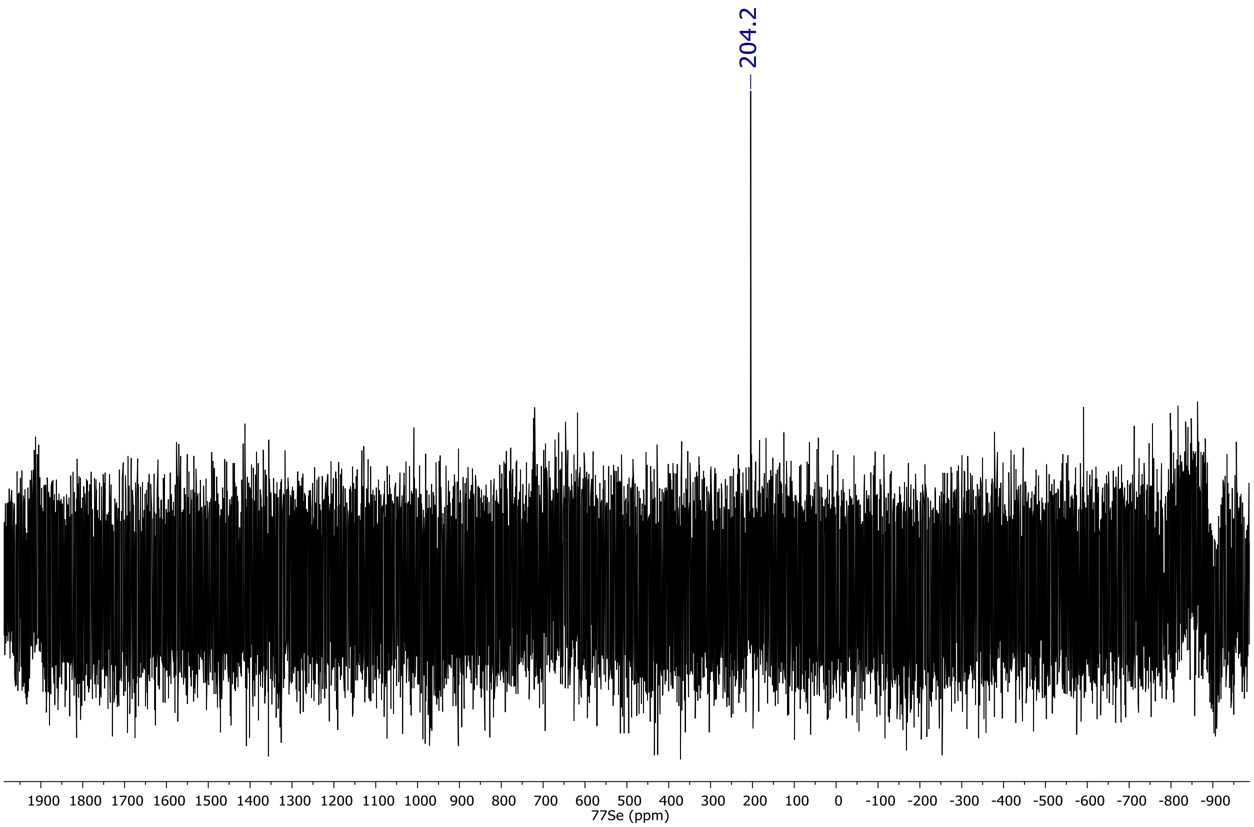

Figure S19. $^{77}Se\{^1H\}$ NMR spectrum of **4** in $CDCl_3$.

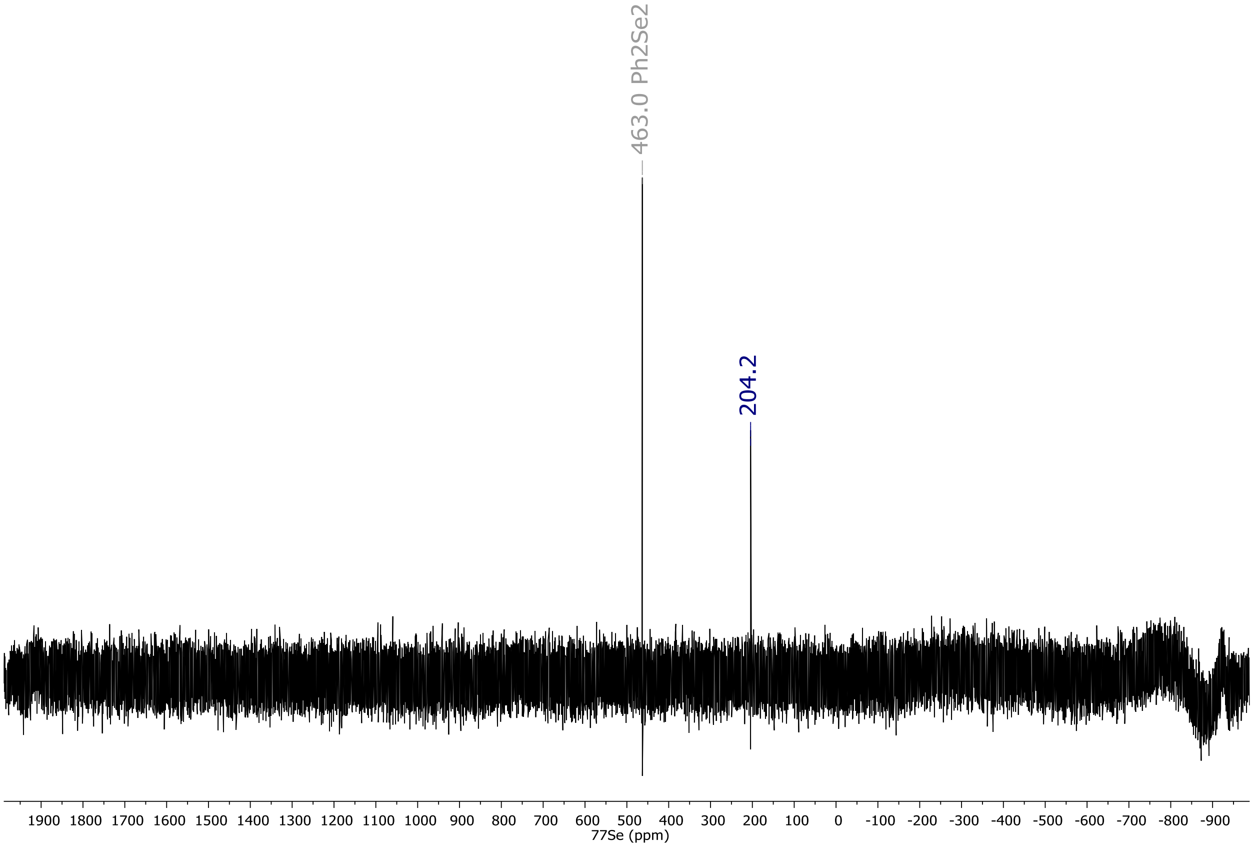

Figure S20. $^{77}Se\{^{1}H\}$ NMR spectrum of **4** in $CDCl_3$ with Ph_2Se_2 capillary.

NMR spectra for compound [5]OTf

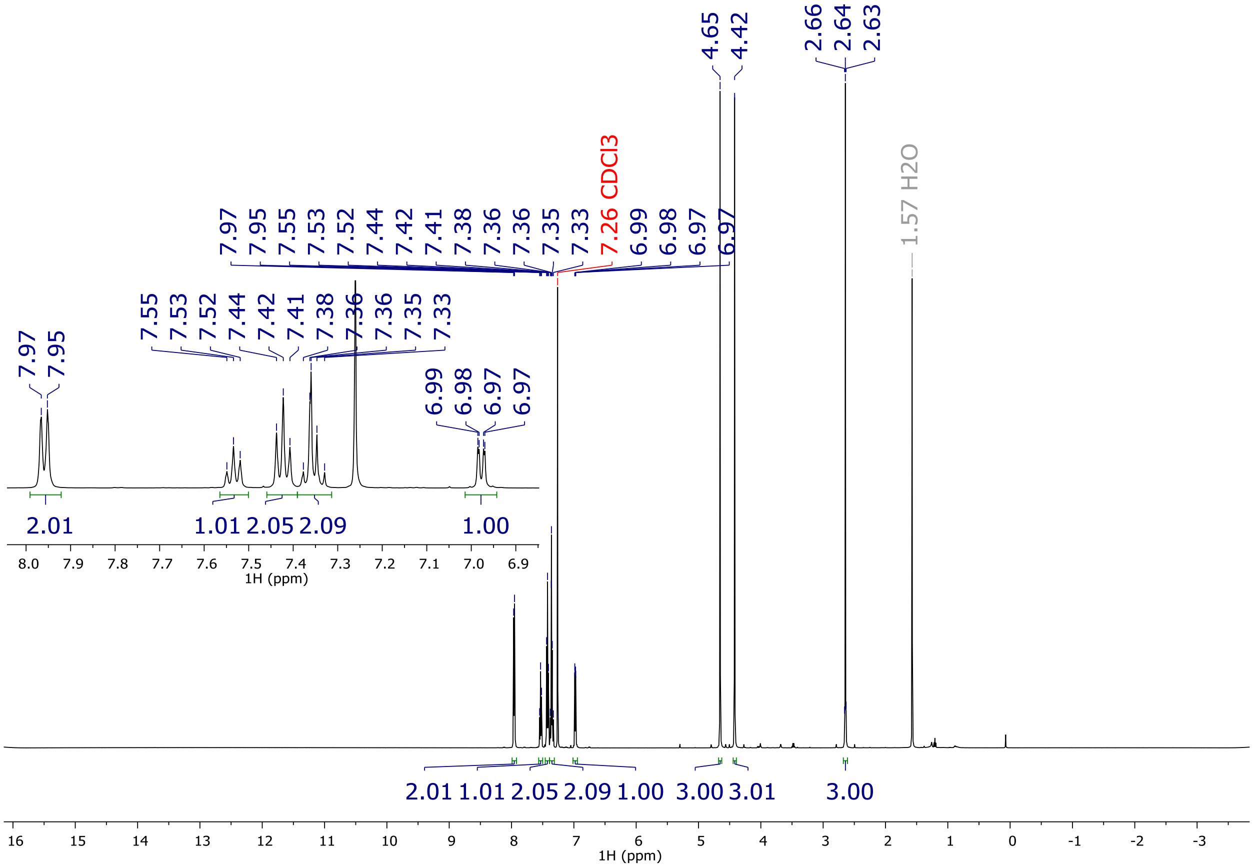

Figure S21. ^{1}H NMR spectrum of [**5**]OTf in $CDCl_3$.

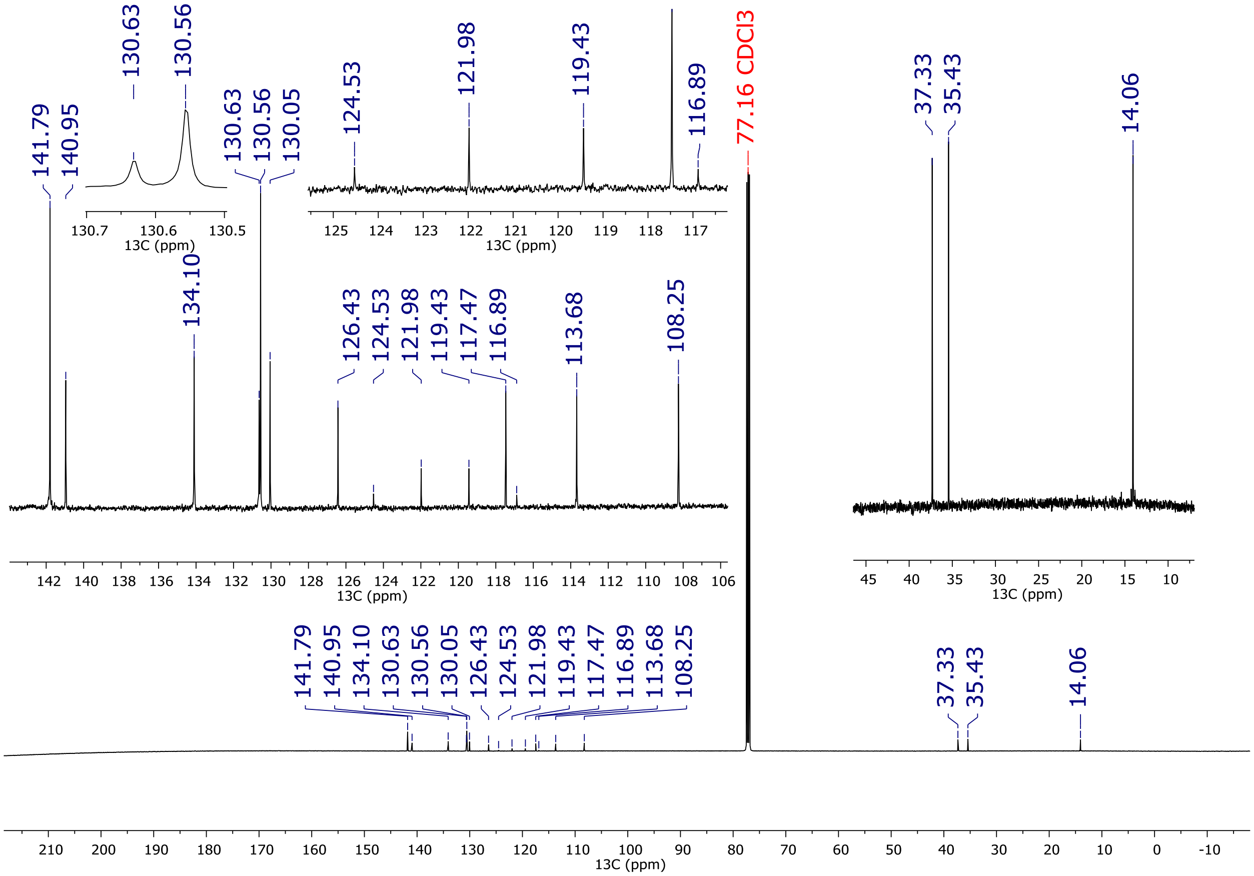

Figure S22. $^{13}C\{^{1}H\}$ NMR spectrum of [**5**]OTf in $CDCl_3$.

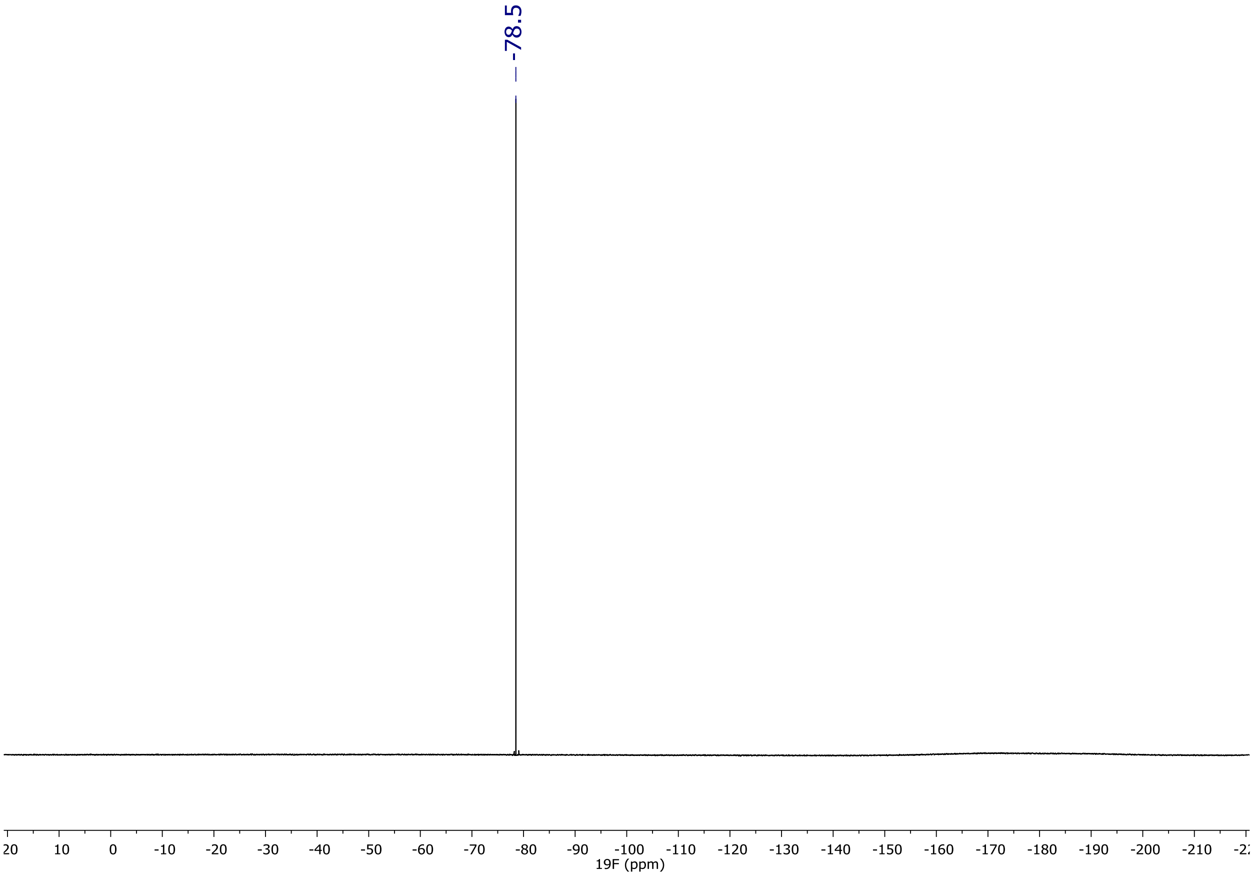

Figure S23. ^{19}F NMR spectrum of [**5**]OTf in $CDCl_3$.

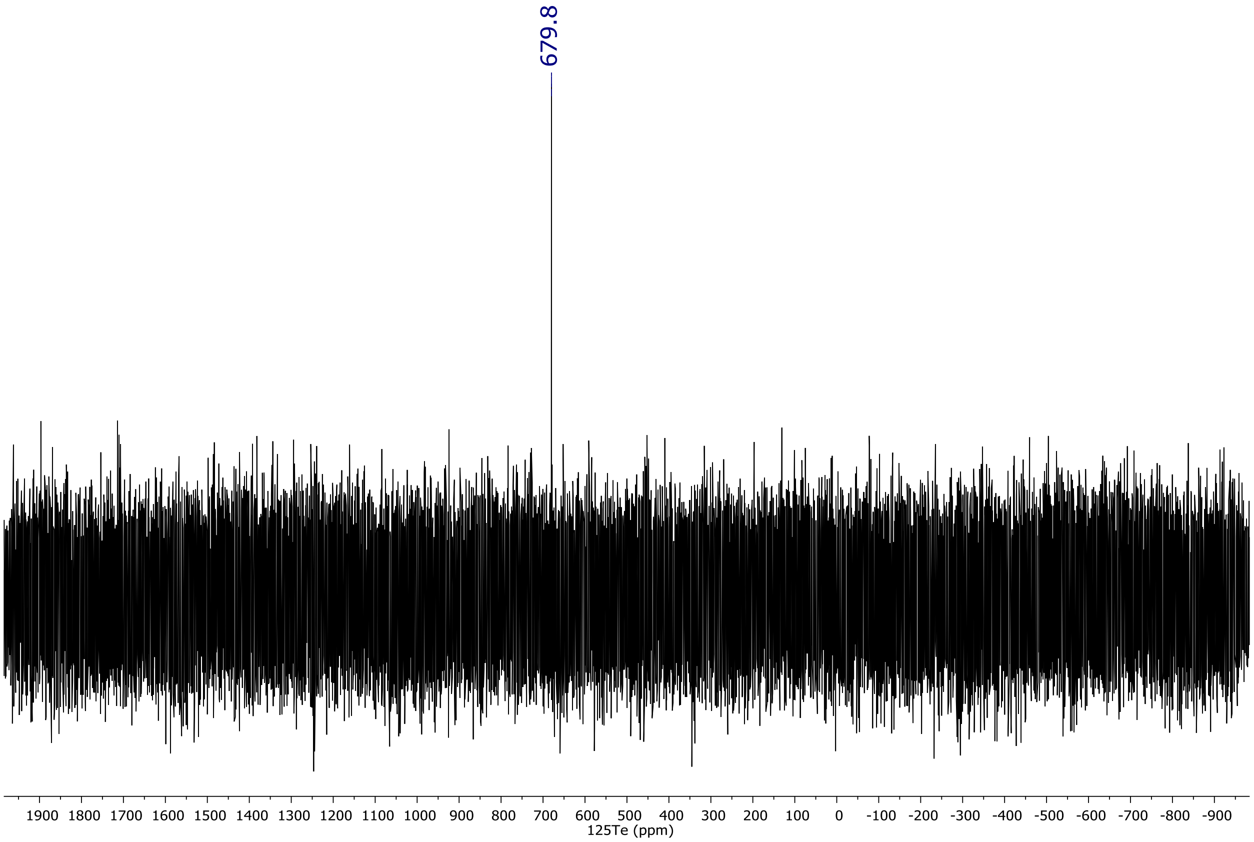

Figure S24. $^{125}Te\{^{1}H\}$ NMR spectrum of [**5**]OTf in $CDCl_3$.

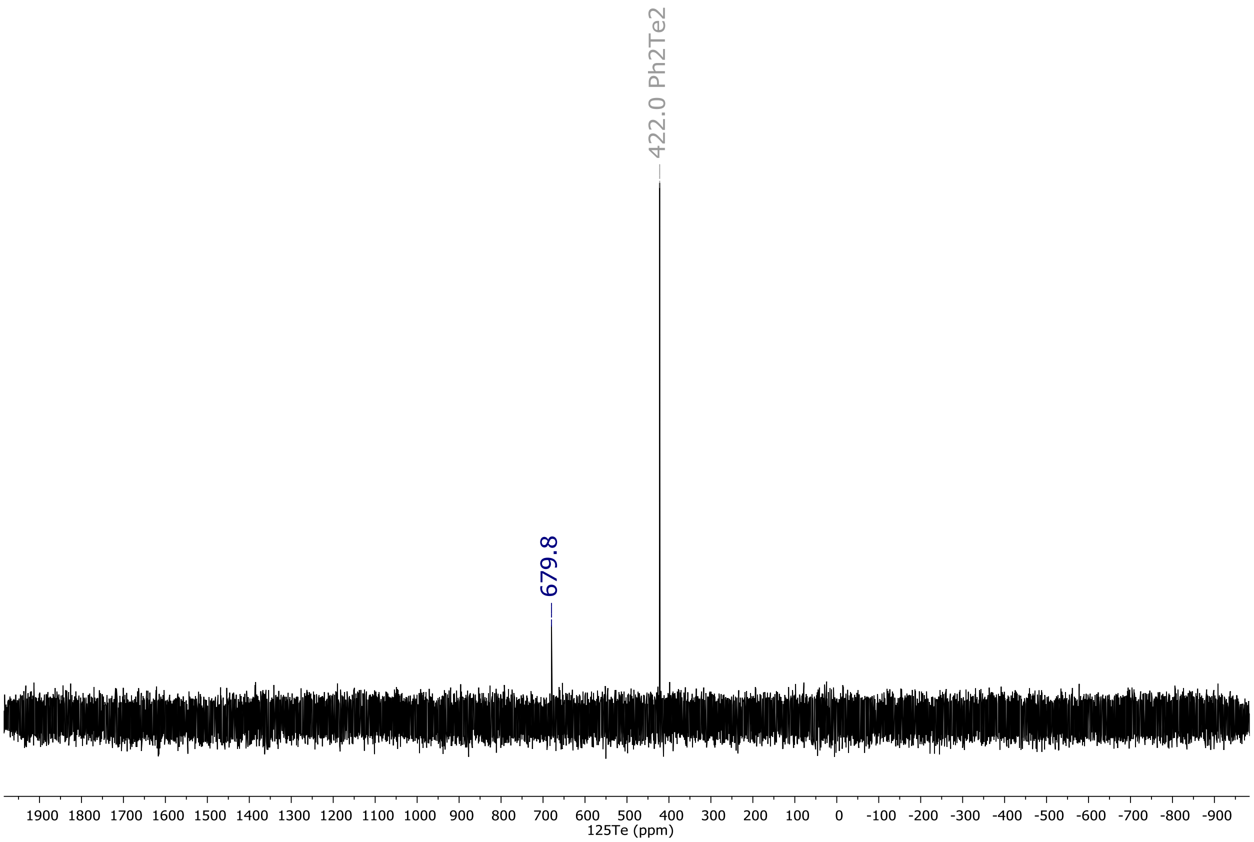

Figure S25. $^{125}Te\{^1H\}$ NMR spectrum of [**5**]OTf in $CDCl_3$ with Ph_2Te_2 capillary.

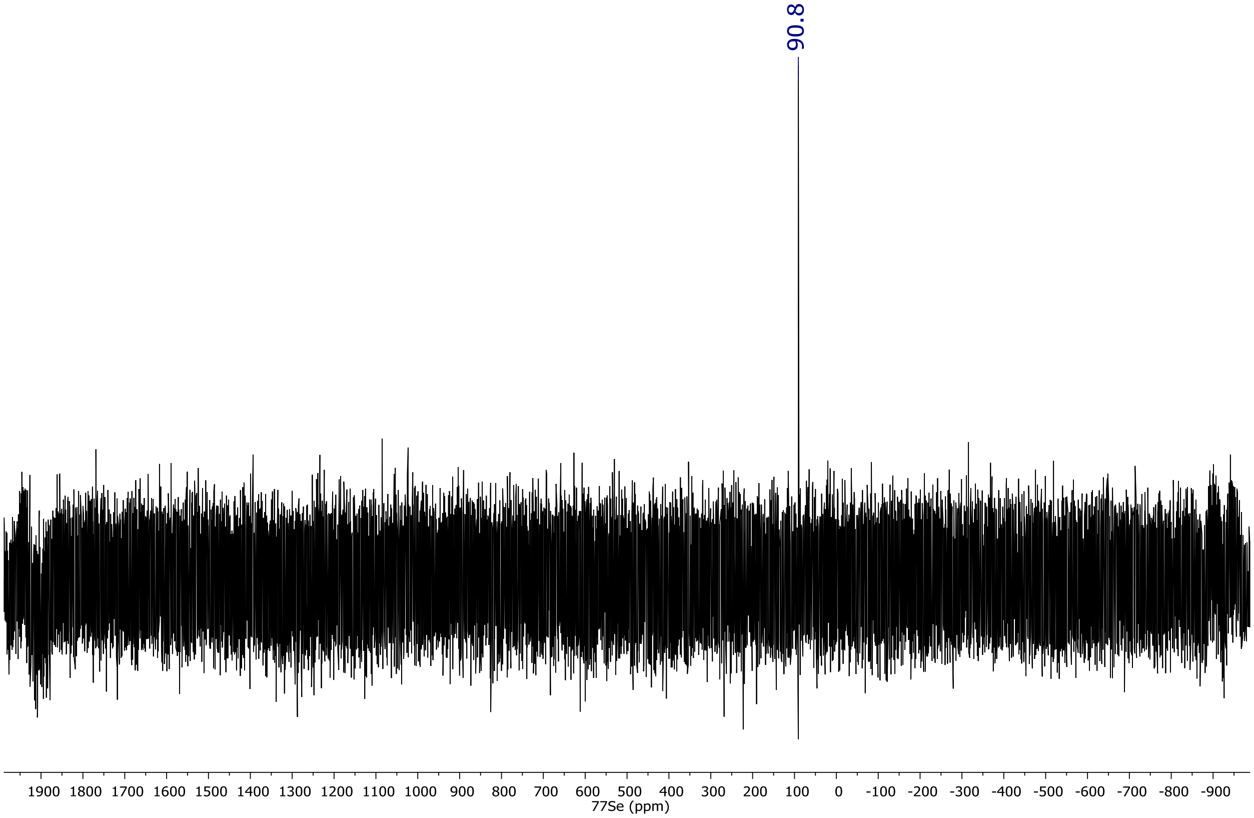

Figure S26. $^{77}Se\{^1H\}$ NMR spectrum of [**5**]OTf in $CDCl_3$.

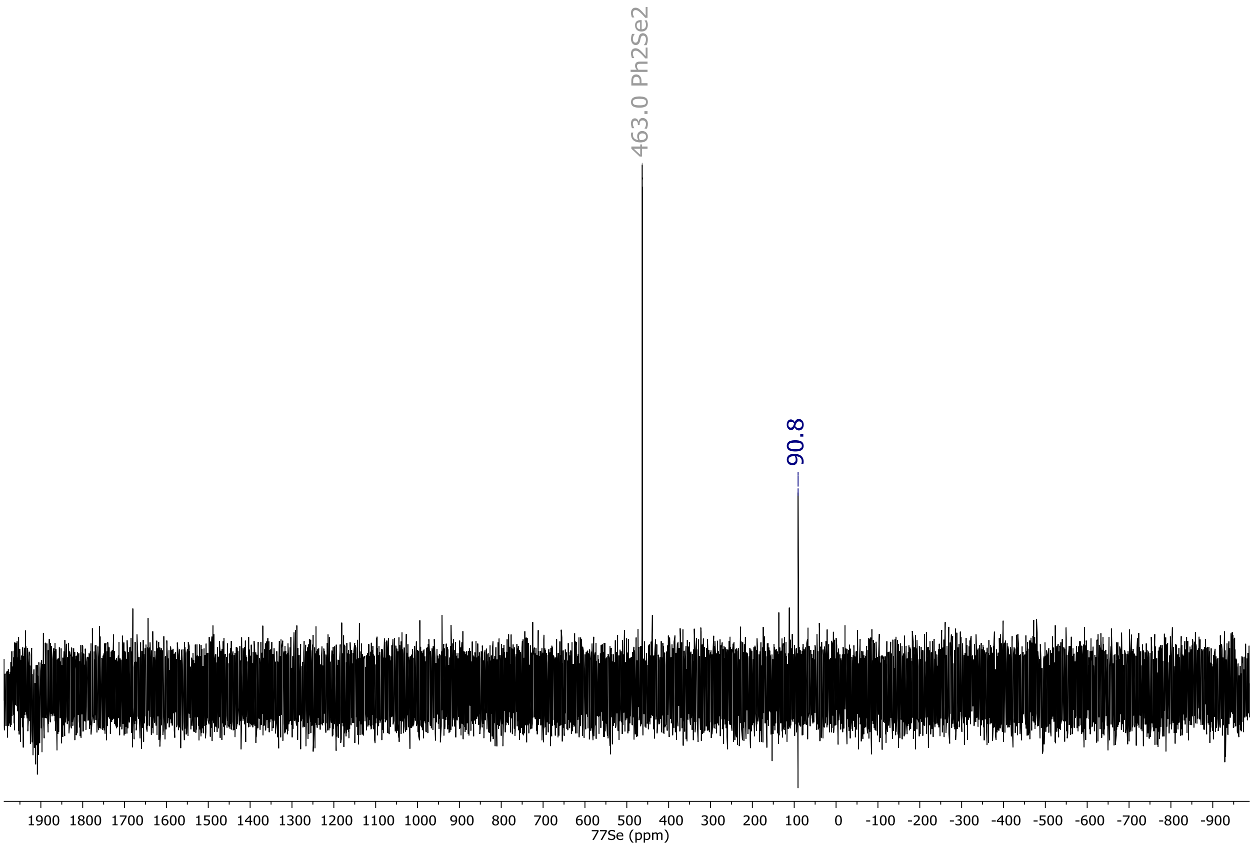

Figure S27. $^{77}Se\{^1H\}$ NMR spectrum of [**5**]OTf in $CDCl_3$ with Ph_2Se_2 capillary.

NMR spectra for compound 7

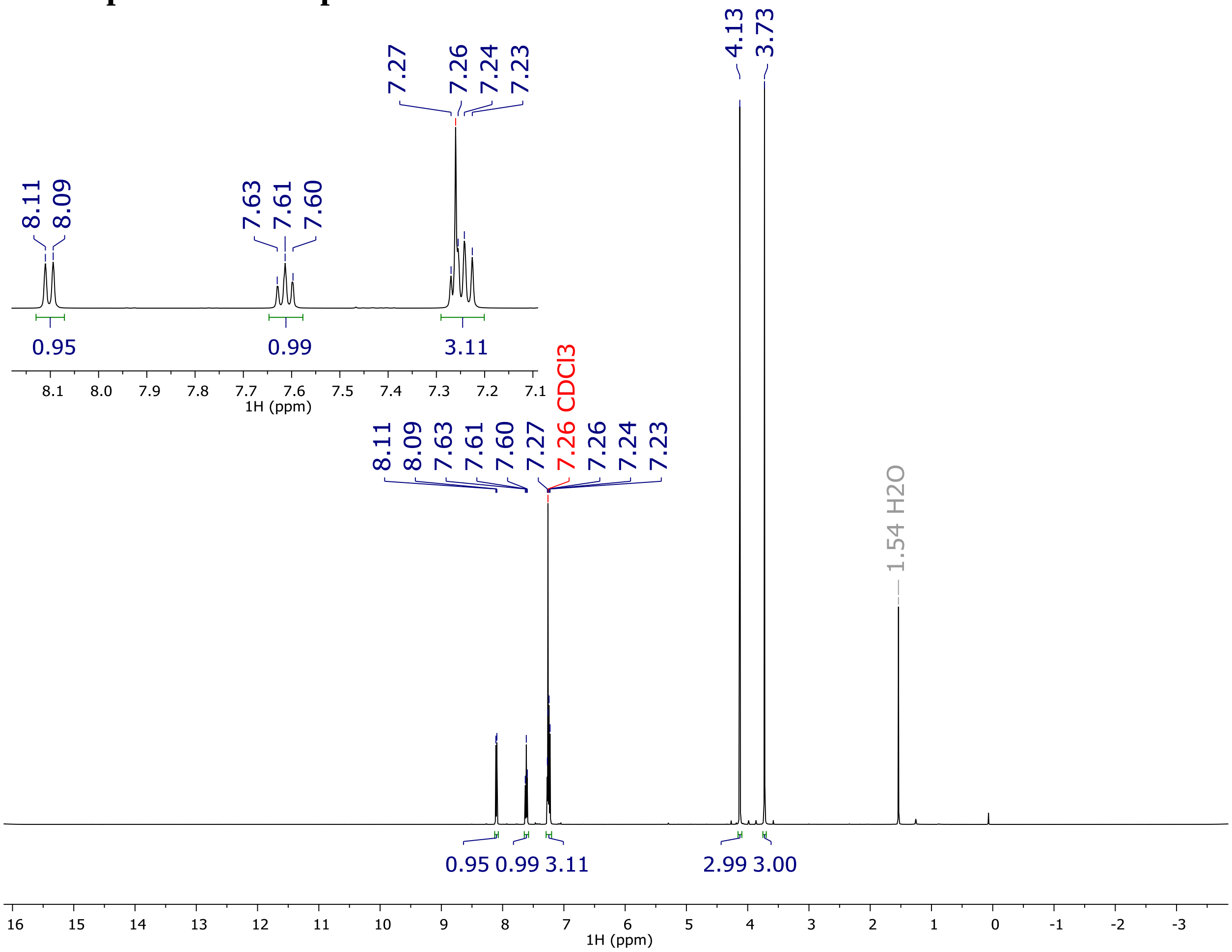

Figure S28. ^{1}H NMR spectrum of **7** in $CDCl_3$.

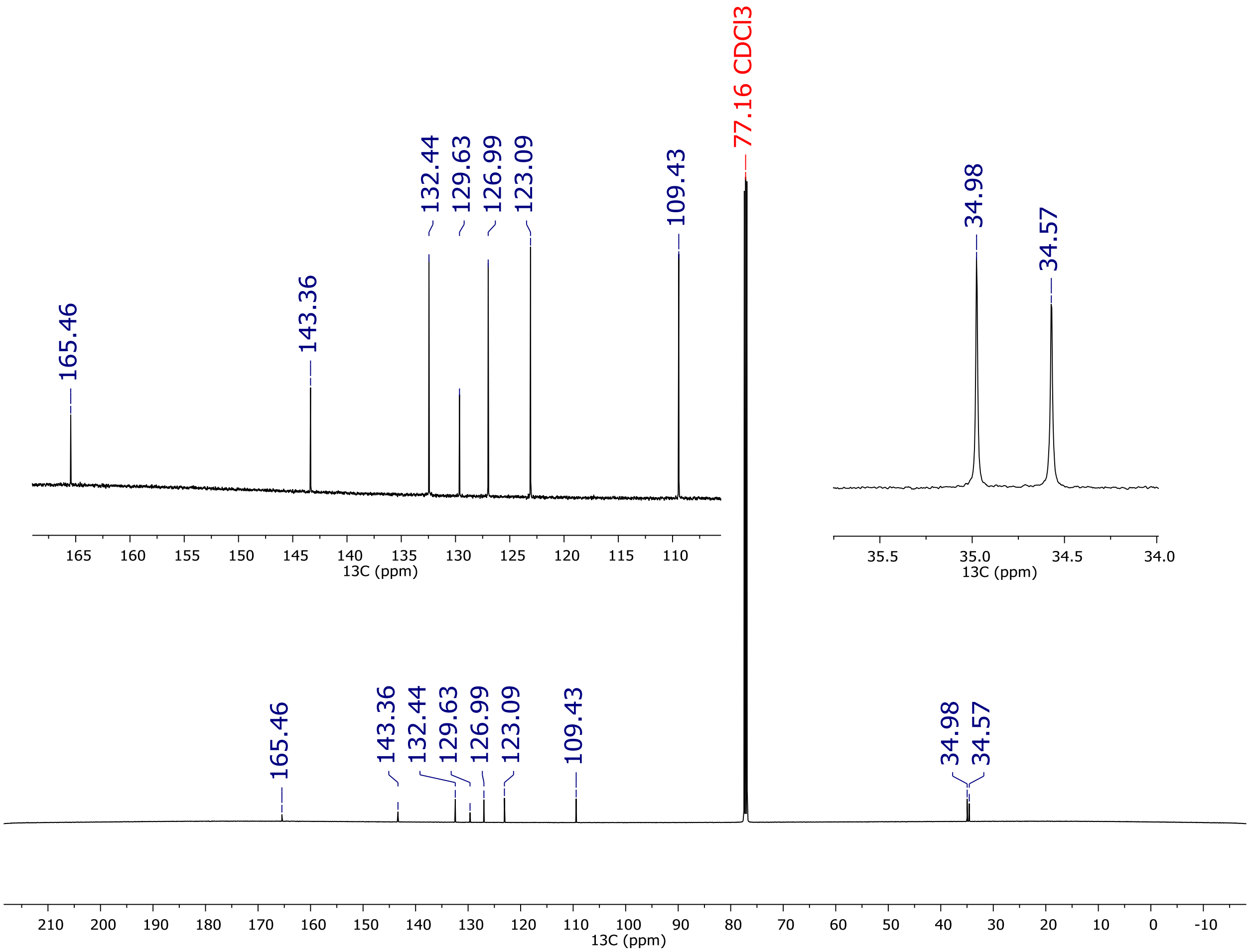

Figure S29. $^{13}C\{^{1}H\}$ NMR spectrum of **7** in $CDCl_3$.

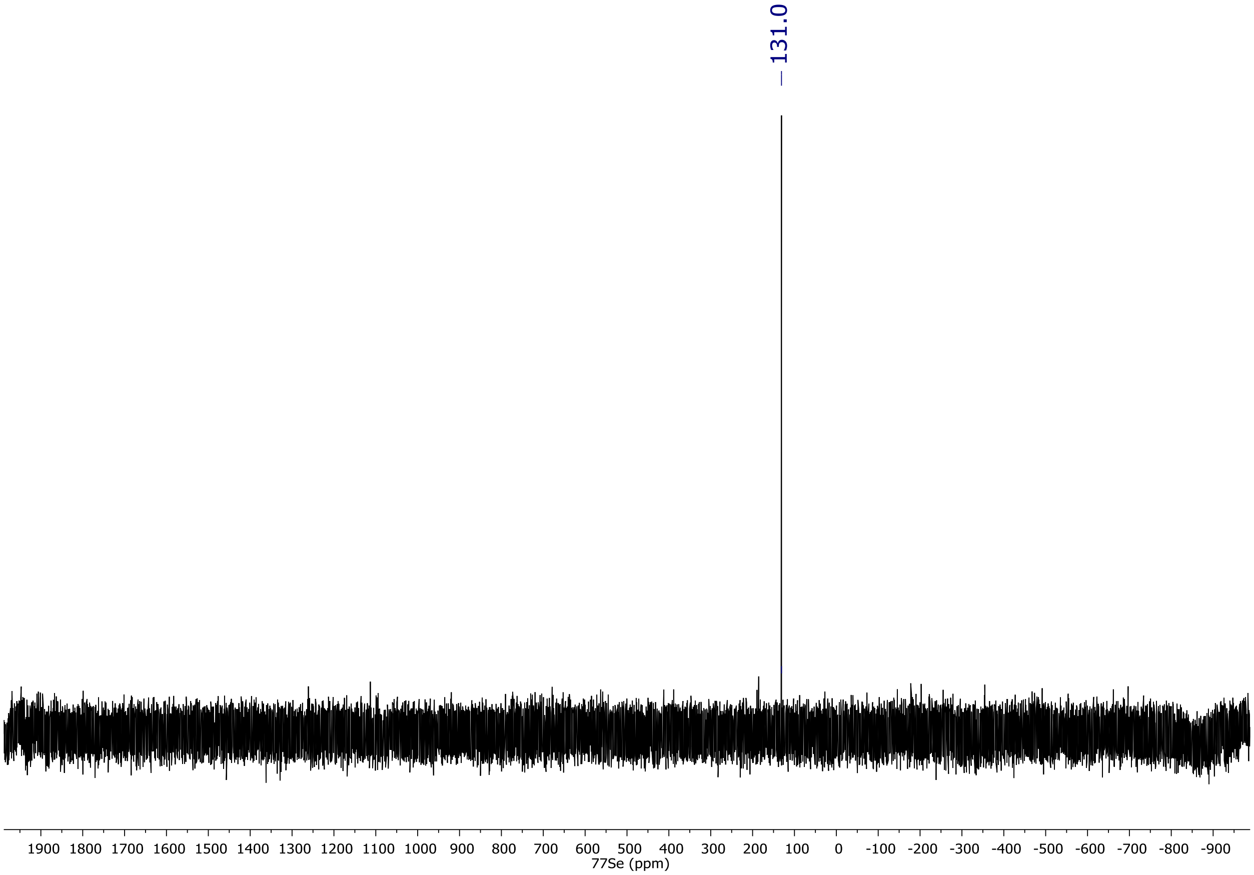

Figure S30. $^{77}Se\{^{1}H\}$ NMR spectrum of **7** in $CDCl_3$.

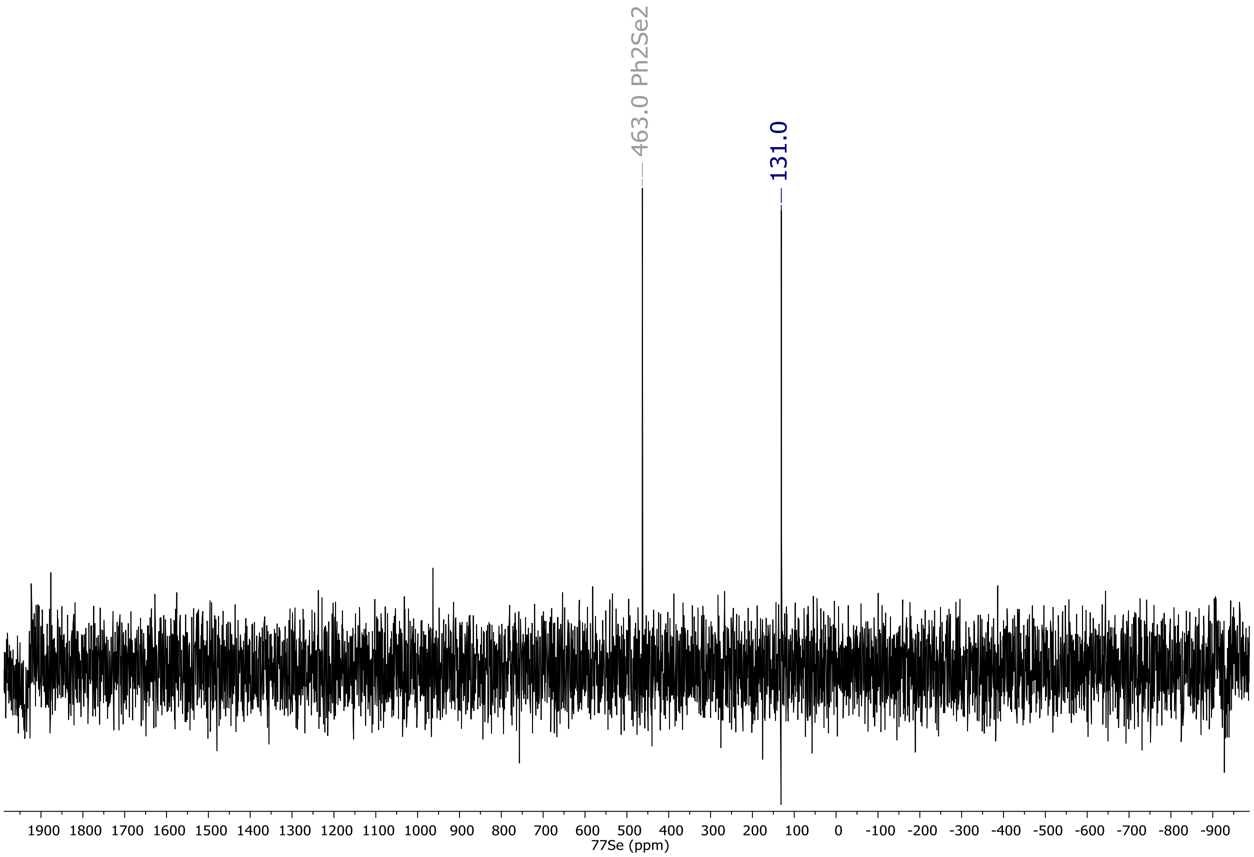

Figure S31. $^{77}Se\{^1H\}$ NMR spectrum of **7** in $CDCl_3$ with Ph_2Se_2 capillary.

NMR spectra for compound [8]OTf

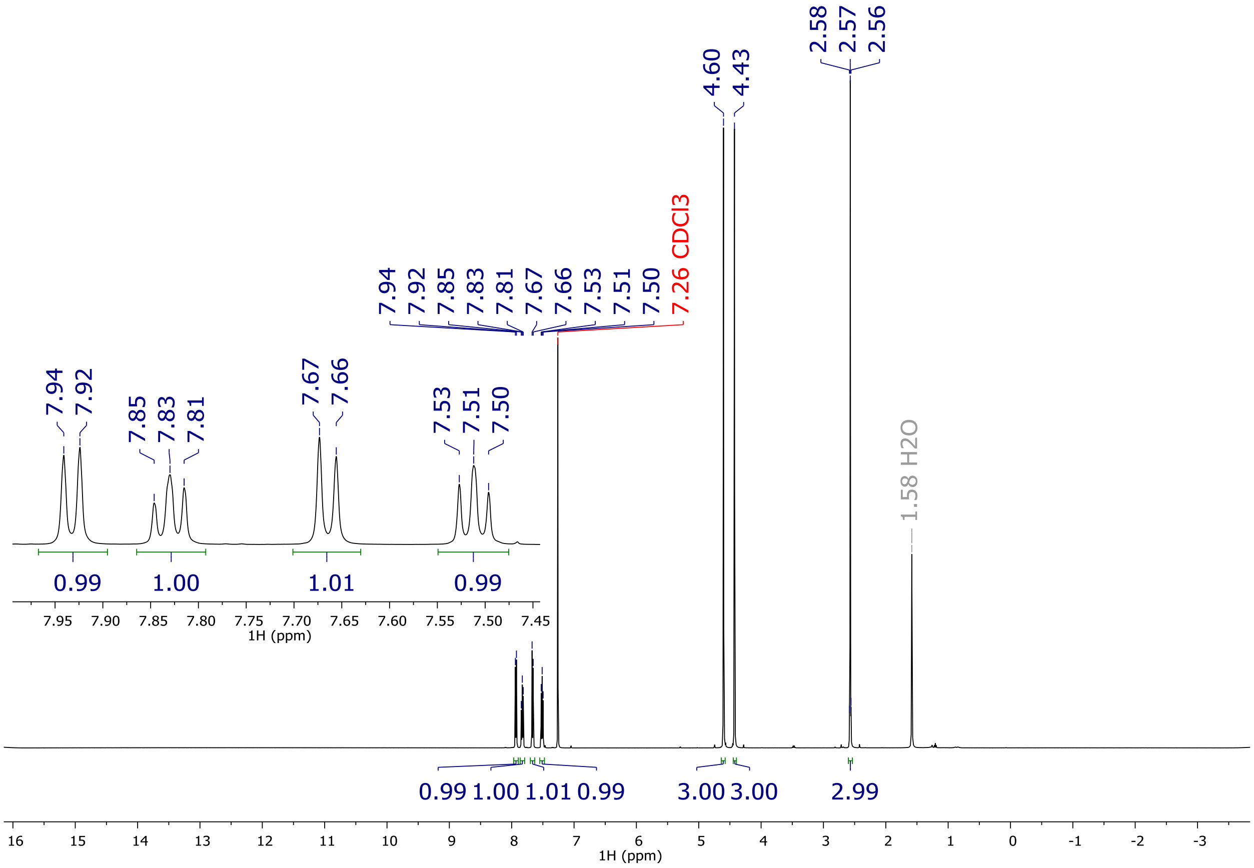

Figure S32. ^{1}H NMR spectrum of [**8**]OTf in $CDCl_3$.

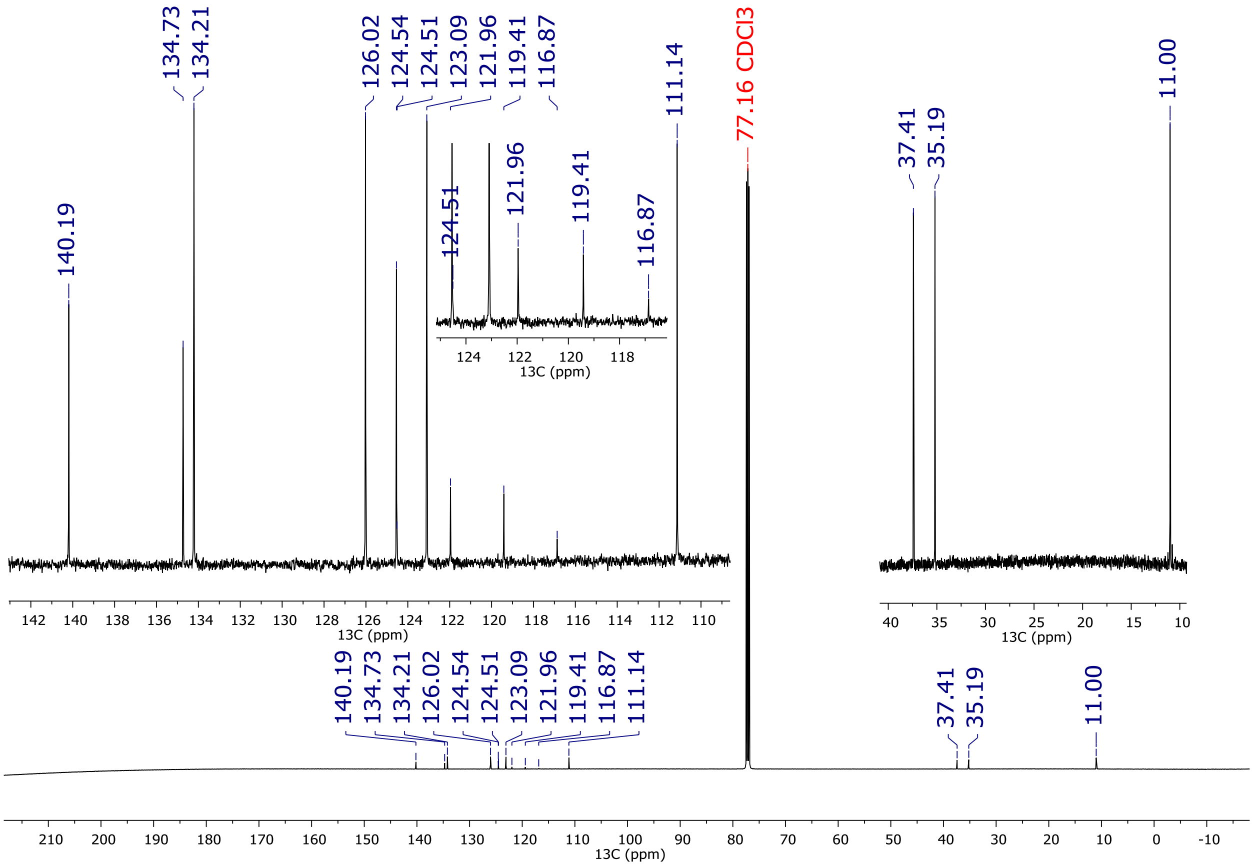

Figure S33. $^{13}C\{^{1}H\}$ NMR spectrum of [**8**]OTf in $CDCl_3$.

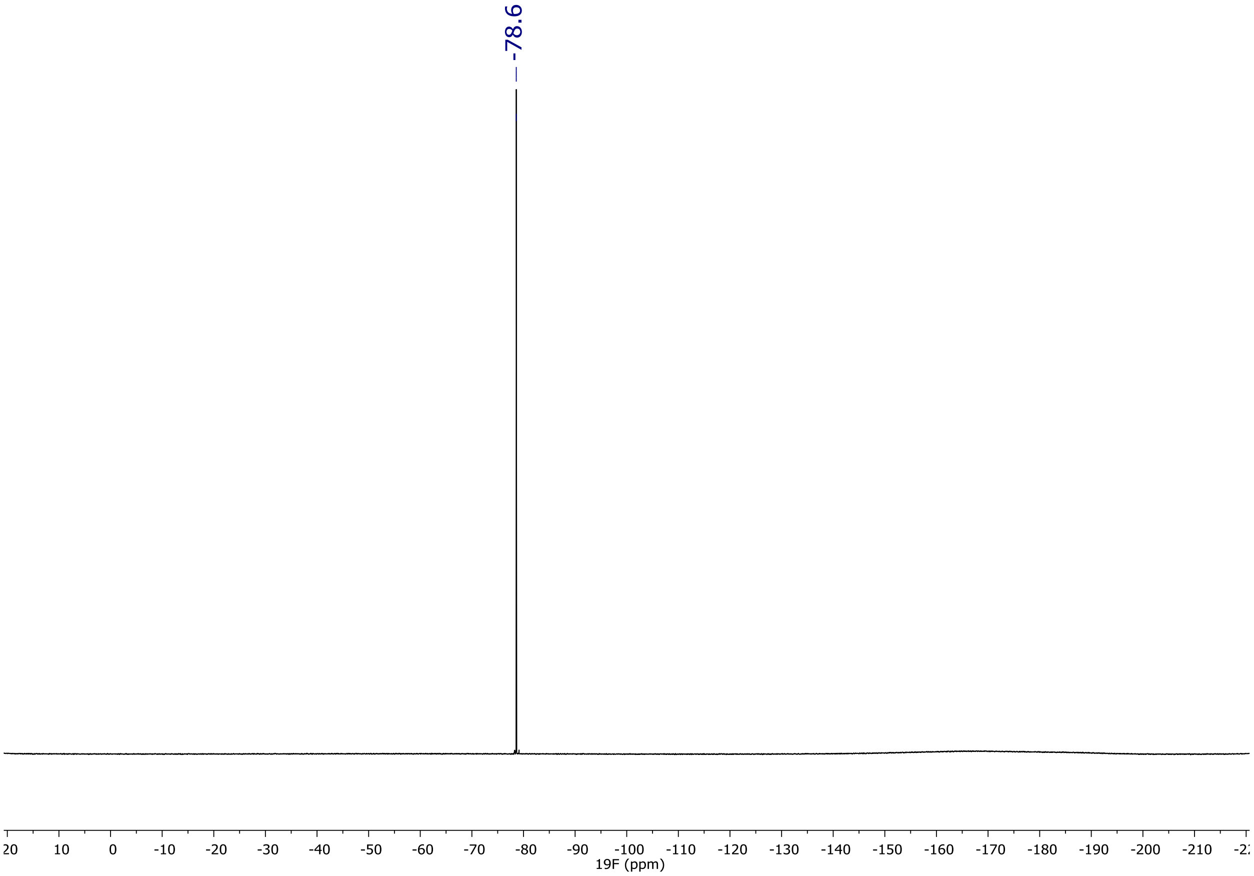

Figure S34. ^{19}F NMR spectrum of [**8**]OTf in $CDCl_3$.

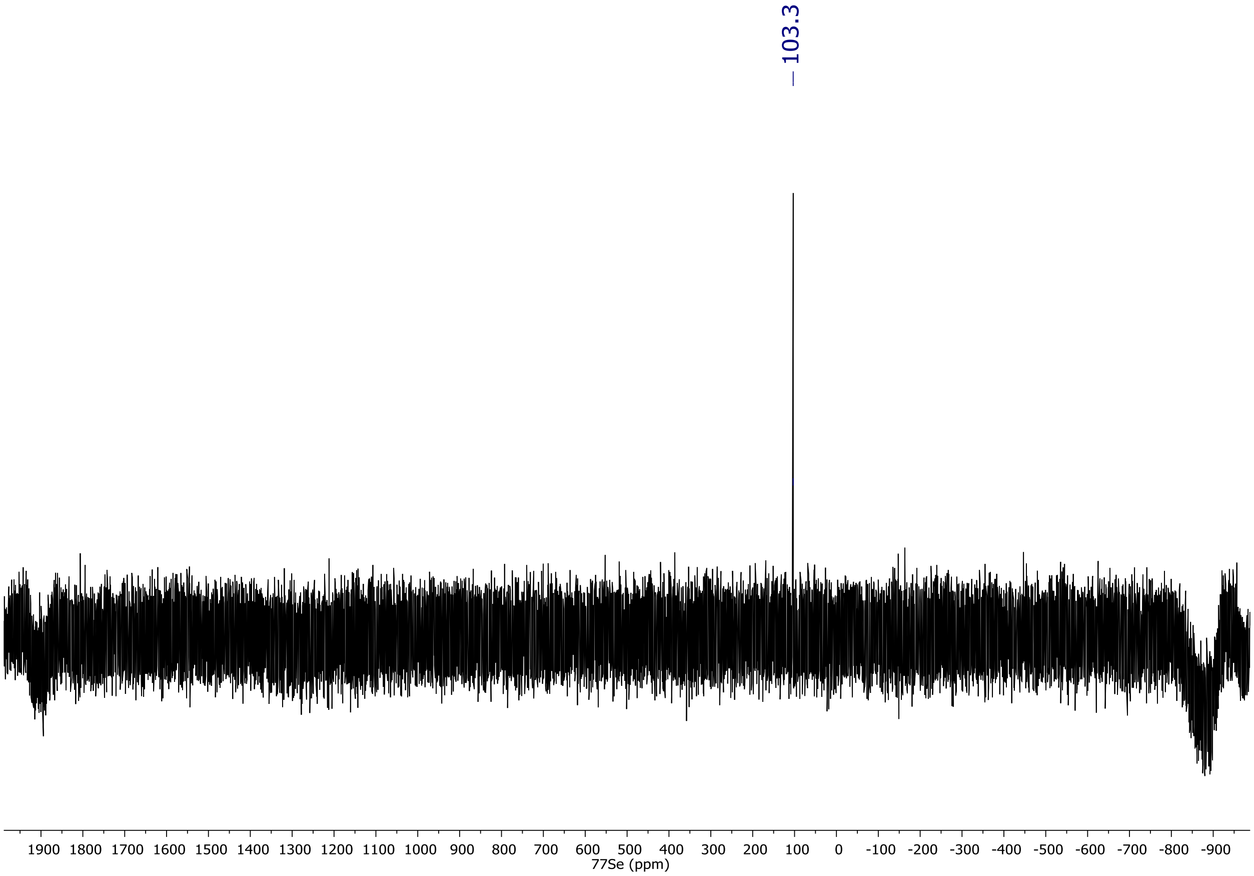

Figure S35. $^{77}Se\{^1H\}$ NMR spectrum of [**8**]OTf in $CDCl_3$.

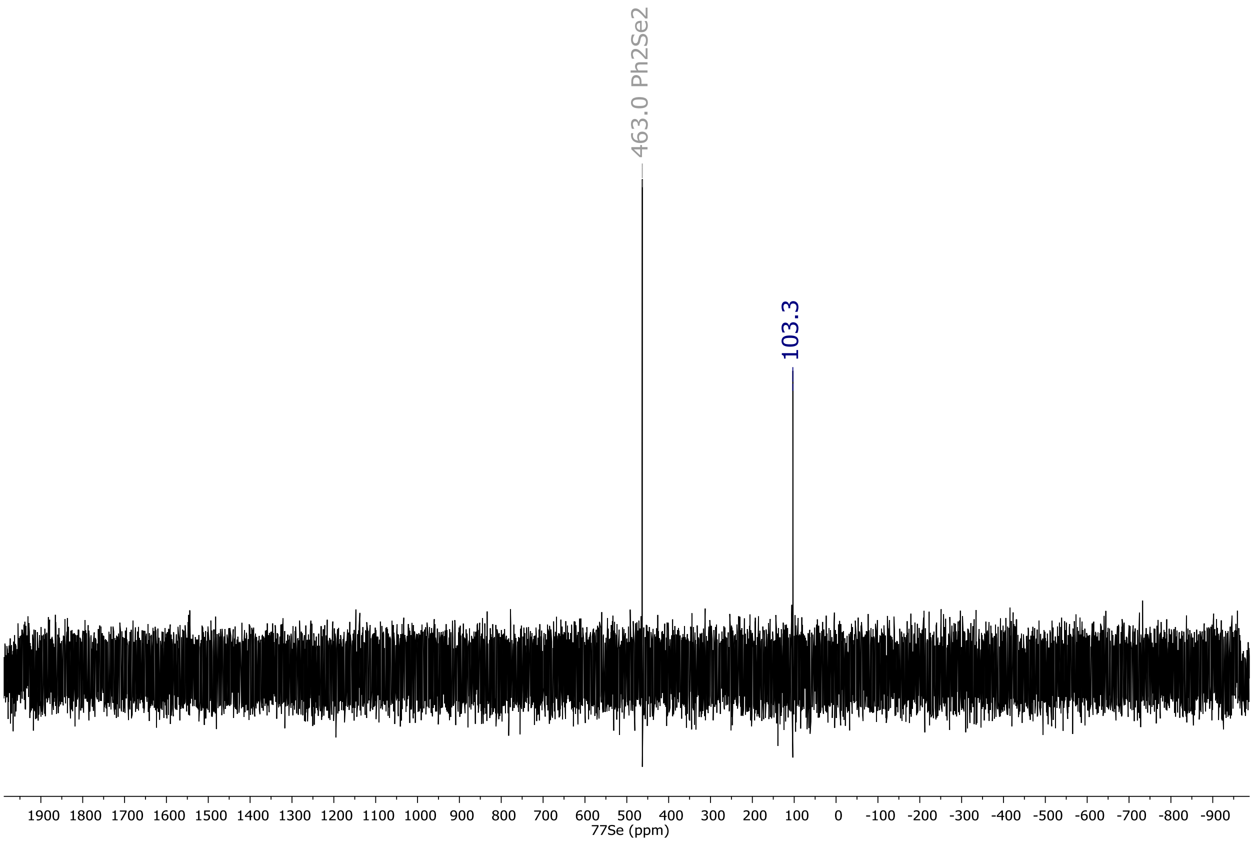

Figure S36. $^{77}Se\{^{1}H\}$ NMR spectrum of [**8**]OTf in $CDCl_3$ with Ph_2Se_2 capillary.

NMR Spectra for Catalytic Studies

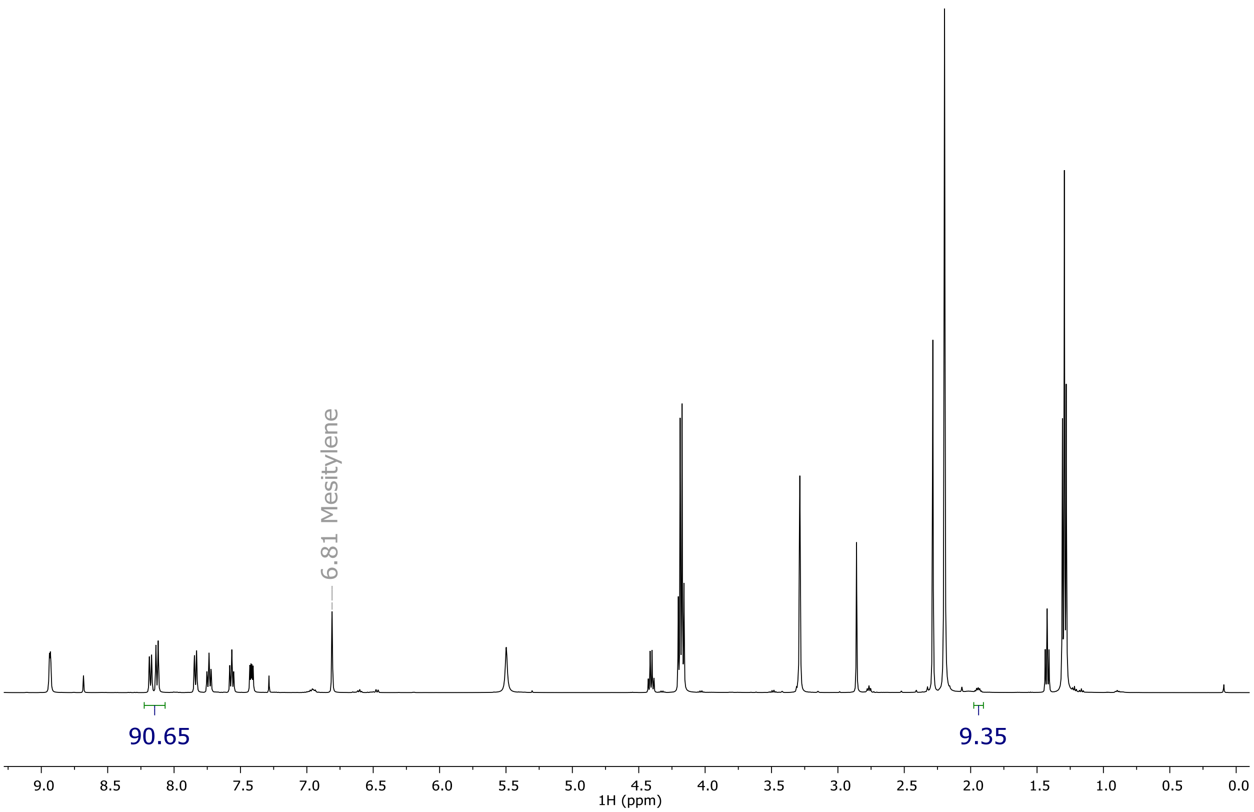

Figure S37. ^{1}H NMR spectrum obtained with no catalyst (blank). This spectrum was collected 20 h after mixing. Integration of the signal indicated a conversion of 9%.

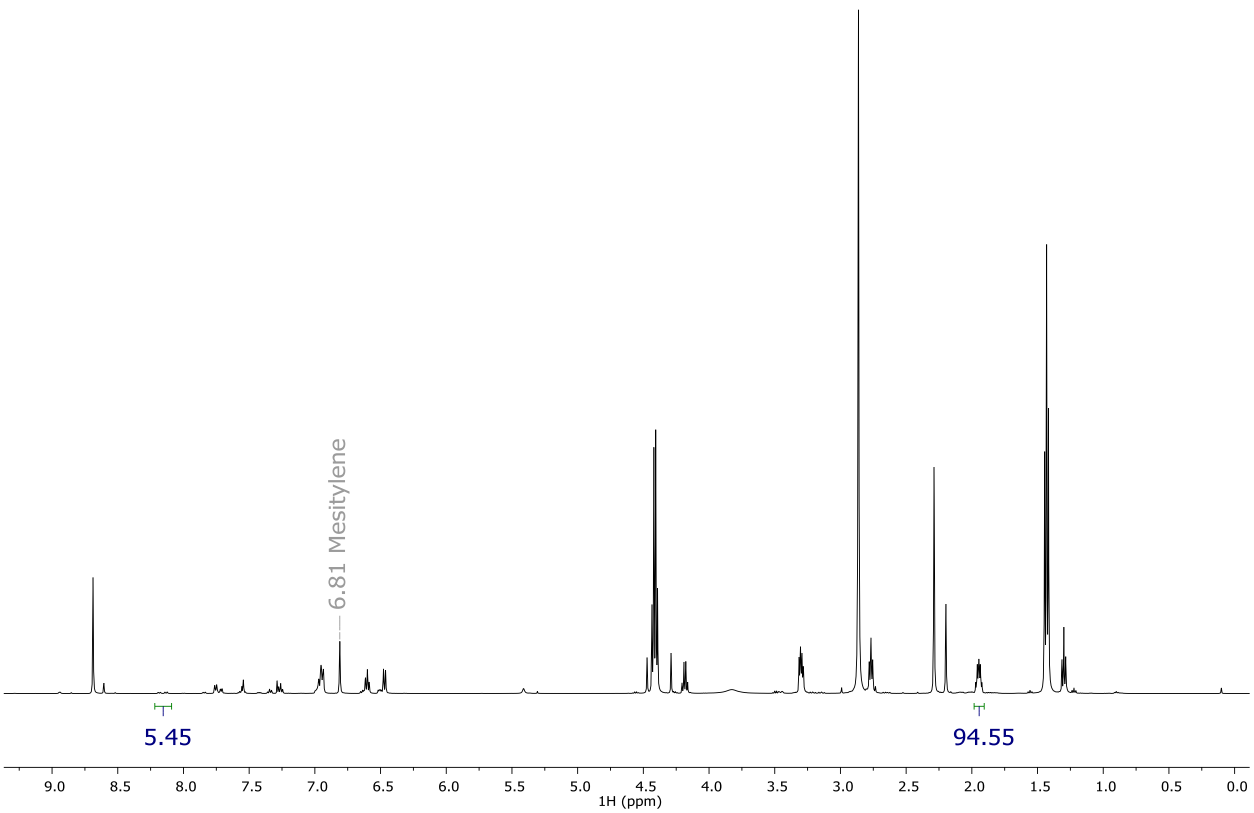

Figure S38. ^{1}H NMR spectrum obtained with [**3**]OTf as catalyst. This spectrum was collected 20 h after mixing. Integration of the signal indicated a conversion of 95%.

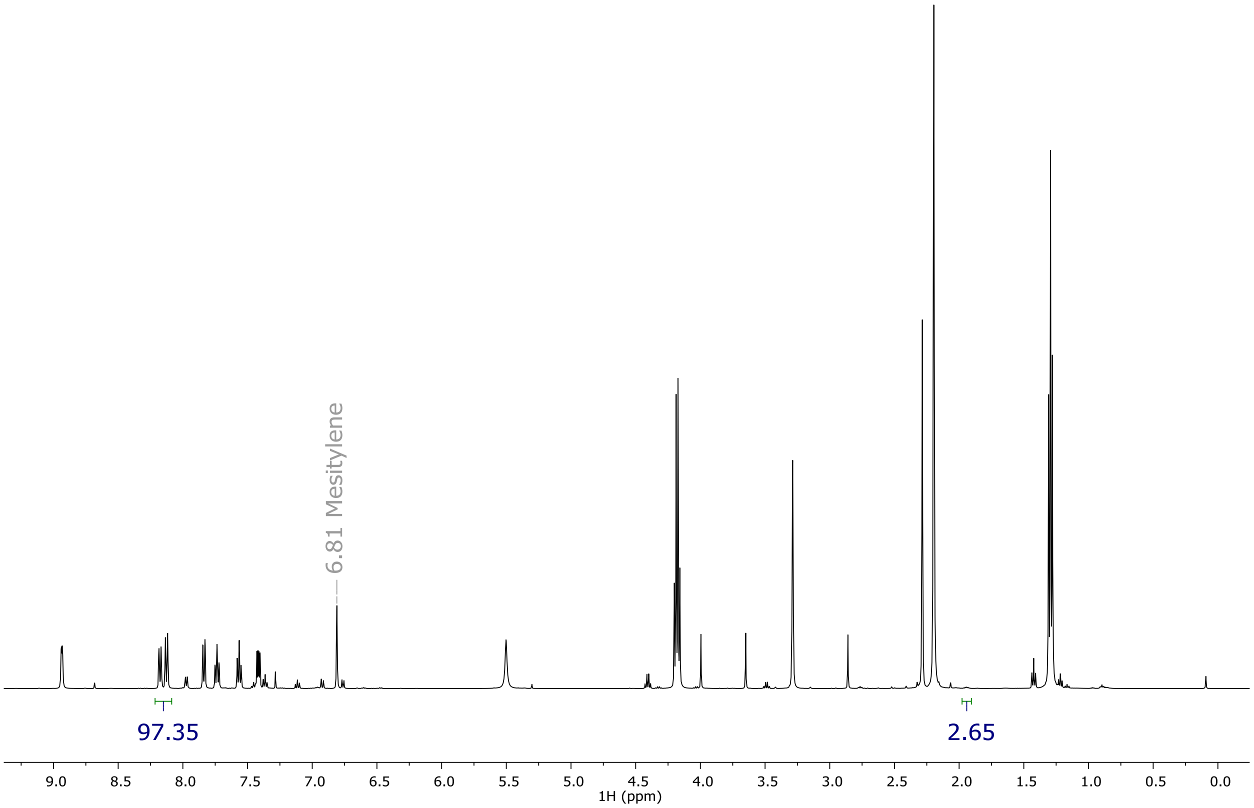

Figure S39. ^1H NMR spectrum obtained with **4** as catalyst. This spectrum was collected 20 h after mixing. Integration of the signal indicated a conversion of 3%.

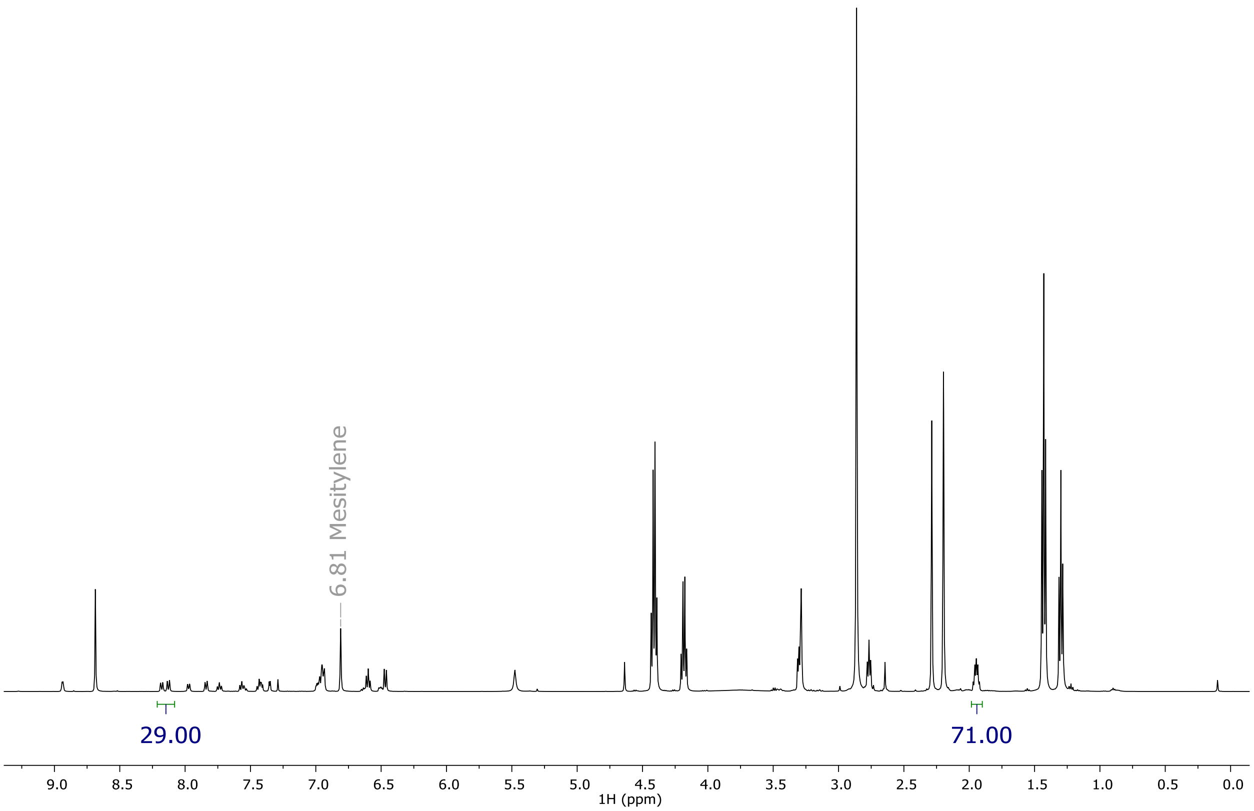

Figure S40. ^{1}H NMR spectrum obtained with [**5**]OTf as catalyst. This spectrum was collected 20 h after mixing. Integration of the signal indicated a conversion of 71%.

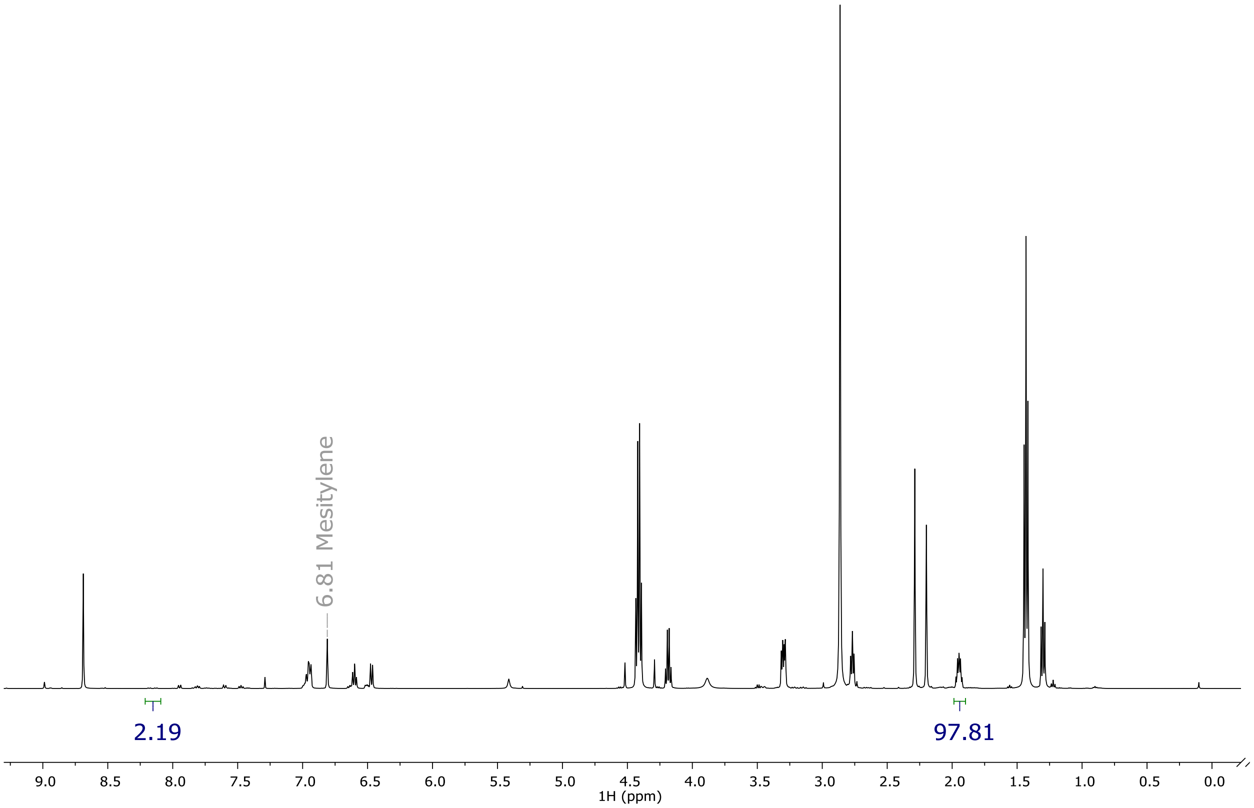

Figure S41. ^{1}H NMR spectrum obtained with [**6**]OTf (same as **C**) as catalyst. This spectrum was collected 20 h after mixing. Integration of the signal indicated a conversion of 98%.

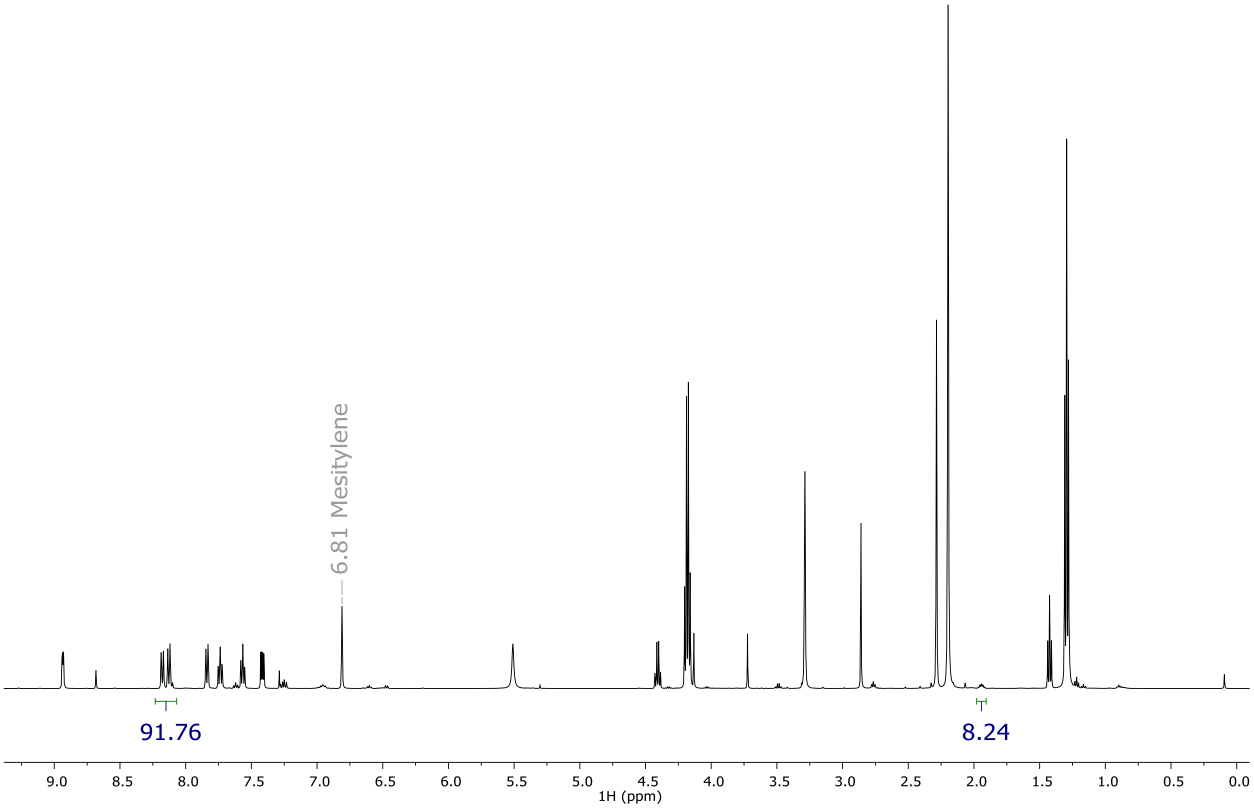

Figure S42. ^{1}H NMR spectrum obtained with **7** as catalyst. This spectrum was collected 20 h after mixing. Integration of the signal indicated a conversion of 8%.

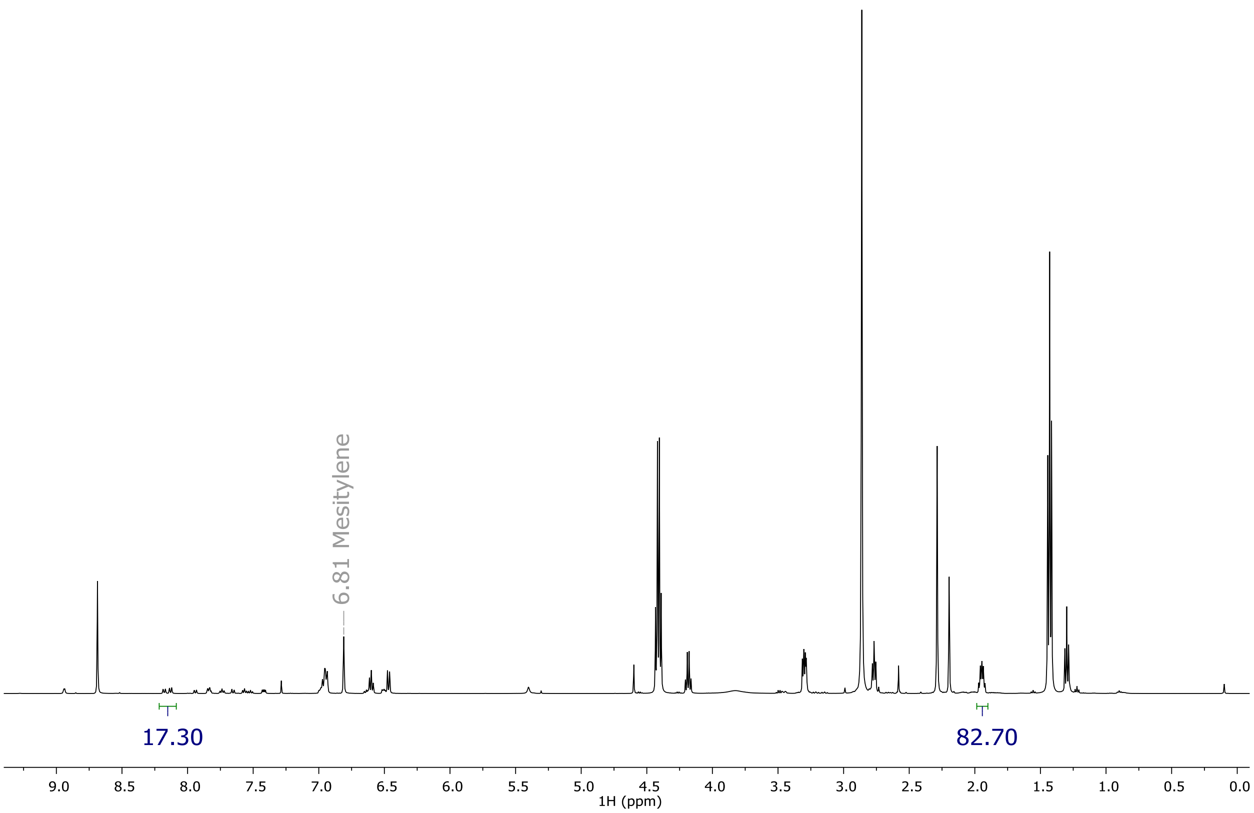

Figure S43. ^{1}H NMR spectrum obtained with [**8**]OTf as catalyst. This spectrum was collected 20 h after mixing. Integration of the signal indicated a conversion of 83%.

Computational Details

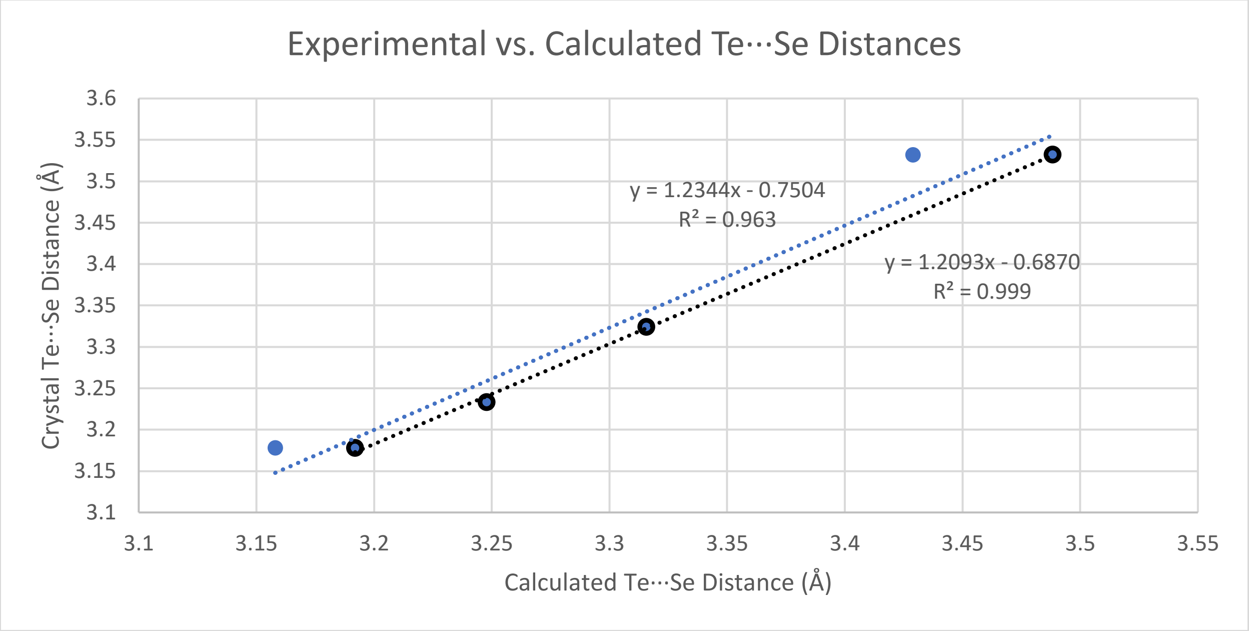

Graph S1. Correlation between the experimental Te···Se distances in the crystal structures and the distances calculated using the level of theory described in the Computational Details. The blue dots and the associated blue trendline include all bond lengths, including the two different ones present in the crystal structures of [**5**]OTf and **D**. The black circles around the blue dots and the associated black trendline remove the shorter of the two distances in the [**5**]OTf and **D** unit cells, showing better correlation between the experimental and calculated values.

Table S1. Natural Population Analysis (NPA) charges on chalcogen centers in molecules investigated.

Compound	Te charge	Se charge
**2**	+0.51	—
$\mathbf{3}^{+}$	+0.58	—
**4**	+0.59	-0.17
$\mathbf{5}^{+}$	+0.59	+0.37
$\mathbf{6}^{+}$	—	—
**7**	—	-0.21
$\mathbf{8}^{+}$	—	+0.38
**D**	+0.52	+0.40
**E**	+0.52	+0.39

Optimized geometries

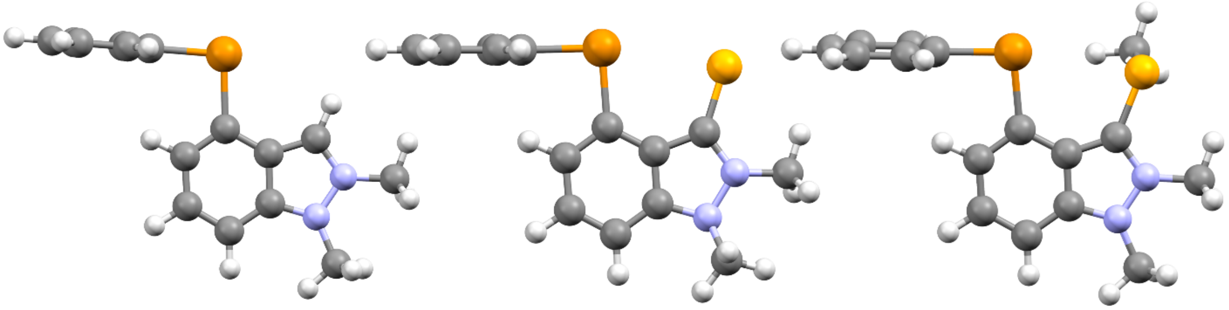

3^+ 4 5^+

Figure S44. Optimized geometries of **3**$^+$, **4**, and **5**$^+$.

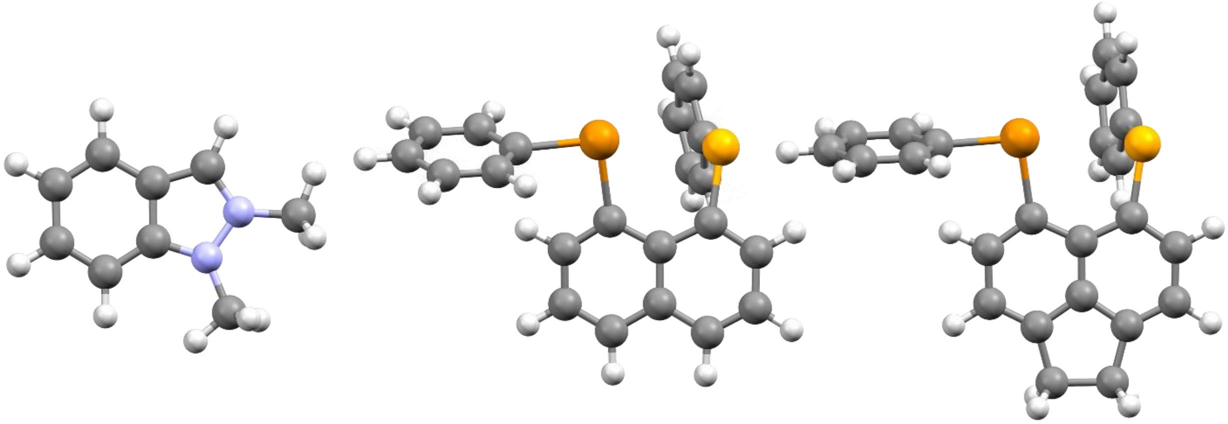

6$^+$ (same as **C**) **D** **E**

Figure S45. Optimized geometries of **6**$^+$ (same as **C**), **D**, and **E**.

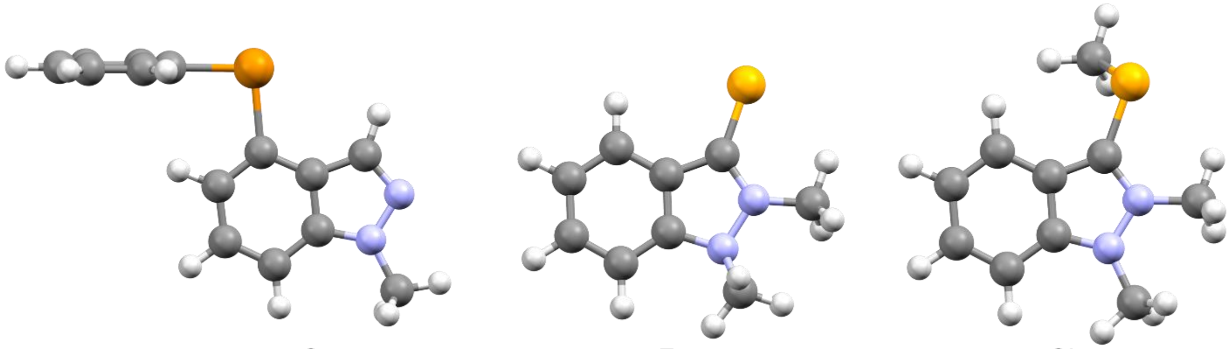

2 **7** **8**$^+$

Figure S46. Optimized geometries of **2**, **7**, and **8**$^+$.

NBO interactions in D and E

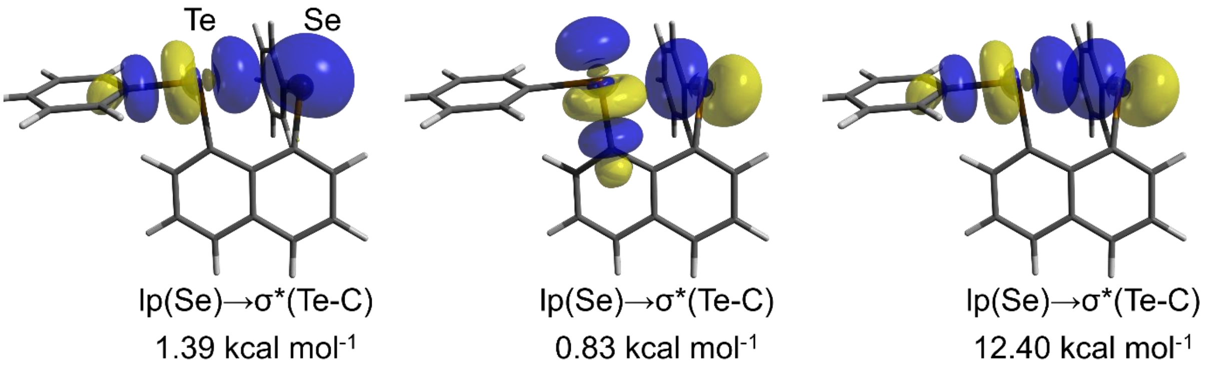

Figure S47. All lp(Se)→σ*(Te-C) donor-acceptor interactions (isovalue = 0.05) present in **D**.

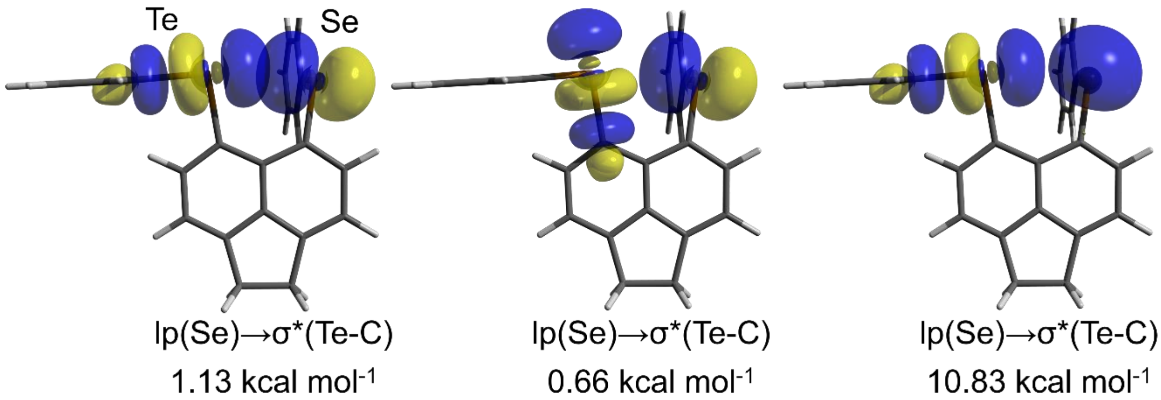

Figure S48. All lp(Se)→σ*(Te-C) donor-acceptor interactions (isovalue = 0.05) present in **E**.
